# Design, Synthesis, Biological Evaluation, 2D-QSAR Modeling, and Molecular Docking Studies of Novel 1*H*-3-Indolyl Derivatives as Significant Antioxidants

**DOI:** 10.3390/ijms221910396

**Published:** 2021-09-27

**Authors:** Maged A. Aziz, Wesam S. Shehab, Ahmed A. Al-Karmalawy, Ahmed F. EL-Farargy, Magda H. Abdellattif

**Affiliations:** 1 Department of Chemistry, Faculty of Science, Zagazig University, Zagazig 44519, Egypt; dr.wesamshehab@gmail.com (W.S.S.); aelfarargy@hotmail.com (A.F.E.-F.); 2 Department of Pharmaceutical Medicinal Chemistry, Faculty of Pharmacy, Horus University Egypt, New Damietta 34518, Egypt; akarmalawy@horus.edu.eg; 3 Department of Chemistry, College of Science, Taif University, P.O. Box 11099, Taif 21944, Saudi Arabia; m.hasan@tu.edu.sa

**Keywords:** indole chalcones, 2D-QSAR modeling, ABTS assay, antioxidant activity, ascorbic acid, docking studies

## Abstract

Novel candidates of 3-(4-(thiophen-2-yl)-pyridin/pyran/pyrimidin/pyrazol-2-yl)-1*H*-indole derivatives (**2**–**12**) were designed by pairing the pyridine/pyrane/pyrimidine/pyrazole heterocycles with indole and thiophene to investigate their potential activities as (2,2′-azinobis (3-ethylbenzothiazoline-6-sulfonic acid) inhibitors. The purpose of these derivatives’ modification is to create high-efficiency antioxidants, especially against ABTS, as a result of the efficiency of this set of key heterocycles in the inhibition of ROS. Herein, 2D QSAR modeling was performed to recommend the most promising members for further in vitro investigations. Furthermore, the pharmacological assay for antioxidant activity evaluation of the yielded indole-based heterocycles was tested against ABTS (2,2′-azinobis (3-ethylbenzothiazoline-6-sulfonic acid); by utilizing ascorbic acid as the standard. Candidate 10 showed higher antioxidant activity (IC_50_ = 28.23 μg/mL) than ascorbic acid itself which achieved (IC_50_ = 30.03 μg/mL). Moreover, molecular docking studies were performed for the newly designed and synthesized drug candidates to propose their mechanism of action as promising cytochrome *c* peroxidase inhibitors compared to ascorbic acid as a reference standard. Our findings could be promising in the medicinal chemistry scope for further optimization of the newly designed and synthesized compounds regarding the introduced structure-activity relationship study (SAR) in order to get a superior antioxidant lead compound in the near future.

## 1. Introduction

Reactive oxygen species (ROS) are a normal product of cellular metabolism in a human cell. Some of these radicals are required for regular cell functions, including neurological signal transmission [1]. Otherwise, the excess production of ROS threatens the human body in various forms and causes a variety of dangers [2]. All cellular macromolecules, including nucleic acids, proteins, carbohydrates, and lipids, can be damaged by excessive ROS and reactive nitrogen species (RNS). ROS can decay human cells by destroying cell membrane lipid, and by extension, change the cell permeability and cleavage of DNA [3]. This oxidative stress creates many risks in the human body and exposes it to various diseases such as Alzheimer’s [2], neurodegenerative diseases, inflammatory diseases, ischemia-reperfusion injury, diabetes, and aging [4].

During normal physiological conditions, most species are formed on a small scale and are scavenged by intracellular antioxidant systems such as small molecules; vitamins C and E, and superoxide dismutase (SOD) [4]. Nevertheless, excess levels of ROS require the intake of highly potent antioxidants to discourage damage to the body.

Ascorbic acid (vitamin C) is present in many fruits and vegetables such as oranges, guava, lemons, berries, broccoli, mango, and peppers, which is crucial in the human diet. Szent-Györgyi first isolated ascorbic as an “acidic carbohydrate” from adrenal glands, lemons, cabbages, and oranges in 1928, and in 1933, Norman Haworth illustrated the chemical structure of vitamin C [5]. In the late 1990s, its chemistry and biochemistry have been discovered [6]. The antioxidant activity of ascorbic acid is one of the most important functions in the body. It has a high ability to inhibit the ROS damages on body cells [7]. In addition, it has a remarkable ability to remove peroxides and convert them into water [8]. In vitro, ascorbate has a wide range of highly effective direct antioxidant effects [9].

Admittedly, heterocyclic moieties have vital biological activities, particularly that containing nitrogen, oxygen, and sulfur atoms [10,11]. Based on our belief in the importance of these candidates, in this paper, we prepared new series containing the indole nucleus and tested their effectiveness as antioxidants. Indolic compounds are powerful antioxidants that protect both proteins and lipids from oxidation processes. The research background for indole candidates proves their potency as antioxidants [12,13]. It is expected that indolic compounds exhibited these abilities and great effectiveness in inhibiting ROS, as it is the main nucleus in melatonin [14]. On the other hand, thiophene derivatives are a precious category of heterocyclic candidates with promising medicinal chemistry applications. They showed versatile physiological and biological roles such as antioxidant, anti-inflammatory [15], antihypertensive [16], anticancer [17], antimicrobial, antimalarial, anti-psychotic, antidepressant, anti-arrhythmic, kinases inhibiting, and antimycobacterial [18]. One of the promising cores that can trap ROS was pyrimidine [19]. Undisputed pyrimidine derivatives, as essential nucleobase, have achieved undeniable results as antioxidant agents against all ROS and RNS [20,21]. Moreover, pyrazole [22], pyrane [23], and pyridine [24,25] rings exhibited vital antioxidants properties in their derivatives. So, we target synthesized new candidates with pyrimidine, pyrazole, pyrane, and pyridine rings in the present work.

One of the best-known computational tools applied in medicinal chemistry is the quantitative structure–activity relationship (QSAR). QSAR can predict the potential of various chemicals and their biological activities by statistical calculations. It gave impressive, truthful, and realistic results if the basic data were available for measurement on its basis in advance [26]. Additionally, it proved their ability to discover compounds with different potencies and demonstrated results that were largely identical to experimental tests for the same compounds. Some examples of these agents include anticonvulsants [27], HIV-1 reverse transcriptase inhibitors [28], anticancer [29]. It is used in several crucial applications, such as determining the presence or absence of harmful features of synthesized drugs, instead of measuring it on animals and exposing many animals to danger [30].

Furthermore, we studied our compounds with molecular docking methodology, predicting the prevailing binding mode(s) of a ligand with a protein in the three-dimensional structure. Docking is an extremely useful strategy in optimizing the best candidates in the fastest and least expensive way by conducting virtual screening on large libraries of drugs, ranking the outcomes, and providing structural assumptions about how the ligands inhibit the target [31].

## 2. Rationale of the Work

**Figure 1:**
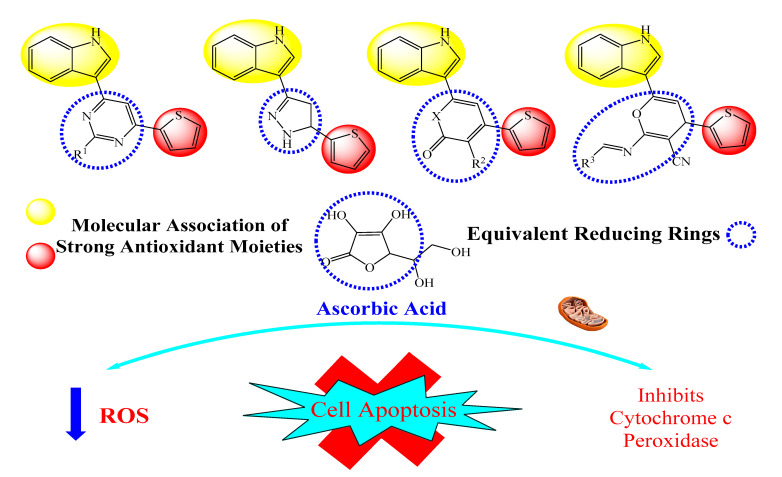
Rationale of the newly synthesized 1*H*-3-indolyl derivatives using molecular association.

Based on the aforementioned information, we designed and synthesized a new series of promising antioxidants by choosing different reducing moieties (pyrimidines, pyrazoles, pyridines, and pyrans) which are corresponding to the reducing ascorbic acid ring and incorporating each in between two potential antioxidant moieties (indole and thiophene rings, respectively) using the molecular association approach in Figure 1.

The molecular association was recommended to produce an additive or synergistic action. Moreover, it was approved to provide a broader spectrum of activity as well [32].

## 3. Results and Discussion

### 3.1. Chemistry

**Scheme 1:**
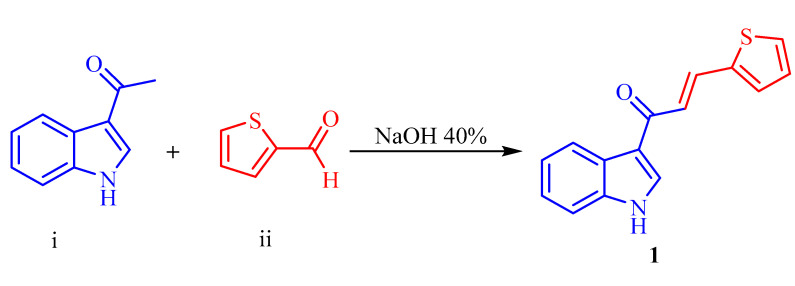
Synthesis of the starting candidate.

In our strategy to design and synthesize novel heterocyclic moieties as antioxidants, including indole nucleus, we synthesized a chalcone (**1**). The essential compound (**1**) that would be used in synthesizing the target indolyl derivatives (**2**–**12**); was conducted as shown in Scheme 1. 3-acetylindole (**i**) was prepared as reported previously through the Acylation of 1-(Pheny1sulfonyl)indole according to Friedel-Crafts method [33]. 1-(1*H*-indol-3-yl)-3-(thiophen-2-yl)prop-2-en-1-one (**1**) was developed by the condensation reaction of 3-acetyl indole (**i**) and thiophene-2-carbaldehyde (**ii**) in a basic medium (NaOH 40%). The chalcone structure **1** was elucidated with IR, ^13^C-NMR, ^1^H-NMR spectra, elemental analysis data, and mass spectroscopy.

The IR spectrum of **1** showed a definite peak at 1650 cm^−1^ due to the resulted *α,β*-unsaturated carbonyl. NH stretching of indole appears at 3400–3500 cm^−1^ as broadband. ^1^H-NMR spectra exhibited olefinic carbons of *α,β*-unsaturated carbonyl (C_11,12_) at 7.17 and 7.6 ppm with coupling constant *J* = 8.0 and 7.5 Hz, respectively. ^13^C-NMR spectra showed carbonyl carbon at 183.6 ppm and *α,β*-unsaturated carbons at 128.9 and 135 ppm.

**Scheme 2:**
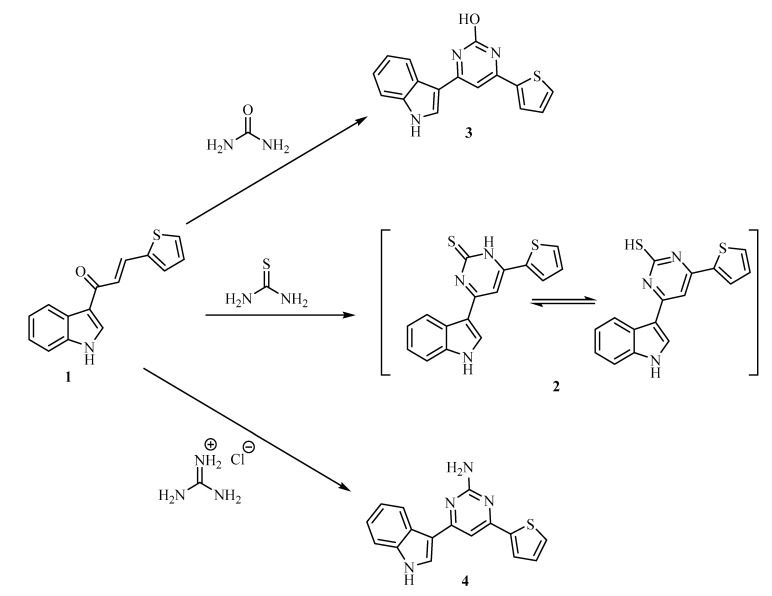
Synthesis of functional pyrimidine ring by hetero-cyclization of chalcone **1**.

In the present approach, we aimed to synthesize novel candidates over three preparing lines. Firstly, to discover a novel series of drugs containing a pyrimidine ring with indole moieties. We utilized highly functionalized reagents as thiourea, urea, and guanidine to give **2**, **3**, and **4,** respectively, as shown in Scheme 2. These compounds’ structures were confirmed with IR, ^1^H-NMR, ^13^C-NMR spectra and mass spectroscopy, and elemental analysis data. 4-(1*H*-indol-3-yl)-6-(thiophen-2-yl)pyrimidine-2-thiol (**2**) can be obtained by reacting thiourea with the start material under reflux for 13 h in the presence of sodium ethoxide as a base.

The IR spectrum of **2** illustrated a broad peak at 3350–3600 cm^−1^ due to 2NH groups stretching. The carbonyl of started chalcone disappeared, and the thione group that resulted from the formation of the pyrimidine ring via hetero-cyclization appeared at 860 and 825 cm^−1^. Moreover, the thiol group appeared at 2320 cm^−1^, which indicated that they are in tautomerism conformation. ^1^H-NMR of (**2**) showed a singlet peak for methine carbon (C_11_) at 8.65 ppm. While, NH, SH groups at 12.074 and 11.95 ppm, respectively. The structure of 4-(1*H*-indol-3-yl)-6-(thiophen-2-yl)pyrimidin-2-ol (**3**) was demonstrated by the IR spectrum. As OH and NH group depict stretching frequency in wide rang 3350–3560 cm^−1^ as a broadband. The presence of OH and NH groups was confirmed by ^1^H-NMR at values 10.76 and 12.08 ppm, respectively. Cyclization as pyrimidine ring illustrated the disappearance value of the ketonic-carbonyl group and the appearance of the carbinol (C_20_) at 162.4 ppm in ^13^C-NMR. 4-(1*H*-indol-3-yl)-6-(thiophen-2-yl)pyrimidin-2-ol (**3**) was afforded as yellow crystals by reacting the starting chalcone with urea using potassium hydroxide as a catalyst. On the other hand, 4-(1*H*-indol-3-yl)-6-(thiophen-2-yl)pyrimidin-2-amine (**4**) was synthesized by the reaction of the starting chalcone **1** with guanidine carbonate in dry ethanol and anhydrous sodium acetate. The IR spectrum estimated this compound’s structure by appearing as a broadband at 3400–3576 cm^−1^ due to NH and NH_2_ groups’ stretching frequency.

**Scheme 3:**
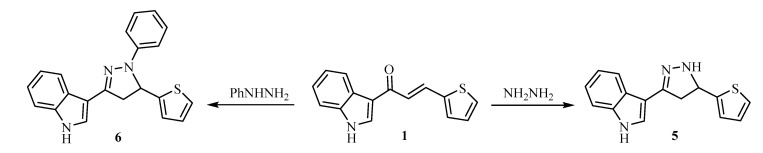
Synthesis of pyrazoles by cyclo-condensation reaction of chalcone **1** with hydrazines.

The second line in our schematic strategy is to synthesize indolylpyrazoles, as shown in Scheme 3. Cyclo-condensation of *α,β*-ethylenic ketone **1** with hydrazine in the presence of glacial acetic acid under refluxing for 7 h yielded yellow crystals of 3-(5-(thiophen-2-yl)-4,5-dihydro-1*H*-pyrazol-3-yl)-1*H*-indole (**5**). Under the same condition, 3-(1-phenyl-5-(thiophen-2-yl)-4,5-dihydro-1*H*-pyrazol-3-yl)-1*H*-indole (**6**) was synthesized by refluxing phenylhydrazine with the start chalone **1**. The chemical structures of compounds **5** and **6** were demonstrated with IR, ^1^H-NMR, ^13^C-NMR spectra, and mass spectroscopy, and elemental analysis data.

The structure of **5** was confirmed via IR spectrum through the stretching value of the carbonyl group and disappearance and methylene group (C_11_) appearance in ^1^H-NMR as a doublet peak at 3.48 ppm with a coupling constant *J* = 14.5 and 12.2 Hz. Additionally, the methine group (C_12_) appeared at 5.003 ppm with *J* = 7.0 Hz as a triplet peak. In the same strategy, the structure of **6** was elucidated. In ^1^H-NMR, the methylene carbon emerged at 3.32 ppm with *J* = 15.7 and 13.2 Hz as a doublet peak and methine carbon at 5.65 ppm and *J* = 6.5 Hz as a triblet peak.

**Scheme 4:**
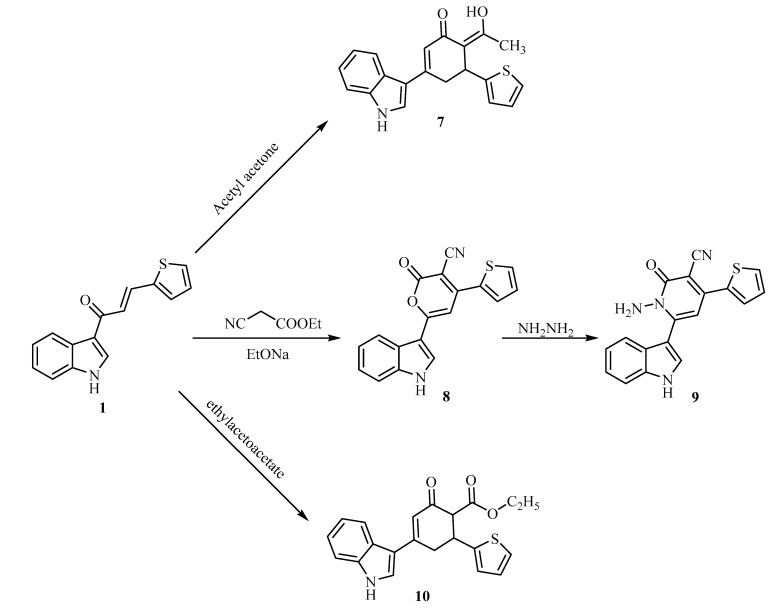
Synthesis A six-membered ring (pyran/cyclohexenone/pyridone) from the mother candidate using various reagents.

The third synthetic approach to synthesize the functional compounds (**7**–**10**) was depicted in Scheme 4. Firstly, cyclohexenes **7** and **10** were synthesized via cyclo-condensation of the chalcone **1** with different reagents in a sodium hydroxide (10%) basic medium as a catalyst. 6-(1-hydroxyethylidene)-3-(1*H*-indol-3-yl)-5-(thiophen-2-yl)cyclohex-2-en-1-one (**7**) was prepared by addition of acetylacetone to chalcone **1** in equilibrium quantities under refluxing for 5 h. Similarly, ethyl 4-(1*H*-indol-3-yl)-2-oxo-6-(thiophen-2-yl)cyclohex-3-ene-1-carboxylate (**10**) was synthesized under the same conditions but with using ethyl acetoacetate as a reagent. Whereas, the 2-pyranone ring 6-(1*H*-indol-3-yl)-2-oxo-4-(thiophen-2-yl)-2H-pyran-3-carbonitrile (**8**) was synthesized through the reaction of chalcone **1** with ethyl cyanoacetate in the presence of sodium ethoxide as a catalyst at room temperature. Treatment of compound **8** with hydrazine hydrate under refluxing for 6 h converted the pyranone ring to 2-pyridone to give 1-amino-6-(1*H*-indol-3-yl)-2-oxo-4-(thiophen-2-yl)-1,2-dihydropyridine-3-carbonitrile (**9**). The structures of compounds **7**, **8**, **9**, and **10** were elucidated by IR, ^1^H-NMR, ^13^C-NMR spectra, and mass spectroscopy, also elemental analysis (For further illustration, see experimental part). 

**Scheme 5:**
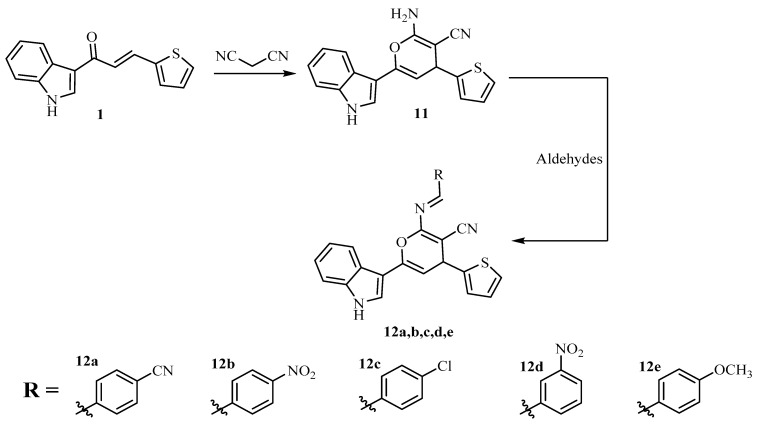
Formation of 2-amino-6-(1*H*-indol-3-yl)-4-(thiophen-2-yl)-4*H*-pyran-3-carbonitrile and its condensed derivatives.

Finally, the reaction of malononitrile with the start chalcone yielded 2-amino-6-(1*H*-indol-3-yl)-4-(thiophen-2-yl)-4*H*-pyran-3-carbonitrile (**11**) with a poly-functional pyrane ring, which can pave the way to synthesize a new series of novel compounds (**12a**–**e**) Scheme 5. A vicinal amino and cyano group can generate a wide range of various heterocyclic compounds [10,11]. In this regard, we envisioned synthesizing imine products by reacting the amino group of **11** with various aldehydes via different condensation reactions. Spectroscopic analysis (IR, ^1^H-NMR, ^13^C-NMR, and mass spectroscopy) revealed that all derivatives formed via condensation of **11** with various aldehydes are open and not heterocyclized with the cyano group. Compound **11** was demonstrated by exhibiting the main functional groups in IR spectra at 2214 cm^−1^ (CN), and 3465–3584 cm^−1^ (NH, NH_2_). Additionally, ^1^H-NMR spectra exhibited NH_2_ at δ 10.8 ppm, and (CH) methine groups at δ 4.51, 5.42 ppm. Furthermore, all carbons were observed in the ^13^C-NMR spectrum, as shown in the experimental part. In addition, all chemical structures of compounds **12a**–**e** were illustrated in the IR spectrum by the observed imine group at the range of 1654 cm^−1^, and the cyano group still presented at 2212 cm^−1^. The experimental part observed further spectroscopic analysis (^1^H-NMR, ^13^C-NMR, MS, and elemental analysis).

### 3.2. In Vitro Antioxidant Activity

In the current work, we selected the most promising candidates of our newly synthesized moieties depending on the 2D-QSAR results and evaluated them as antioxidants. The antioxidants properties of our selected candidates were screened by using ABTS (2,2′-azinobis(3-ethylbenzothiazoline-6-sulfonic acid) assay. The in vitro antioxidant assay (ABTS test) showed varied significant levels of free radical inhibition activity of indolyl derivatives **2**, **3**, **4**, **7**, **8**, **10**, **12b**, **12d**, **12e**, as shown in Table 1.

**Figure 2:**
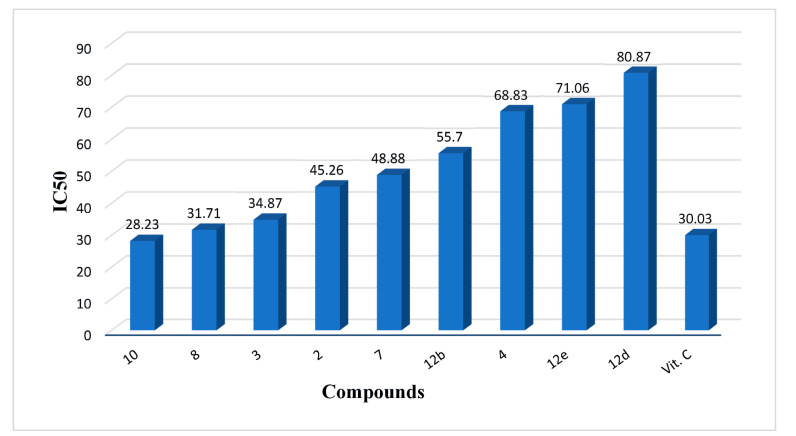
ABTS radical scavenging effect of (thiophen-2-yl)-1*H*-indole derivatives with standard ascorbic acid.

The recorded results revealed that our candidates might be arranged into three levels according to their activities. Firstly, ethyl-4-(1*H*-indol-3-yl)-2-oxo-6-(thiophen-2-yl)cyclohex-3-ene-1-carboxylate **10** showed the highest antioxidant activity (IC_50_ = 28.23 μg/mL) which was found to be superior to the antioxidant activity of *L*-ascorbic acid (IC_50_ = 30.23 μg/mL), as a reference antioxidant compound. While **8**, **3**, **2**, and **7** exhibited moderate antioxidant properties (IC_50_ = 31.71, 34.87, 45.26, 48.88 μg/mL, respectively). Finally, the rest of the tested compounds displayed modest activities compared to *L*-ascorbic acid (IC_50_ = 30.03 μg/mL), as shown in Figure 2.

Compound **10** achieved the most effective anti-ABTS·^+^ indoles among all the tested derivatives, in addition to its antioxidant property, which was found to be higher than that of ascorbic acid itself. This potential activity of this compound was proposed to be due to the presence of three oxygen atoms. One of them presents as an ethoxy group which enhanced the antioxidant activity. Furthermore, these oxygen atoms increased the solubility degree of the drug in aqueous media via the formation of excessive hydrogen bonds, which improves this candidate’s in vitro antioxidant properties.

**Figure 3:**
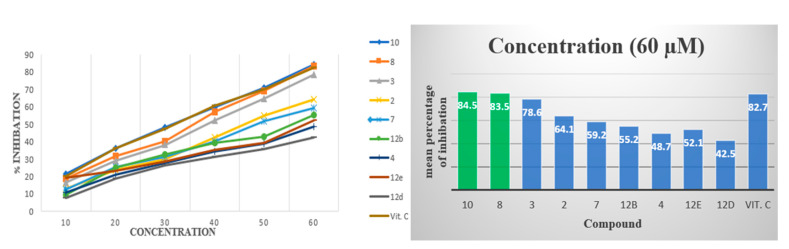
Graphical presentation of considerable antioxidant activity of thiophen-2-ylindole candidates (**2**, **3**, **4**, **7**, **8**, **10**, **12b**, **12d**, and **12e**).

On the other hand, compounds **4**, **12b**, **12d**, and **12e** exhibited antioxidant activities but were lower than ascorbic due to the excessive aromatic rings. More aromatic rings have electron-withdrawing effects that may inhibit the activity of compounds against the free radicals. Another consequence of these aromatic rings on the activity of these compounds is increasing their lipophilicity. The lower activity of these candidates made us infer that the excessive number of free functional groups containing oxygen and nitrogen atoms, besides decreasing the number of aromatic rings, might play a significant role in the redox reaction process. The percentage of inhibition of the tested compounds, via scavenging their activities against ABTS, revealed that compound **10** achieved a mean inhibition greater than ascorbic acid over all the most different concentrations in addition to compound **8,** which exhibited inhibition higher than ascorbic acid at a concentration (60 µm) as shown in Figure 3.

### 3.3. Structure-Activity Relationship Study

**Figure 4:**
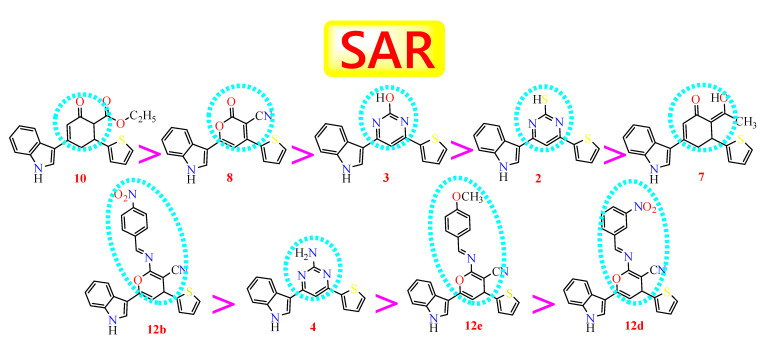
Structure-activity relationship study of the newly designed and synthesized derivatives tested biologically as antioxidants.

Regarding the structure-activity relationship study of the newly designed and synthesized candidates Figure 4, we can conclude the following interesting results:

(1) The introduction of simple six-membered rings in the linker region in between the indole and thiophene rings (**10**, **8**, **3**, **2**, and **7**) was found to be superior to its attachment to chalcone moieties (**12b**, **4**, **12e**, and **12d**).
(2) The introduction of hydrophilic functional groups in position 2 of the pyrimidine linker was observed to be in the following order (−OH > −SH > −NH_2_), corresponding to compounds **3**, **2**, and **4**, respectively.
(3) The chalcone linker formed by *p*-NO_2_ benzaldehyde derivative (**12b**) was better than the *m*-NO_2_ one (**12d**).
(4) The presence of additional oxygen functional groups such as carbonyl or hydroxyl, even methoxy groups in the linker six-membered ring increases the antioxidant properties of these moieties.

### 3.4. Computational Chemistry

#### 3.4.1. Docking Study

In our way to study the proposed mechanism of action for the newly designed and synthesized drug candidates as promising antioxidants compared to ascorbic acid as a reference standard, we performed the molecular docking study for the prepared database containing the previously mentioned new compounds besides the co-crystallized ascorbic acid extracted from cytochrome *c* peroxidase enzyme (PDB code: 2X08) [34]. It was found to be bound to the γ-heme edge of cytochrome *c* peroxidase through the formation of four H-bonds with Gly41, His181, Arg184, and Val45 amino acids. Molecular docking results of the two most promising compounds (**10** and **8**) compared to the docked ascorbic acid as a reference standard were depicted in Table 2 and Table 3.

Table 2 shows that compounds (**10** and **8**) got stabilized inside the binding pocket of cytochrome *c* peroxidase with very promising binding scores of −7.49 and −7.24 kcal/mol, respectively, compared to that of ascorbic acid (−4.60 kcal/mol). Moreover, ascorbic acid formed three H-bonds with Met172 and His175 at 3.21, 3.29, and 3.32 Å, respectively. On the other hand, compound (**10**) was stabilized inside the binding pocket of cytochrome *c* peroxidase by forming only one H-bond with Ser185 and one pi-pi interaction with His175 at 2.98 and 3.82, respectively, indicating a great binding affinity and an expecting intrinsic activity as well. However, compound (**8**) bound Arg184, Ser185, and His181 with three H-bonds at 2.89, 3.15, and 3.19 Å, respectively. Moreover, it formed a fourth H-pi interaction with His175 at 4.46 Å as represented in Table 2 and Table 3.

#### 3.4.2. QSAR Modeling

**Figure 5:**
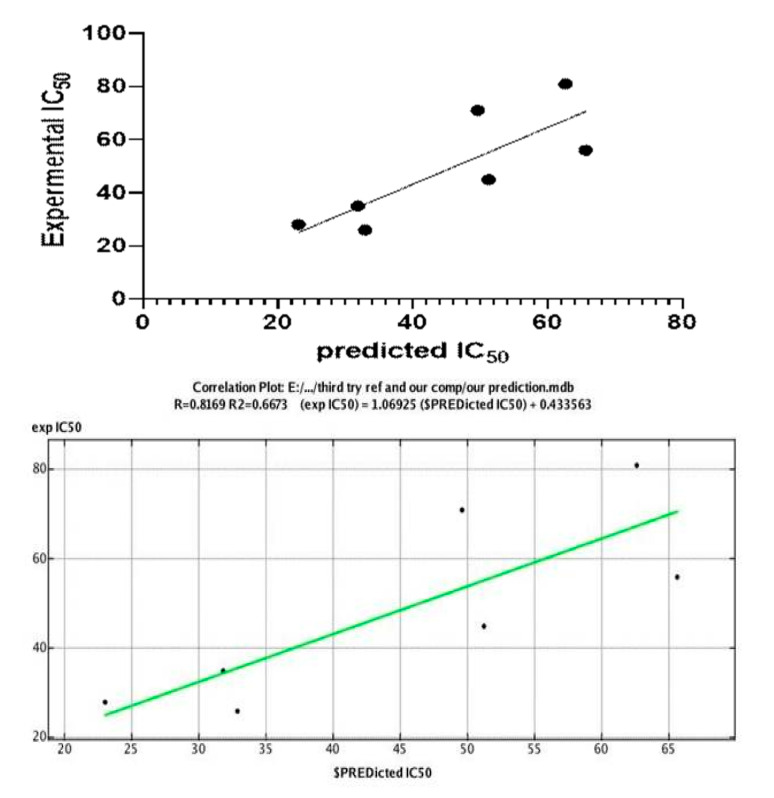
The correlation plot between predicted IC50 ($Pred.) values on the *x*-axis and IC50 values on the *y*-axis for compounds **2**, **10**, **3**, **12b**, **12d**, **12e**, and ascorbic acid. The plots give R^2^ values equal to 0.6673.

QSAR prediction is an in silico tool extensively, and it is applied to evaluate the structure-activity relationship of newly synthesized compounds. By using MOE software, a QSAR model of (1*H*-indol-3-yl) derivatives was developed [35,36]. Many 2D descriptors can be calculated by the empirical method (AM1-HF), such as AM1_Dipole, a descriptor for total energy (kcal/mol) (AM1_E). The heat of the formation descriptor, the energy of the lowest unoccupied molecular orbital descriptors, can be formulated and calculated using the empirical method (AM1-LUMO). The energy of the highest occupied molecular orbital descriptors can be formulated and calculated using the empirical method (AM1-HOMO), ionization potential(eV) (AM_IP), the number of double bonds (b_double), a descriptor of acidity at pH = 7 (h_pKa). The descriptor of basicity at pH = 7 (h_pKb), were calculated [37]. Hence, the partial least squares (PLS) method was used to obtain the QSAR model. We use the antioxidant result of 11 compounds Table 4 with 9 new synthesized compounds in this study and the reference ascorbic acid as the training set Table 5 [38]. The best model induced by the (PLS) method indicated a precise correlation with an r^2^ value of 0.09652. Figure 5 represented the correlation between the predicted and the experimental IC50 values. The prediction of biological activities of compounds from the test set examined the predictive efficiency of the model. The antioxidant IC_50_ are evaluated according to the QSAR model by the following equation:

(1) IC50 = 41.60348 − 0.04968 ∗ AM1_dipole + 0.00040 ∗ AM1_E + 0.14380 ∗ AM1_HF + 0.00425 AM1_HOMO − 0.00425 ∗ AM1_IP − 0.00298 ∗ AM1_LUMO − 0.00371 ∗ b_double 
+ 0.54160 ∗ + 0.04828 ∗ h_pKa + 0.17928 ∗ h_pKb

Additionally, ESTIMATED NORMALIZED LINEAR MODEL (SD = Standard Deviation) can be calculated by the following equation:

(2) IC50/SD(IC50) = 0.85934 − 0.00172 ∗ AM1_dipole/SD(AM1_dipole) + 0.14919 ∗ AM1_E/SD(AM1_E) + 0.37041 ∗ AM1_HF/SD(AM1_HF) + 0.00004 ∗ AM1_HOMO/SD(AM1_HOMO) − 0.00004 ∗ AM1_IP/SD(AM1_IP) − 0.00002 ∗ AM1_LUMO/SD(AM1_LUMO) − 0.00009 ∗ b_double/SD(b_double) + 0.24091 ∗ E/SD(E) + 0.00296 ∗ h_pKa/SD(h_pKa) + 0.00735 ∗ h_pKb/SD(h_pKb)

Table 6 shows a measure of the relative importance of descriptors and an estimate of compound similarity, which was detected by the relative importance of descriptors. The predicted IC50 values from 2D-QSAR calculations exhibited potency similar to the experimented values of IC50 of our candidates against ABTS assay Table 7.

## 4. Materials and Methods

### 4.1. Chemistry

All chemicals included 3-Acetylindole were provided from Sigma-Aldrich (St. Louis, MO, USA), and all solvents included annular ethanol delivered from El-NASR Co., Egypt. All the reactions were carried out with readily available reagents that were utilized without additional purification as received. Digital Electrothermal IA 9100 Series apparatus Cole-Parmer, Beacon Road, Stone, Staffordshire, UK) used to measure the melting points that they are uncorrected. Mass spectra were recorded in the Thermo scientific GCMS model (ISQLT) via the direct probe controller inlet part to a single quadrupole mass analyzer utilizing Thermo X-Cali bur software, at Al-Azhar University, (RCMB), Naser City, Cairo. IR spectra were carried out in the range from 4000 to 400 cm^−1^ on (Thermo-Fisher Scientific) FT-IR PLUS spectrometer (υ by cm^−1^) utilizing potassium bromide disks (KBr) at the microanalytical Laboratory, Faculty of Science, Cairo University, Egypt. C, H, and N analyses were carried out on a PerkinElmer CHN 2400. ^1^H and ^13^C-NMR spectra were carried out on a Bruker NMR spectrophotometer at 400 MHz in DMSO-*d_6_* using tetramethylsilane (TMS) as the internal reference standard, chemical shifts are expressed in δ which given in parts per million (ppm), and DMSO-*d_6_* was used as the solvent. All spectral analysis data are provided in the supplementary materials.

**1-(1*H*-indol-3-yl)-3-(thiophen-2-yl)prop-2-en-1-one (1)**

A few drops of 40% NaOH aqueous solution were added portion-wise to a mixture of 3-acetylindole (20 mmol, 3.2 g) and thiophene-2-carbaldehyde (20 mmol, 1.9 mL) in 50 mL ethanol. The reaction mixture was stirred for 1h at room temperature. Filtration gave a pale-yellow precipitate. A 4% aqueous HCl was utilized to wash the precipitate and then were crystallized from ethanol to give chalcone **1**. Yield: 85%. m.p.: 237–240 °C. IR (ν/cm^−1^, KBr): 3400–3500 cm^−1^ (NH, str), 3091 cm^−1^ (CH-Aryl), 2975 cm^−1^ (CH-Aliphatic), 1650 cm^−1^ (C=O). ^1^H-NMR (400 MHz, DMSO-*d*_6_): δ = 7.17 (*d*, *J* = 8.0 Hz, 1*H*, CO-CH=CH), 7.6 (*d*, *J* = 7.5 Hz, 1*H*, CO-CH=CH), 7.18–7.82 (*m*, 7*H*, ArH), 8.67 (*s*, 1*H*, C=CH-NH), and 12.1 (s, 1*H*, NH exchangeable by D_2_O). ^13^C-NMR (100MHz, DMSO-*d*_6_): δ = 128.9 ppm (CO-CH=CH), 135 ppm (CO-CH=CH), 183.6 ppm (CO), 129, 131, 132, 140 ppm (thiophene), 112.66, 117.94, 122.22, 123.58, 123.68, 126.32, 137.33 ppm (indolyl carbons). MS (*M/Z*): M^+•^ (253.20), Base-peak (57). Anal. Calcd. for C_15_H_11_NOS (253.32): C, 71.12; H, 4.38; N, 5.53; S, 12.66. Found: C, 71.31; H, 4.21; N, 5.65; S, 12.73.

**4-(1*H*-indol-3-yl)-6-(thiophen-2-yl)pyrimidine-2-thiol (2)**

A freshly prepared EtO^−^Na^+^ solution (by adding sodium metal (0.23 g, 10 mmol) in 40 mL of absolute ethanol) was added to a mixture of Chalcone **1** (10 mmol) and thiourea (10 mmol). The reaction mixture was refluxed for 13 h. After cooling, the mixture was neutralized with diluted hydrochloric acid in crushed ice, filtered, washed with ethanol, and dried. Crystallization from ethanol afforded the pyrimidine-2-thiol **2** as yellow crystals. Yield 66%. m.p.: 250–255 °C. IR (ν/cm^−1^, KBr): 3350–3600 cm^−1^ (NH, NH_2_, str), 3039 cm^−1^ (CH-Aryl), 2919 cm^−1^ (CH-Aliphatic), 2229 cm^−1^ (CN), 2320 cm^−1^ (thiol), 1588 cm^−1^ (C=C), 860, 825 cm^−1^ (thione group). ^1^H-NMR (400 MHz, DMSO-*d*_6_): δ = 8.65 (*s*, 1*H*, N-C=CH), 7.46–7.51 (*m*, ArH, thiophene), 7.58–7.81 (*m*, 7*H*, ArH), 12.074 (s, 1*H*, SH), and 11.95 (s, 1*H*, NHexchangeable by D_2_O). ^13^C-NMR (100MHz, DMSO-*d*_6_): δ = 181.4 ppm (C-SH), 127–142 ppm (thiophene), 111–136 ppm (indolyl carbons). MS (*M/Z*): M^+•^ (309.00), Base-peak (253). Anal. Calcd. for C_16_H_11_N_3_S_2_ (309.41): C, 62.11; H, 3.58; N, 13.58; S, 20.72. Found: C, 62.26; H, 3.39; N, 13.46; S, 20.83.

**4-(1*H*-indol-3-yl)-6-(thiophen-2-yl)pyrimidin-2-ol (3)**

A potassium hydroxide (10 mmol) was added to a mixture of Chalcone **1** (10 mmol) and urea (10 mmol). The reaction mixture was refluxed for 7 h. After cooling, the mixture was neutralized with diluted hydrochloric acid in crushed ice, filtered, washed with ethanol, and dried. Crystallization from ethanol afforded the pyrimidine-2-ol **3** as yellow crystals. Yield 82%. m.p.: 266–270°C. IR (ν/cm^−1^, KBr): 3350–3560 cm^−1^ (NH, OH, str), 3008 cm^−1^ (Aromatic-CH), 2975 cm^−1^ (CH-Aliphatic). ^1^H-NMR (400 MHz, DMSO-*d*_6_): δ = 7.18–7.51 (*m*, ArH, thiophene), 7.58–8.30 (*m*, 7*H*, ArH), 10.76 (s, 1*H*, OH), and 12.08 (s, 1*H*, NH exchangeable by D_2_O). ^13^C-NMR (100MHz, DMSO-*d*_6_): δ = 162.4 ppm (C-OH), 130–141 ppm (thiophene), 115–132 ppm (indolyl carbons). MS (*M/Z*): M^+•^ (294.15), Base-peak (253). Anal. Calcd. for C_16_H_11_N_3_OS (293.34): C, 65.51; H, 3.78; N, 14.32; S, 10.93. Found: C, 65.32; H, 3.91; N, 14.19; S, 11.09.

**4-(1*H*-indol-3-yl)-6-(thiophen-2-yl)pyrimidin-2-amine (4)**

By mixing (1 mmol) of chalcone **1** with guanidine carbonate (10 mmol) in dry ethanol, then (10 mmol) of anhydrous sodium acetate was added. The reaction mixture was refluxed on a water bath for 4 h. After cooling, the mixture was neutralized with diluted hydrochloric acid in crushed ice, filtered, then washed with ethanol, and finally dried. After that, afforded precipitate was crystallized from ethanol to give the pyrimidine-2-amine **4** as pale-yellow crystals. Yield 75%. m.p.: 270–273 °C. IR (ν/cm^−1^, KBr): 3400–3576 cm^−1^ (NH, NH_2_, str), 3148 cm^−1^ (NH, str), 3039 cm^−1^ (CH-Aryl), 2918 cm^−1^ (CH-Aliphatic). ^1^H-NMR (400 MHz, DMSO-*d*_6_): δ = 8.64 (*s*, 1*H*, N-C=CH), 7.14–7.81 (*m*, ArH, thiophene), 7.48–8.57 (*m*, 7*H*, ArH), 12.04 (s, 1*H*, NH exchangeable by D_2_O), and 7.08 (s, 2*H*, NH_2_ exchangeable by D_2_O). ^13^C-NMR (100MHz, DMSO-*d*_6_): δ = 111.2, 114.3, 123.5, 126.02, 128.2, 128.9, 132.4, 134.7, 136.5, 138.4, 140.1, 142.3, 160.4, and 163.5 ppm. MS (*M/Z*): M^+•^ (292.56), Base-peak (253). Anal. Calcd. for C_16_H_12_N_4_S (292.36): C, 65.73; H, 4.14; N, 19.16; S, 10.97. Found: C, 65.57; H, 4.29; N, 19.31; S, 10.79. 

**3-(5-(thiophen-2-yl)-4,5-dihydro-1*H*-pyrazol-3-yl)-1*H*-indole (5)**

To a solution of the started chalcone **1** (10 mmol) in 30 mL absolute ethanol, hydrazine hydrate (99%, 20 mmol), and a few drops of glacial acetic acid were added, followed by refluxing the reaction mixture for 7 h. After cooling, the mixture was poured onto ice water. The crude product was filtered off and crystallized from ethanol to give pyrazolo indole **5** as yellow crystals. Yield 60%. m.p.: 210–215 °C. IR (ν/cm^−1^, KBr): 3465–3534 cm^−1^ (2NH, str), 3039 cm^−1^ (CH-Aryl), 2972 cm^−1^ (CH-Aliphatic). ^1^H-NMR (400 MHz, DMSO-*d*_6_): δ = 3.48 (*dd*, *J* = 14.5 & 12.2 Hz, 2*H*, methylene), 5.003 (t, *J* = 7.0 Hz, CH, methine), 6.96–7.1 (*m*, ArH, thiophene), 7.18–7.59 (*m*, 7*H*, ArH indole), 8.12 (s, 1*H*, NH hydrazide, exchangeable by D_2_O), and 11.39 (s, 1*H*, NH exchangeable by D_2_O). ^13^C-NMR (100MHz, DMSO-*d*_6_): δ = 44.2, 53.7, 112.2, 116.20, 122.5, 125.46, 127.5, 128.9, 133.4, 134.8, 136.05, 138.4, 142.1, and 145.3 ppm. MS (*M/Z*): M^+•^ (267.15), Base-peak (253). Anal. Calcd. for C_15_H_13_N_3_S (267.35): C, 67.39; H, 4.90; N, 15.72; S, 11.99. Found: C, 67.21; H, 5.01; N, 15.65; S, 12.08.

**3-(1-phenyl-5-(thiophen-2-yl)-4,5-dihydro-1*H*-pyrazol-3-yl)-1*H*-indole (6)**

To a solution of the started chalcone **1** (10 mmol) in 30 mL glacial acetic acid, phenylhydrazine (40 mmol) was added, then refluxing the reaction mixture for 7 h. After that, the reaction mixture was cooled then poured onto ice water. After filtration, the product was washed with water till neutralization, then recrystallized from ethanol to give pyrazolo indole **6** as yellow crystals. Yield 60%. m.p.: 210–215 °C. IR (ν/cm^−1^, KBr): 3403 cm^−1^ (NH, str), 3038 cm^−1^ (CH-Aryl), 2973 cm^−1^ (CH-Aliphatic). ^1^H-NMR (400 MHz, DMSO-*d*_6_): δ = 3.32 (*dd*, *J* = 15.7 & 13.2 Hz, 2*H*, methylene), 5.65 (t, *J* = 6.5 Hz, C*H*, methine), 6.68–7.47 (*m*, ArH, thiophene), 7.51–8.34 (*m*, 7*H*, ArH indole), and 12.08 (s, 1*H*, NH exchangeable by D_2_O). ^13^C-NMR (100MHz, DMSO-*d*_6_): δ = 42.2, 58.7, 112.4, 113.7, 116.7, 119.2, 121.4, 122.2, 124.46, 126.03, 127.5, 128.9, 133.4, 134.8, 136.05, 138.4, 141.1, and 152.3 ppm. MS (*M/Z*): M^+•^ (343.75), Base-peak (253). Anal. Calcd. for C_21_H_17_N_3_S (343.45): C, 73.44; H, 4.99; N, 12.24; S, 9.33. Found: C, 73.31; H, 5.08; N, 12.34; S, 9.25.

**6-(1-hydroxyethylidene)-3-(1*H*-indol-3-yl)-5-(thiophen-2-yl)cyclohex-2-en-1-one (7)**

A mixture of chalcone **1** (8 mmol) and acetylacetone (8 mmol) in ethanol (20 mL) in the presence of 10% NaOH (1 mL) was refluxed for 5 h. After cooling, the product was filtered and recrystallized from ethanol to yield the yellow crystals of **7**. Yield 80%. m.p.: 240–242 °C. IR (ν/cm^−1^, KBr): 3423 cm^−1^ (NH, str), 3074 cm^−1^ (Aromatic-CH), 2974 cm^−1^ (CH-Aliphatic). ^1^H-NMR (400 MHz, DMSO-*d*_6_): δ = 2.4 (*s*, 3*H*, methyl, =C(OH)CH_3_), 2.49;2.51 (*dd*, *J* = 15.3 &15.8 Hz, 2*H*, methylene), 3.1 (*t*, *J* = 6.6 Hz, C*H*, methine), 6.9–7.46 (*m*, ArH, thiophene), 7.1–8.3 (*m*, 7*H*, ArH indole), and 10.7 (s, 1*H*, NH exchangeable by D_2_O). ^13^C-NMR (100MHz, DMSO-*d*_6_): δ = 45.1, 47.6, 52.4, 57.6, 112.4, 113.7, 115.7, 120.2, 121.1, 122.4, 123.46, 125.03, 128.5, 129.9, 131.4, 135.8, 137.05, 138.4, 141.1, 158.4, and 161.3 ppm. MS (*M/Z*): M^+•^ (336.10), Base-peak (253). Anal. Calcd. for C_20_H_17_NO_2_S (335.42): C, 71.62; H, 5.11; N, 4.18; S, 9.56. Found: C, 71.41; H, 5.38; N, 4.04; S, 9.71.

**6-(1*H*-indol-3-yl)-2-oxo-4-(thiophen-2-yl)-2*H*-pyran-3-carbonitrile (8)**

A mixture of chalcone **1** (4 mmol, 1 g) and ethyl cyanoacetate (4 mmol, 0.5 g) in 50 mL ethanol was stirred at room temperature. A freshly prepared EtO^−^Na^+^ solution (By adding sodium metal (0.23 g, 10 mmol) in 40 mL of absolute ethanol) was added to this mixture. The reaction mixture was refluxed for 7 h. After cooling, the formed solid was collected by filtration, then washed with water, dried, and finally recrystallized from ethanol to create compound **8** as greenish-yellow crystals. Yield: 70%. m.p.: 288–290 °C. IR (ν/cm^−1^, KBr): 3400–3500 cm^−1^ (NH, str), 3039 cm^−1^ (CH-Aryl), 2920 cm^−1^ (CH-Aliphatic), 2283 cm^−1^ (CN), 1724 cm^−1^ (C=O for 2-pyrone cyclization), 1552 cm^−1^ (C=C). ^1^H-NMR (400 MHz, DMSO-*d*_6_): δ = 6.95 (*s*, 1*H*, O-C=CH), 8.32–8.65 (*m*, ArH, thiophene), 7.20–7.83 (*m*, 7*H*, ArH), and 12.03 (s, 1*H*, NH exchangeable by D_2_O). ^13^C-NMR (100MHz, DMSO-*d*_6_): δ = 115.8 ppm (CN), 127–130 ppm (thiophene), 110–130 ppm (indolyl carbons). MS (*M/Z*): M^+•^ (318.30), Base-peak (57). Anal. Calcd. for C_18_H_10_N_2_O_2_S (318.35): C, 67.91; H, 3.17; N, 8.80; S, 10.07. Found: C, 68.14; H, 2.99; N, 8.91; S, 9.95.

**1-amino-6-(1*H*-indol-3-yl)-2-oxo-4-(thiophen-2-yl)-1,2-dihydropyridine-3-carbonitrile (9)**

Hydrazine hydrate (2 mmol) was added to the pyranone **8** (2 mmol) in 30 mL of ethanol. The mixture was heated under reflux for 6 h. after cooling, the formed solid product was filtered off, dried, and then recrystallized from ethanol to give compounds **9** as a yellow powder. Yield: 75%. m.p.: 298–300 °C. IR (ν/cm^−1^, KBr): 3350–3600 cm^−1^ (NH, NH_2_, str), 3039 cm^−1^ (CH-Aryl), 2975 cm^−1^ (CH-Aliphatic), 2229 cm^−1^ (CN), 1640 cm^−1^ (C=O for cyclic amide), 1588 cm^−1^ (C=C). ^1^H-NMR (400 MHz, DMSO-*d*_6_): δ = 6.54 (*s*, 1*H*, N-C=CH), 8.32–9.6 (*m*, ArH, thiophene), 7.13–7.91 (*m*, 7*H*, ArH), 11.54 (s, 1*H*, NH exchangeable by D_2_O), and 6.23 (s, 2*H*, NH_2_ exchangeable by D_2_O). ^13^C-NMR (100MHz, DMSO-*d*_6_): δ = 116.4 ppm (CN), 164.21 (CO), 129–132 ppm (thiophene), 111–128 ppm (indolyl carbons). MS (*M/Z*): M^+•^ (333.05), Base-peak (57). Anal. Calcd. for C_18_H_12_N_4_OS (332.38): C, 65.05; H, 3.64; N, 16.86; S, 9.65. Found: C, 64.91; H, 3.76; N, 16.95; S, 9.51.

**Ethyl 4-(1*H*-indol-3-yl)-2-oxo-6-(thiophen-2-yl)cyclohex-3-ene-1-carboxylate (10)**

A mixture of chalcone **1** (8 mmol), and ethylacetoacetate (8 mmol) in ethanol (20 mL) in the presence of 10% NaOH (1 mL) was refluxed for 5 h. After cooling, the product was filtered and recrystallized from ethanol to yield the yellow crystals of **10**. Yield 85%. m.p.: 270–272 °C. IR (ν/cm^−1^, KBr): 3461 cm^−1^ (NH, str), 3005 cm^−1^ (CH-Aryl), 2974 cm^−1^ (CH-Aliphatic), 1725 cm^−1^ (C=O on cyclohexanone), 1735 cm^−1^ (C=O in ester form). ^1^H-NMR (400 MHz, DMSO-*d*_6_): δ = 1.10 (*t*, *J* = 7.4 Hz, 3*H*, CH_3_-CH_2_-O), 2.50 (*dd*, *J* = 18.4 &16.7 Hz, 2*H*, methylene), 3.08 (*d*, *J* = 4.9 Hz, 1*H*, CO-CH-CO), 3.43 (*q*, *J* = 7.8 Hz, 2*H*, O-CH_2_-CH_3_), 4.78 (t, *J* = 7.1 Hz, 1*H*, CH, methine), 7.16–7.46 (*m*, ArH, thiophene), 7.50–7.81 (*m*, 7*H*, ArH indole), and 10.72 (s, 1*H*, NH exchangeable by D_2_O). ^13^C-NMR (100MHz, DMSO-*d*_6_): δ = 21.2, 36.4, 43.2, 58.7, 65.7, 67.4, 112.4, 113.7, 118.6, 119.2, 121.4, 122.2, 124.46, 126.03, 127.5, 128.9, 133.4, 134.8, 138.4, 173.1, and 184.3 ppm. MS (*M/Z*): M^+•^ (364.95), Base-peak (57). Anal. Calcd. for C_21_H_19_NO_3_S (365.45): C, 69.02; H, 5.24; N, 3.83; S, 8.77. Found: C, 69.17; H, 5.09; N, 3.91; S, 8.59.

**2-amino-6-(1*H*-indol-3-yl)-4-(thiophen-2-yl)-4*H*-pyran-3-carbonitrile (11)**

A mixture of chalcone **1** (10 mmol) with malononitrile (10 mmol) and ammonium acetate (5 mmol) in ethanol 30 mL was refluxed for 6 h. The reaction mixture was cooled and poured into ice-cold acidified water. After neutralization, the crude precipitate was filtrated, then dried and recrystallized from ethanol to yield reddish-yellow crystals **11**. Yield 85%. m.p.: 245–250 °C. IR (ν/cm^−1^, KBr): 3465–3584 cm^−1^ (NH, NH_2_, str), 3003 cm^−1^ (CH-Aryl), 2971 cm^−1^ (CH-Aliphatic), 2214 cm^−1^ (CN). ^1^H-NMR (400 MHz, DMSO-*d*_6_): δ = 4.51 (*d*, *J* = 9.8 Hz, 1*H*, methine, CH-C(CN)=C), 5.42 (*d*, *J* = 10.2 Hz, 1*H*, CH=C-O), 7.17–7.27 (*m*, ArH, thiophene), 7.49–7.79 (*m*, 7*H*, ArH), 10.8 (s, 2*H*, NH_2_ exchangeable by D_2_O), and 12.10 (s, 1*H*, NH exchangeable by D_2_O). ^13^C-NMR (100MHz, DMSO-*d*_6_): δ = 30.67, 112.6, 117.9 (CN), 122.25, 122.33, 123.7, 126.35, 128.9, 129.35, 131.66, 132.9, 135.0, 137.35, 140.69, 183.63 (O-C-NH2) ppm. MS (*M/Z*): M^+•^ (319.55), Base-peak (57). Anal. Calcd. for C_18_H_13_N_3_OS (319.38): C, 67.69; H, 4.10; N, 13.16; S, 10.04. Found: C, 67.81; H, 3.99; N, 13.01; S, 10.12.

**General synthesis of 6-(1*H*-indol-3-yl)-4-(thiophen-2-yl)-4*H*-pyran-3-carbonitrile derivatives (12a–e)**

An appropriate aromatic aldehyde (10 mmol) was added to compound **11** (10 mmol) with few drops of piperidine (0.5 mL) in ethanol (20 mL). The reaction mixture was heated under reflux for 4 h, cooled and poured onto cold water, and filtered. The obtained solid was recrystallized from dioxane to give **12a-e.**

**2-((4-cyanobenzylidene)amino)-6-(1*H*-indol-3-yl)-4-(thiophen-2-yl)-4*H*-pyran-3- carbonitrile (12a)**

Yield 65%. m.p.: 285–290 °C. IR (ν/cm^−1^, KBr): 3392–3437 cm^−1^ (NH, str), 3003 cm^−1^ (CH-Aryl), 2929 cm^−1^ (CH-Aliphatic), 2212 cm^−1^ (CN), 1654 cm^−1^ (C=N). ^1^H-NMR (400 MHz, DMSO-*d*_6_): δ = 2.5 (*d*, *J* = 11.1 Hz, 1*H*, methine, CH-C(CN)=C), 3.35 (*d*, *J* = 10.9 Hz, 1*H*, CH=C-O), 7.2–8.16 (*m*, ArH), 11.95 (s, 1*H*, NH exchangeable by D_2_O). ^13^C-NMR (100MHz, DMSO-*d*_6_): δ = 30.67, 112.6, 117.9, 118.2 (CN), 122.25, 122.33, 123.7, 126.35, 128.9, 129.35, 131.66, 132.9, 135.0, 137.35, 140.69, 183.63 (O-C-NH_2_) ppm. Anal. Calcd. for C_26_H_16_N_4_OS (432.50): C, 72.20; H, 3.73; N, 12.95; S, 7.41. Found: C, 72.39; H, 3.61; N, 13.01; S, 7.29.

**6-(1*H*-indol-3-yl)-2-((4-nitrobenzylidene)amino)-4-(thiophen-2-yl)-4*H*-pyran-3-carbonitrile (12b)**

Yield 65%. m.p.: 285–290 °C. IR (ν/cm^−1^, KBr): 3368–3586 cm^−1^ (NH, str), 2920 cm^−1^ (CH-Aliphatic), 2201 cm^−1^ (CN), 1610 cm^−1^ (C=N). ^1^H-NMR (400 MHz, DMSO-*d*_6_): δ = 2.5 (*d*, *J* = 8.5 Hz, 1*H*, methine, CH-C(CN)=C), 3.34 (*d*, *J* = 8.7 Hz, 1*H*, CH=C-O), 7.1–8.52 (*m*, ArH), 11.75 (s, 1*H*, NH exchangeable by D_2_O). ^13^C-NMR (100MHz, DMSO-*d*_6_): δ = 42.51, 115.6 (CN), 121.25, 122.53, 125.7, 127.35, 128.56, 129.87, 130.66, 132.9, 134.0, 137.53, 150.69, 186.63 (O-C-NH_2_) ppm. MS (*M/Z*): M^+•^ (451.55), Base-peak (311). Anal. Calcd. for C_25_H_16_N_4_O_3_S (452.49): C, 66.36; H, 3.56; N, 12.38; S, 7.09. Found: C, 66.19; H, 3.71; N, 12.22; S, 7.21.

**2-((4-chlorobenzylidene)amino)-6-(1*H*-indol-3-yl)-4-(thiophen-2-yl)-4*H*-pyran-3-carbonitrile (12c)**

Yield 65%. m.p.: 285–290 °C. IR (ν/cm^−1^, KBr): 3395–3646 cm^−1^ (NH, str), 3050 cm^−1^ ( Aromatic-CH), 2921 cm^−1^ (CH-Aliphatic), 2201 cm^−1^ (CN), 1593 cm^−1^ (C=N). ^1^H-NMR (400 MHz, DMSO-*d*_6_): δ = 4.47 (*d*, *J* = 8.8 Hz, 1*H*, methine, CH-C(CN)=C), 5.04 (*d*, *J* = 9.0 Hz, 1*H*, CH=C-O), 7.35–7.93 (*m*, ArH), 10.76 (s, 1*H*, NH exchangeable by D_2_O). ^13^C-NMR (100MHz, DMSO-*d*_6_): δ = 30.67, 118.4 (CN), 120.25, 121.36, 123.7, 125.52, 127.9, 128.35, 131.53, 134.9, 135.25, 136.35, 146.69, 165.63 (O-C-NH_2_) ppm. Anal. Calcd. for C_25_H_16_ClN_3_OS (441.93): C, 67.95; H, 3.65; Cl, 8.02; N, 9.51; S, 7.25. Found: C, 67.82; H, 3.79; Cl, 8.21; N, 9.37; S, 7.19. 

**6-(1*H*-indol-3-yl)-2-((3-nitrobenzylidene)amino)-4-(thiophen-2-yl)-4*H*-pyran-3-carbonitrile (12d)**

Yield 65%. m.p.: 285–290 °C. IR (ν/cm^−1^, KBr): 3390–3674 cm^−1^ (NH, str), 3010 cm^−1^ (CH-Aryl), 2922 cm^−1^ (CH-Aliphatic), 2205 cm^−1^ (CN), 1609 cm^−1^ (C=N). ^1^H-NMR (400 MHz, DMSO-*d*_6_): δ = 2.9 (*d*, *J* = 9.2 Hz, 1*H*, methine, CH-C(CN)=C), 3.7 (*d*, *J* = 9.4 Hz, 1*H*, CH=C-O), 7.15–8.52 (*m*, ArH), 10.93 (s, 1*H*, NH exchangeable by D_2_O). ^13^C-NMR (100MHz, DMSO-*d*_6_): δ = 37.67, 116.6 (CN), 123.56, 123.88, 124.15, 124.36, 126.35, 129.10, 129.35, 131.66, 132.53, 136.0, 137.55, 145.69, 180.63 (O-C-NH_2_) ppm. MS (*M/Z*): M^+•^ (452.15), Base-peak (311). Anal. Calcd. for C_25_H_16_N_4_O_3_S (452.49): C, 66.36; H, 3.56; N, 12.38; S, 7.09. Found: C, 66.41; H, 3.43; N, 12.19; S, 7.21.

**6-(1*H*-indol-3-yl)-2-((4-methoxybenzylidene)amino)-4-(thiophen-2-yl)-4*H*-pyran-3-carbonitrile (12e)**

Yield 65%. m.p.: 285–290 °C. IR (ν/cm^−1^, KBr): 3370–3693 cm^−1^ (NH, str), 3102 cm^−1^ ( Aromatic-CH), 2924 cm^−1^ (CH-Aliphatic), 2206 cm^−1^ (CN), 1595 cm^−1^ (C=N).^1^H-NMR (400 MHz, DMSO-*d*_6_): δ = 3.6 (*d*, *J* = 9.6 Hz, 1*H*, methine, CH-C(CN)=C), 3.88 (*d*, *J* = 10.0 Hz, 1*H*, CH=C-O), 6.99–8.68 (*m*, ArH), 11.87 (s, 1*H*, NH exchangeable by D_2_O). ^13^C-NMR (100MHz, DMSO-*d*_6_): δ = 47.67, 116.5 (CN), 122.55, 122.78, 123.56, 126.44, 128.10, 129.10, 131.23, 132.41, 135.53, 137.45, 148.69, 167.63 (O-C-NH_2_) ppm. MS (*M/Z*): M^+•^ (436.10), Base-peak (311). Anal. Calcd. for C_26_H_19_N_3_O_2_S (437.52): C, 71.38; H, 4.38; N, 9.60; S, 7.33. Found: C, 71.51; H, 4.21; N, 9.73; S, 7.18.

### 4.2. Antioxidant Activity

All chemicals, including L-ascorbic acid, were provided from Sigma-Aldrich (St. Louis, MO, USA), and all solvents, including annular ethanol, delivered from El-NASR Co., Egypt. This assay follows the methodology of Re et al. [39]. ABTS method is a typical assay in evaluating the potency of antioxidant activities of many pure organic candidates. ABTS is an abbreviation of the chemical compound 2,2′-Azinobis (3-ethylbenzothiazoline-6-sulfonic acid). The chemical structure of this compound is very stable in free radical cation form (ABTS^·+^). ABTS^·+^ can react with any compound that generates a hydrogen atom (H-Donor) or an electron such as phenols and thiols, where ABTS·^+^ reacts as H or an electron acceptor as shown in Figure S1.

This transformation of hydrogen or electron converts ABTS^·+^ solution from a dark green one to a colorless solution. Equal amounts of ABTS^·+^ and potassium persulphate K_2_S_2_O_8_ (7 mM and 3.5 mM, respectively) were added from their stock solutions to make the ABTS^·+^ standard solution. The yielded mixture was left to stand at room temperature in the darkness for 13–15 h overnight. The ABTS^·+^ stock solution is appropriate for use after the completion of the reaction. The evidence of finishing the reaction is the stability of the spectrophotometric absorbance of the ABTS^·+^ solution at a wavelength of 735 nm. ABTS^·+^ stock solution may be stored at room temperature in the darkness for about 2–3 days and will still be valid over this period to use. The present assay was prepared the ABTS^·+^ working solution from stock one by dilution in annular ethanol to get an absorbance A_blank_ of 0.7 ± 0.02 at a wavelength of 735 nm. In an incubator, the produced solution was controlled at a temperature of 30 °C and was equilibrated. In this assay, A_blank_ was adapted to be accurately 0.7 at time = 0, i.e., before determining the absorbance for all the targeted compounds. In order to measure the scavenging activities of the targeted compounds against the free radicals, 1.5 mL of dark blue ABTS^·+^ working solution was mixed with 10 µL of the compounds solutions (**2**, **3**, **4**, **7**, **8**, **10**, **12b**, **12d**, **12e**). These steps are repeated using various concentrations of the tested compounds solutions over a range of 10–300 µM. These various concentrations are achieved by dilution using distilled water and annular ethanol or both, based on their solubility degree. After adding ABTS·+ solution with different tested candidates, the change in absorbance value was determined over various rang of time at 0, 0.5, 1, and 5 min in order to manage to achieve the steady-state value of absorbance. In our present assay, the steady-state value was achieved after 15 min. By generalization, A_test_, absorbance value for each tested candidate, was reported after 15 min of addition ABTS^·+^ solution tested candidate solution. The mean of the values was recorded. Each concentration for each tested sample at a specific time was recorded separately for all compounds, then every three separate measurements were determined, and the mean was taken. The antioxidant activity of each compound against ABTS^·+^ was calculated from the percent reduction in absorbance values, according to the following equation:

ABTS^+^ radical cation scavenging activity of test compound (%) = 100(A_blank_ − A_test_)/A_blank_. (3)

where A_test_ or A_15_ = The absorbance value for each tested candidate after 15 min of addition ABTS^·+^ solution. A_blank_ or A_0_ = The absorbance value of ABTS^·+^ itself before adding the tested candidates.

i.e., Time = 0. (A_blank_ was adjusted to be 0.70)

In this assay, after 15 min of reaction, the IC50 (inhibitory concentration 50 percent) of each of the test compounds was determined and calculated by Unico spectrophotometer 1200 USA and compared to that of L-ascorbic acid as the reference and standard.

### 4.3. Docking Study

A molecular docking study of the sixteen newly designed and synthesized indolic compounds was performed using the MOE 2019.0102 program [40]. The newly synthesized compounds were drawn using ChemDraw, imported into the MOE program window, converted for their 3D forms, adjusted for the partial charges, and energy minimized as described earlier [41,42]. The database was built containing the newly synthesized candidates (**1**–**12a**–**e**) together with the co-crystallized ascorbic acid as a reference standard. A general docking process was performed using the site of the co-crystallized ascorbic acid inside cytochrome *c* peroxidase as the docking site. Furthermore, all the other docking parameters were adjusted as previously discussed in detail [43,44]. Moreover, it is worth mentioning that a program validation process was performed at first before applying the docking process by redocking the co-crystallized ascorbic acid at its binding pocket of the cytochrome *c* peroxidase enzyme. A valid performance was confirmed by obtaining a low RMSD value (˂1) [45,46].

### 4.4. QSAR Study

A set of 10 derivatives of 1H-indol-3-yl compounds from antioxidant activities was assisted and examined by 2D-QSAR to evaluate the effect of its structure varieties. For further validation and experimental data you can refer to the supplementary file.

## 5. Conclusions

A new series of 3-(4-(thiophen-2-yl)-pyridin/pyran/pyrimidin/pyrazol-2-yl)-1*H*-indole derivatives were designed and synthesized as promising antioxidant candidates based on the introduction of equivalent reducing heterocyclic rings comparable to that of ascorbic acid. Applying a quantitative analysis of the structure-activity relationship (2D-QSAR) on candidates exhibited a various range of potentially promising antioxidant activities. Concerning ascorbic acid antioxidant activity, these synthesized compounds were categorized into three featured groups of antioxidants based on the results of their biological scavenging abilities against the evaluated radicals in vitro. Surprisingly, compound **10** was found to be more potent than ascorbic acid with IC_50_ = 28.23 μg/mL compared to that of ascorbic acid (IC_50_ = 30.03 μg/mL). It could be a promising lead compound, which via structural modification, led to the design and synthesis of novel powerful antioxidants. Furthermore, the mechanism of action for the new compounds was proposed as cytochrome *c* peroxidase inhibitors via molecular docking compared to ascorbic acid as a reference standard.

## Figures and Tables

**Table 1:** Antioxidant activities of the newly designed and synthesized indolyl derivatives.

Comp.	Conc (µM)						
	10	20	30	40	50	60	IC_50_
**10**	**21.6**	**36.1**	**50.3**	**59.7**	**71.0**	**84.5**	**28.23 ± 0.19**
**8**	18.7	31.9	40.2	57.3	69.1	83.5	31.71 ± 0.21
**3**	16.5	29.1	38.2	52.3	64.9	78.6	34.87 ± 0.22
**2**	13.0	23.7	30.1	42.6	54.8	64.1	45.26 ± 0.28
**7**	12.5	25.3	31.1	40.4	51.9	59.2	48.88 ± 0.24
**12b**	9.4	24.9	32.6	39.4	43.0	55.2	55.70 ± 0.31
**4**	10.8	21.2	27.6	34.5	39.0	48.7	68.83 ± 0.35
**12e**	19.1	23.1	28.7	35.2	39.4	52.1	71.06 ± 0.37
**12d**	7.8	18.6	26.2	31.4	35.7	42.5	80.87 ± 0.48
**Vit. C**	**20.1**	**36.3**	**47.5**	**60.7**	**70.2**	**82.7**	**30.23 ± 0.15**

**Table 2:** The binding scores, RMSD values, and receptor interactions of the two most promising compounds (**10** and **8**) compared to the docked ascorbic acid as a reference standard inside the binding pocket of cytochrome *c* peroxidase.

Compound	Score *	RMSD	Receptor Interactions	Distance *
**10**	−7.49	1.06	Ser185/H-donor	2.98
			His175/pi-pi	3.82
**8**	−7.24	0.77	Arg184/H-acceptor	2.89
			Ser185/H-acceptor	3.15
			His181/H-acceptor	3.19
			His175/H-pi	4.46
Ascorbic acid (docked)	−4.60	1.64	Met172/H-donor	3.21
			Met172/H-donor	3.29
			His175/H-acceptor	3.32

* The score unit is (kcal/mol), and the distance unit is (Å).

**Table 3:** 3D receptor interactions and positioning of the two most promising compounds (**10** and **8**) compared to the docked ascorbic acid as a reference standard inside the binding pocket of cytochrome *c* peroxidase.

Compound	3D Receptor Interactions	3D Receptor Positioning
**10**	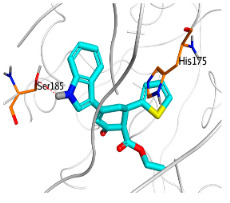	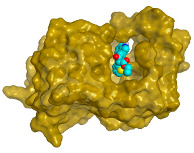
**8**	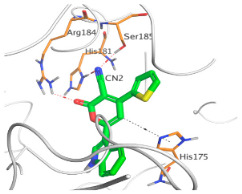	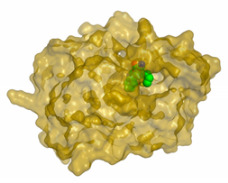
Ascorbic acid (docked)	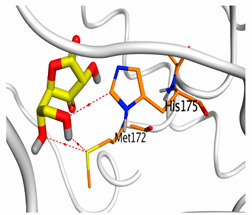	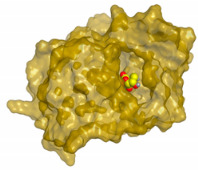

**Table 4:** Structures of reference compounds.

Code	Structure	Code	Structure
**4a**	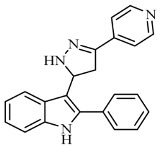	**6a**	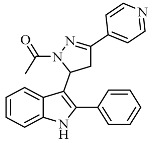
**4b**	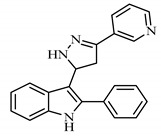	**6b**	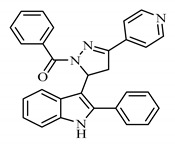
**4c**	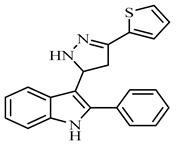	**6c**	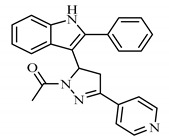
**5a**	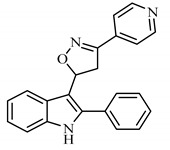	**6d**	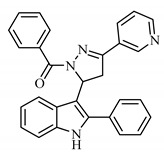
**5b**	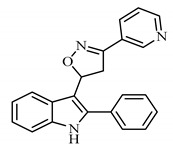	**7**	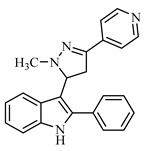
**5c**	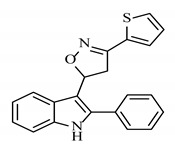	**Ascorbic acid**	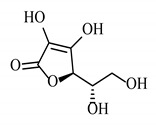

**Table 5:** References Compound and its Descriptors.

Mol.	IC50	AM1_ Dipole	AM1_E	AM1_ HF	AM1_ HOMO	AM1_ IP	AM1_ LUMO	b_Double	E	h_pKa	h_pKb
**4a**	21	5.03	−89,676.41	167.91	−8.35	8.35	−0.31	1.00	57.57	14.00	7.14
**4b**	35	2.99	−89,676.74	185.72	−8.30	8.30	−0.36	1.00	68.23	14.00	8.27
**4c**	24	3.73	−86,118.22	175.61	−8.25	8.25	−0.29	1.00	81.53	14.00	9.43
**5a**	70	5.23	−91,962.23	135.54	−8.50	8.50	−0.48	1.00	52.95	14.00	8.07
**5b**	117	3.22	−91,962.84	134.94	−8.47	8.47	−0.46	1.00	63.56	14.00	8.90
**5c**	233	3.57	−88,403.33	129.78	−8.40	8.40	−0.40	1.00	74.17	14.00	14.00
**6a**	29	8.10	−103,600.10	137.00	−8.62	8.62	−0.54	2.00	56.15	14.00	7.58
**6b**	53	5.27	−118,976.99	174.77	−8.60	8.60	−0.42	2.00	84.13	14.00	7.56
**6c**	29	5.53	−103,599.55	139.04	−8.73	8.73	−0.36	2.00	56.59	14.00	7.58
**6d**	50	3.20	−118,977.77	173.99	−8.56	8.56	−0.43	2.00	94.39	14.00	8.68
**7**	37	4.53	−93,258.02	173.10	−8.53	8.53	−0.19	1.00	51.55	14.00	7.00
**2**	42	4.12	−73,955.20	138.00	−8.45	8.45	−0.74	0.00	32.43	11.78	7.08
**10**	28	5.16	−99,419.59	−24.37	−8.48	8.48	−0.53	3.00	40.11	9.57	14.00
**3**	35	4.13	−76,858.14	96.20	−8.42	8.42	−0.79	0.00	9.91	9.09	7.46
**12b**	56	8.59	−124,440.79	180.53	−8.62	8.62	−1.77	4.00	84.13	18.12	8.21
**12d**	81	3.78	−124,441.94	178.35	−8.56	8.56	−1.51	4.00	78.68	18.18	8.28
**12e**	71	8.22	−116,256.55	135.79	−8.42	8.42	−0.94	3.00	61.33	9.78	7.67
**Ascorpic A.**	18	3.57	−64,592.94	−232.22	−9.81	9.81	−0.66	2.00	89.43	7.30	9.91

**Table 6:** Relative Importance of selected Descriptors.

Descriptor	Relative Importance
AM1_dipole	0.004654
AM1_E	0.402764
AM1_HF	1.000000
AM1_HOMO	0.000101
AM1_IP	0.000101
AM1_LUMO	0.000066
b_double	0.000231
E	0.650402
h_pKa	0.007998
h_pKb	0.019853

**Table 7:** The predicted antioxidant IC50.

Compound	Predicted IC50	Exp. IC50
**1**	69.38435583	--
**8**	49.09544469	31.71
**9**	74.55433474	--
**2**	51.26496301	45
**4**	27.77711207	68.83
**5**	79.93628873	--
**6**	77.74169132	--
**7**	47.80848428	48.88
**10**	23.05876527	28
**3**	31.84598687	35
**11**	53.56207022	--
**12a**	66.38183873	--
**12b**	65.65430383	56
**12c**	56.45276193	--
**12d**	62.64197628	81
**12e**	49.63524301	71
**Ascorbic acid**	32.91004419	26

## Data Availability

Not applicable.

## Supplementary Materials

The following are available online at https://www.mdpi.com/article/10.3390/ijms221910396/s1.
