# Supplementary material for: Design, Synthesis, Biological Evaluation, 2D-QSAR Modeling, and Molecular Docking Studies of Novel 1H-3-Indolyl Derivatives as Significant Antioxidants
Source: Int J Mol Sci. 2021 Sep 27;22(19):10396. doi: 10.3390/ijms221910396 (PMC8508798; doi:10.3390/ijms221910396)
Supplement: Supplementary file 1 [file ijms-22-10396-s001.zip › ijms-1344493-supplementary.pdf]

Design, Synthesis, Biological Evaluation, 2D-QSAR Modeling, and Molecular Docking Studies of Novel 1*H*-3-Indolyl Derivatives as Significant Antioxidants

Maged A. Aziz [1,*], Wesam S. Shehab [1], Ahmed A. Al-Karmalawy [2], Ahmed F. EL-Farargy [1] and Magda H. Abdellattif [3]

Supplementary document

Antioxidant activity

All chemicals included L-ascorbic acid were provided from Sigma-Aldrich (U.S.A.), and all solvents included annular ethanol delivered from El-NASR Co., Egypt. This assay follows the methodology of Re *et al*. ABTS method is a common assay in evaluation the potency of antioxidant activities of many pure organic candidates. ABTS method is a typical assay in evaluating the potency of antioxidant activities of many pure organic candidates. ABTS is an abbreviation of the chemical compound 2,2'-Azinobis(3-ethylbenzothiazoline-6-sulfonic acid). The chemical structure of this compound is very stable in free radical cation form ($ABTS^{\cdot+}$). $ABTS^{\cdot+}$ can react with any compound that generates a hydrogen atom (H-Donor) or an electron such as phenols and thiols, where $ABTS^{\cdot+}$ reacts as H or an electron acceptor **Figure S1**.

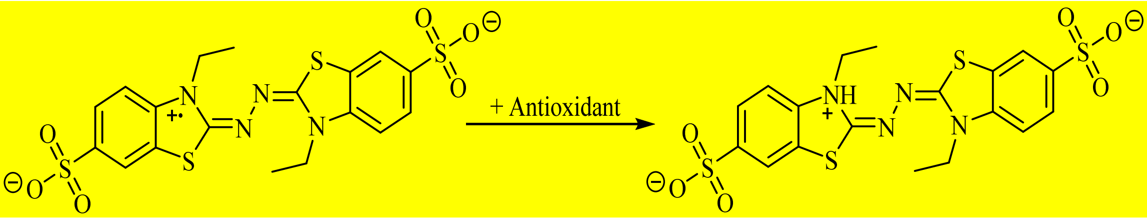

Figure S1. The reaction of $ABTS^{\cdot+}$ with antioxidant.

This transformation of hydrogen or electron converts $ABTS^{\cdot+}$ solution from a dark green one to a colorless solution. Equal amounts of $ABTS^{\cdot+}$ and potassium persulphate $K_2S_2O_8$ (7 mM & 3.5 mM, respectively) were added from their stock solutions to make the $ABTS^{\cdot+}$ standard solution. The yielded mixture was left to stand at room temperature in the darkness for 13-15 h overnight. The $ABTS^{\cdot+}$ stock solution is appropriate for use after the completion of the reaction. The evidence of finishing the reaction is the stability of the spectrophotometric absorbance of the $ABTS^{\cdot+}$ solution at a wavelength of 735 nm. $ABTS^{\cdot+}$ stock solution may be stored at room temperature in

the darkness for about 2-3 days and still valid over this period to use. The present assay was prepared the $ABTS^{\cdot+}$ working solution from stock one by dilution in annular ethanol to get an absorbance A_{blank} of 0.7 ± 0.02 at a wavelength of 735 nm. In an incubator, the produced solution was controlled at a temperature of 30 °C and was equilibrated. In this assay, A_{blank} was adapted to be accurately 0.7 at time = 0, i.e., before the determination of the absorbance for all the targeted compounds. In order to measure the scavenging activities of the targeted compounds against the free radicals 1.5 ml of dark blue $ABTS^{\cdot+}$ working solution was mixed with 10 µl of the compounds solutions (**2**, **3**, **4**, **7**, **8**, **10**, **12b**, **12d**, **12e**). These steps are repeated using various concentrations of the tested compounds solutions over a range of 10-300 µM. These various concentrations are achieved by dilution using distilled water and annular ethanol or both based on their solubility degree. After adding ABTS·+ solution with different tested candidates, the change in absorbance value was determined over various rang of time at 0, 0.5, 1, and 5 mins in order to manage to achieve the steady-state value of absorbance. In our present assay, the steady-state value was achieved after 15 min. By generalization, A_{test}, absorbance value for each tested candidate, was reported after 15 min of addition $ABTS^{\cdot+}$ solution tested candidate solution. The mean of values was recorded. Each concentration for each tested sample at a specific time was recorded separately for all compounds, then every three separate measurements were determined, and the mean was taken. The antioxidant activity of each compound against $ABTS^{\cdot+}$ was calculated from the percent reduction in absorbance values, according to the following equation:

$ABTS^{\cdot+}$ radical cation scavenging activity of test compound (%) = $100(A_{blank}-A_{test})/A_{blank}$.

Where,

A_{test} or A_{15} = The absorbance value for each tested candidate after 15 min of addition $ABTS^{\cdot+}$ solution.

A_{blank} or A_0 = The absorbance value of $ABTS^{\cdot+}$ itself before adding the tested candidates.

i.e., Time = 0. (A_{blank} was adjusted to be 0.70)

In this assay, after 15 minutes of reaction, the IC50 (inhibitory concentration 50 percent) of each of the test compounds was determined and calculated by Unico spectrophotometer 1200 USA and compared to that of L-ascorbic acid as the reference and standard.

QSAR Study

Data set for QSAR studies

A set of 10 derivatives of 1*H*-indol-3-yl compounds from antioxidant activities was assisted and examined by 2D-QSAR to evaluate the effect of its structure varieties. Initially, to split data into training and test 11 compounds, *Kennard Stone* (ks) statistic method was used for data validation of 7 compounds, and about 80% of the data set was distributed as training and the remaining as a test set during the use of this application. The negative logarithmic scale (pIC50 = −log IC50) was used. The 2D structural coordinates of descriptive molecules were sketched and saved in mol2. These compounds' structures were transferred into the MOE database file using the MOE-DB option, which converted coordinates of all the compounds into 3D structures by MOE wash module. Using MOE default wash command, partial charges were performed on the compounds, and their energy was corrected using the current MMFF94 force field.

Molecular descriptors

Several 2D and 3D structural fragments and graph invariants contribute by MOE to determine the physicochemical properties of compounds. For this purpose, all the compounds (both training and test set) with some 2D descriptors present in MOE were calculated. Subsequently, "QuaSAR-Contingency," a statistical option in MOE was applied for the selection of suitable descriptors for QSAR modeling. The contingency analysis proposed 10 2D descriptors including AM1_Dipole, a descriptor for Total energy (kCal/mol)(AM1_E, a descriptor for the heat of formation calculated by empirical method (AM1-HF), descriptor for calculating the energy of the lowest unoccupied molecular orbital by empirical method (AM1-LUMO), descriptor for calculating the energy of highest occupied molecular orbital by empirical method (AM1-HOMO), ionization potential(eV)(AM_IP), number of double bonds (b_double), descriptor of acidity at pH=7(h_pKa), and the descriptor of basicity at pH=7(h_pKb), were calculated. Consequently, the QSAR model was obtained by the partial least squares (PLS) method.

Experimental data model development

QSAR model was improved based on the actual antioxidant activities (as dependant variable) and the descriptors as model fields. For the training data set, regression analysis was performed, and r^2 root means square error (RMSE) values of the fit were reported. This fit model was saved as the QSAR model and used for the prediction of activities of compounds of the test data set.

Cross-Validating the model

The durability and power of the above QSAR fit were adopted for both model validation and cross-validation. These validation metrics estimate the predictive activities and the residual for the training set molecules. The predicted, residual, and Z-score values of acceptable thresholds were calculated for both model and cross-validations.

Graphical analysis

The predictive capability of the model was evaluated using a correlation plot by plotting the values on the x-axis and the predicted IC50 activity on the y-axis. This correlation plot was used to identify outliers that have a Z-score beyond the range of 2.

Docking study

A molecular docking study of the sixteen newly designed and synthesized indolic compounds was performed using MOE 2019.0102 program. The newly synthesized compounds were drawn using ChemDraw, imported into the MOE program window, converted for their 3D forms, adjusted for the partial charges, and energy minimized as described earlier. The database was built containing the newly synthesized candidates (**1-12a-e**) together with the co-crystallized ascorbic acid as a reference standard. A general docking process was performed using the site of the co-crystallized ascorbic acid inside cytochrome *c* peroxidase as the docking site. Also, all the other docking parameters were adjusted as previously discussed in detail. Moreover, it is worth mentioning that a program validation process was performed at first before applying the docking process by

redocking the co-crystallized ascorbic acid at its binding pocket of the cytochrome *c* peroxidase enzyme. A valid performance was confirmed by obtaining a low RMSD value (<1).

Chemistry

All chemicals included 3-Acetylindole were provided from Sigma-Aldrich (U.S.A.), and all solvents included annular ethanol delivered from El-NASR Co., Egypt. All the reactions were carried out with readily available reagents that were utilized without additional purification as received. Digital Electrothermal IA 9100 Series apparatus Cole-Parmer, Beacon Road, Stone, Staffordshire, ST15 OSA, UK) used to measure the melting points that they are uncorrected. The mass spectrum was recorded in the Thermo scientific GCMS model (ISQLT) via the direct probe controller inlet part to a single quadrupole mass analyser utilizing Thermo X-Cali bur software, at Al-Azhar University, (RCMB), Naser City, Cairo. IR spectra were carried out in the range from 4000 to 400 cm^{-1} on (Thermo-Fisher Scientific) FT-IR PLUS spectrometer (υ by cm-1) utilizing potassium bromide disks (KBr) at the microanalytical Laboratory, Faculty of Science, Cairo University, Egypt. C, H, and N analyses were carried out on a PerkinElmer CHN 2400. ^{1}H and ^{13}C-NMR spectra were carried out on a Bruker NMR spectrophotometer at 400 MHz in DMSO-d_6 using tetramethylsilane (TMS) as the internal reference standard, chemical shifts are expressed in δ which given in parts per million (ppm), and DMSO-d_6 was used as the solvent.

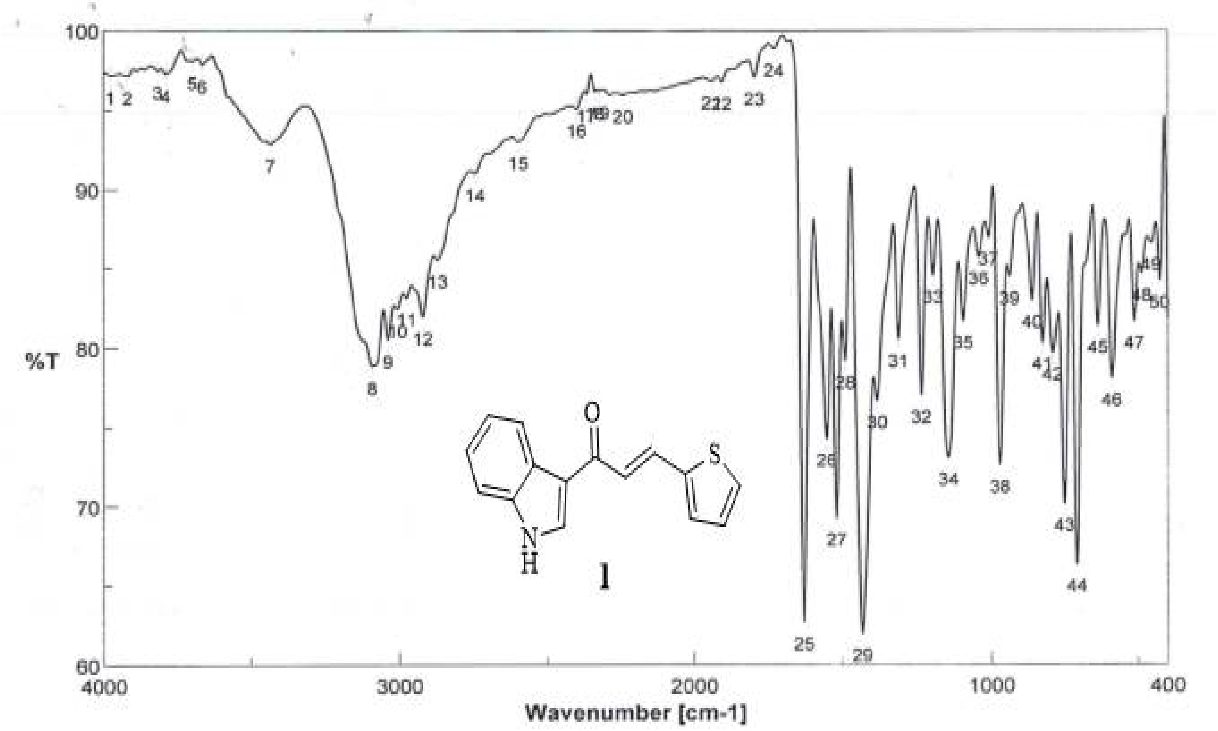

Accumulation	16
Resolution	4 cm-1
Zero Filling	ON
Apodization	Cosine
Gain	Auto (8)
Scanning Speed	Auto (2 mm/sec)
Date/Time	8/27/2020 11:03AM
Update	8/27/2020 11:06AM
Operator	IR
File Name	Memory#46
Sample Name	W1
Comment	

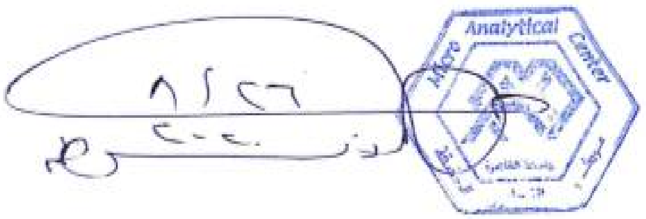

IR spectrum for compound 1

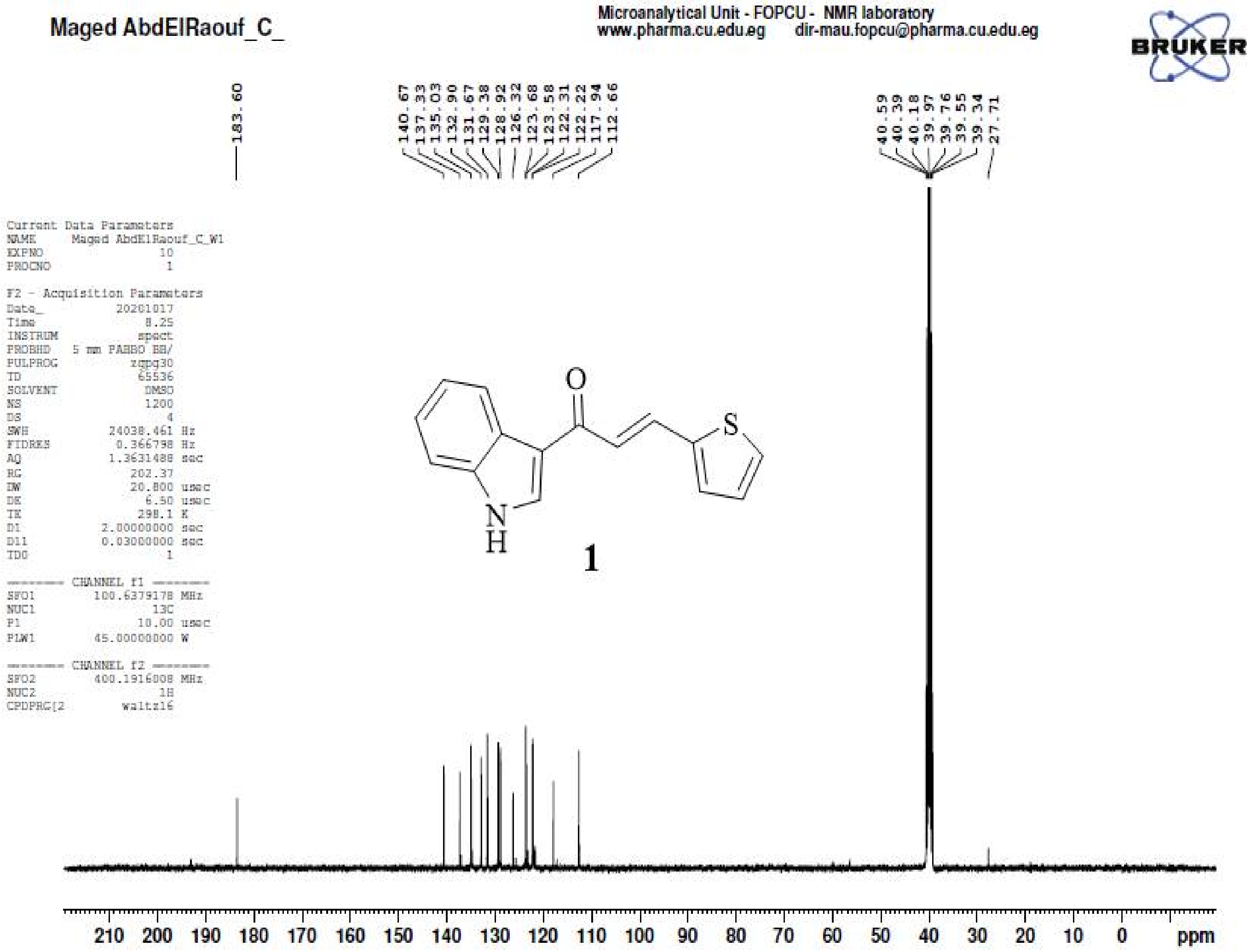

^{13}C-NMR spectrum for compound 1

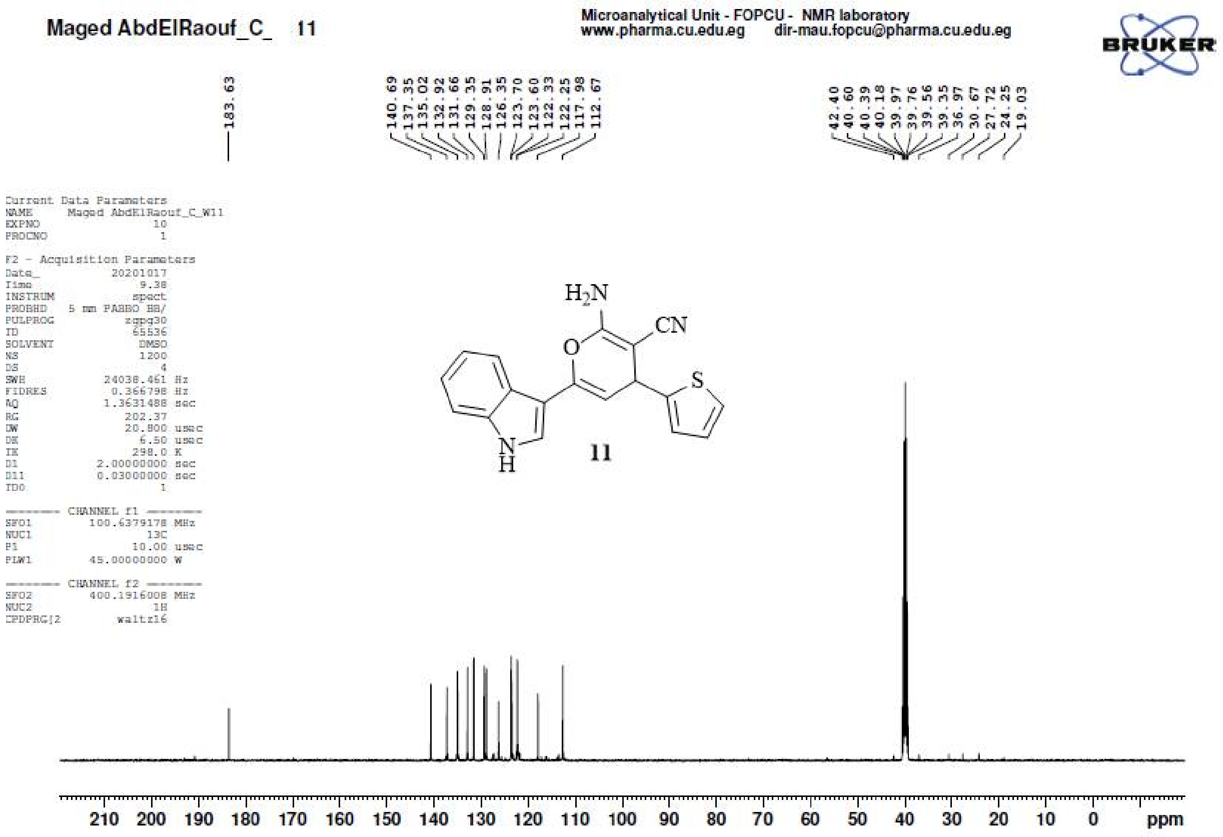

^{13}C-NMR spectrum for compound 1

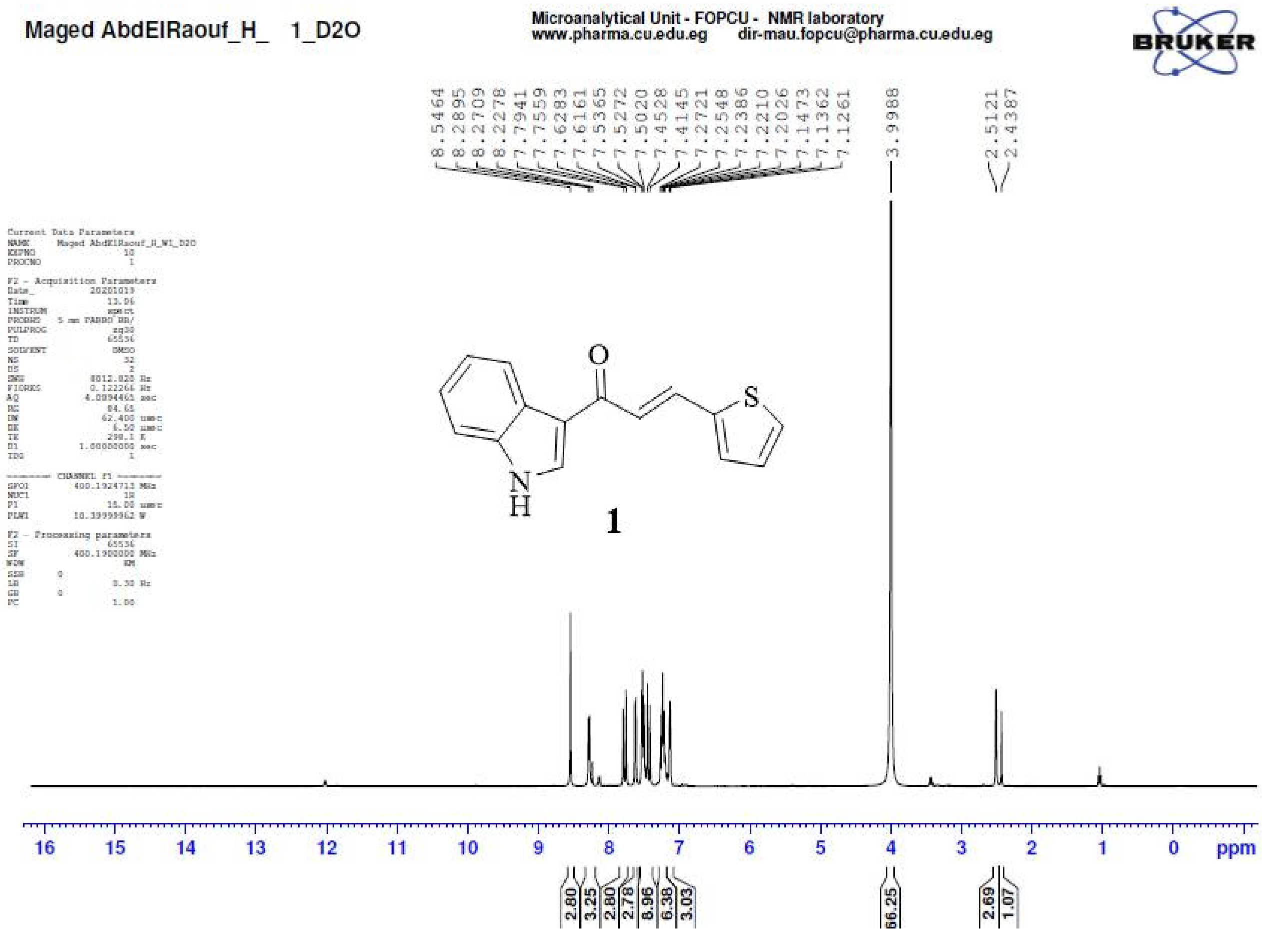

^{1}H-NMR (D_2O) spectrum for compound 1

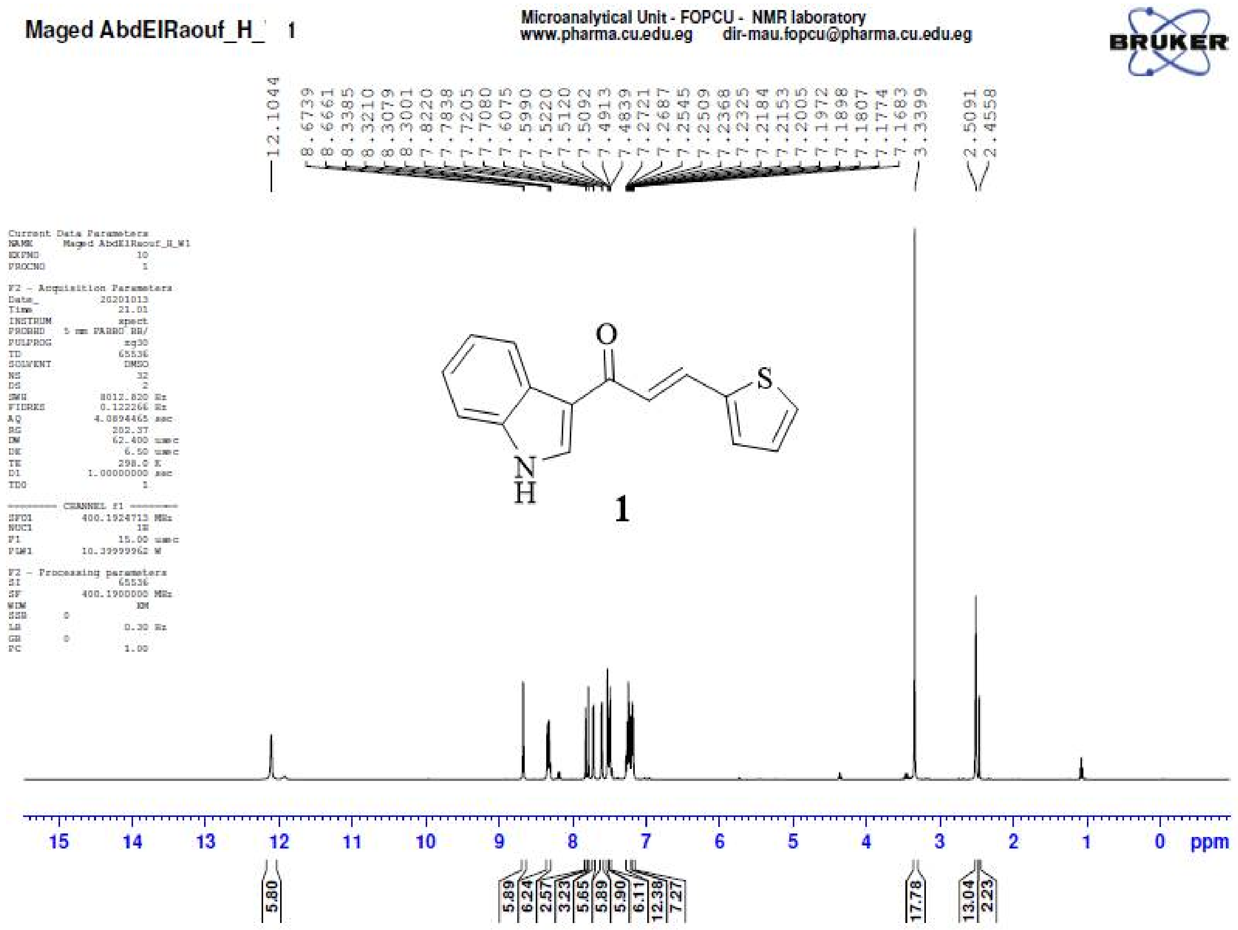

^{1}H-NMR spectrum for compound 1

23-Jan-07 20:32:55

Cairo University
Micro Analytical Center

DI Analysis
Shimadzu Qp-2010 Plus

Sample Information

Analyzed by	: Dr. Mai Younis
Analyzed	: 23/01/2007 07:52:21 م
Sample Name	: W1
Sample ID	:
Customer Name	: Dr. Abdou Othman - Science - Cairo
Data File	: C:\GCMSsolution\Data\Project1\W1 .QGD
Org Data File	: C:\GCMSsolution\Data\Project1\W1 .QGD
Method File	: C:\GCMSsolution\Data\Project1\High Temperature Op
Org Method File	: C:\GCMSsolution\Data\Project1\High Temperature Op
Report File	:
Tuning File	: C:\GCMSsolution\System\Tune1_default.qgt
SEndIfSModified by	: Dr. Mai Younis
Modified	: 23/01/2007 07:58:10 م

Method

===== Analytical Line 1 =====

IonSourceTemp	:250.00 °C
[MS Table]	
--Group 1 - Event 1--	
Start Time	:0.00min
End Time	:10.00min
ACQ Mode	:Scan
Event Time	:0.50sec
Scan Speed	:1000
Start m/z	:50.00
End m/z	:500.00
Electron Voltage	: 70 eV
Ionization Mode	: EI

C:\GCMSsolution\Data\Project1\W1 QGD

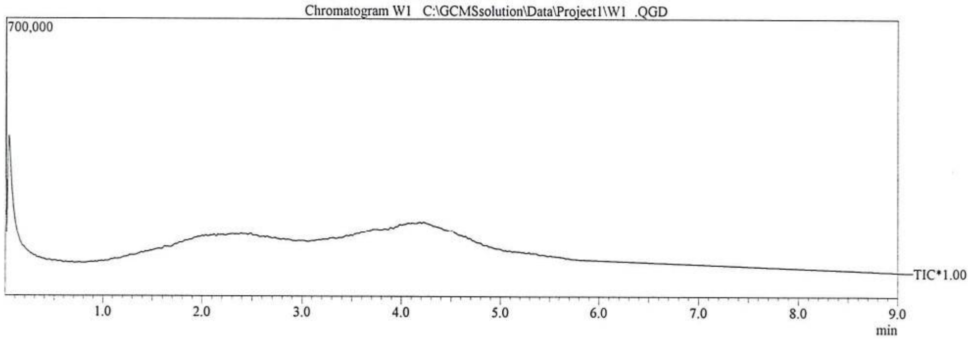

Spectrum

Line#:1 R.Time:3.2(Scan#:379)
MassPeaks:129
RawMode:Single 3.2(379) BasePeak:57(9439)
BG Mode:None Group 1 - Event 1

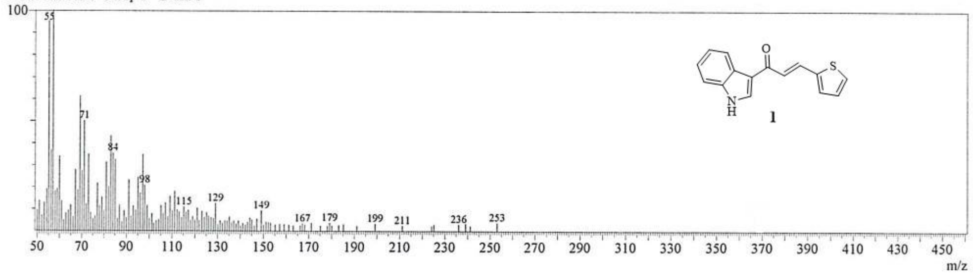

Mass spectrum for compound 1

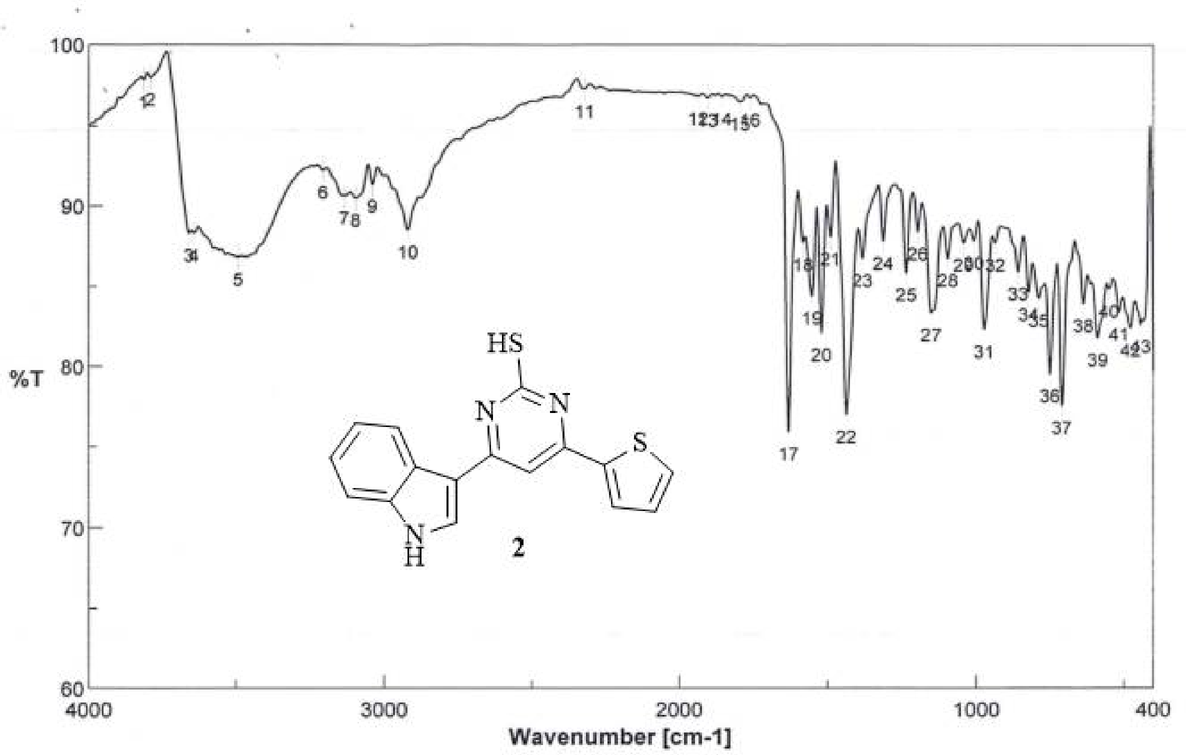

Accumulation	16
Resolution	4 cm-1
Zero Filling	ON
Apodization	Cosine
Gain	Auto (8)
Scanning Speed	Auto (2 mm/sec)
Date/Time	8/27/2020 11:09AM
Update	8/27/2020 11:11AM
Operator	IR
File Name	Memory#59
Sample Name	W4
Comment	

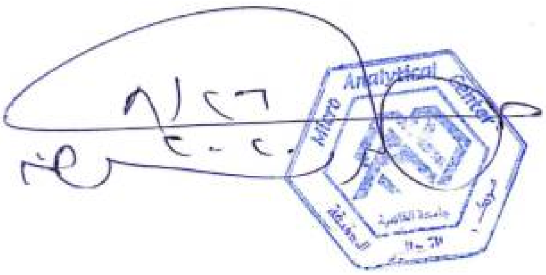

IR spectrum for compound 2

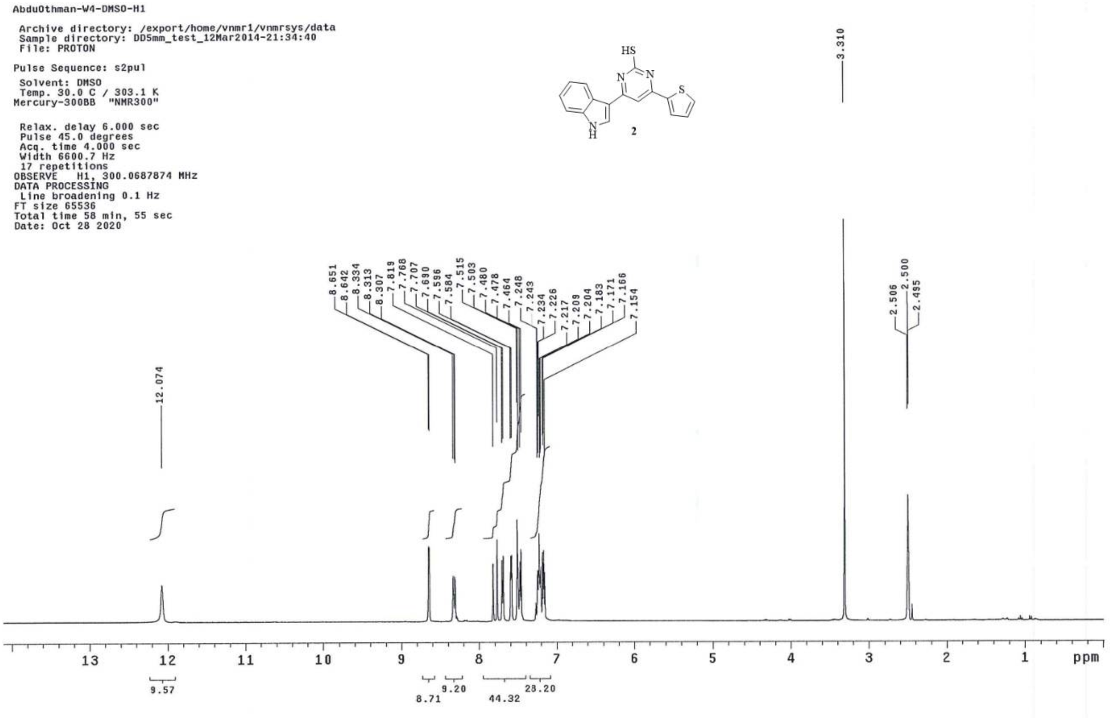

^{1}H-NMR spectrum for compound 2

23-Jan-07

Cairo University
Micro Analytical Center

DI Analysis
Shimadzu Qp-2010 Plus

Sample Information

Analyzed by	: Dr. Mai Younis
Analyzed	: 23/01/2007 07:27:40 م
Sample Name	: W2.
Sample ID	:
Customer Name	: Dr. Abdou Othman - Science - Cairo
Data File	: C:\GCMSsolution\Data\Project1\W2..QGD
Org Data File	: C:\GCMSsolution\Data\Project1\W2..QGD
Method File	: C:\GCMSsolution\Data\Project1\High Temperature Op
Org Method File	: C:\GCMSsolution\Data\Project1\High Temperature Op
Report File	:
Tuning File	: C:\GCMSsolution\System\Tune1_default.qgt
$EndIf$Modified by	: Dr. Mai Younis
Modified	: 23/01/2007 07:27:13 م

Method

===== Analytical Line 1 =====

IonSourceTemp	:250.00 °C
[MS Table]	
--Group 1 - Event 1--	
Start Time	:0.00min
End Time	:10.00min
ACQ Mode	:Scan
Event Time	:0.50sec
Scan Speed	:1000
Start m/z	:50.00
End m/z	:500.00
Electron Voltage	: 70 eV
Ionization Mode	: EI

C:\GCMSsolution\Data\Project1\W2..QGD

Chromatogram W2. C:\GCMSsolution\Data\Project1\W2..QGD

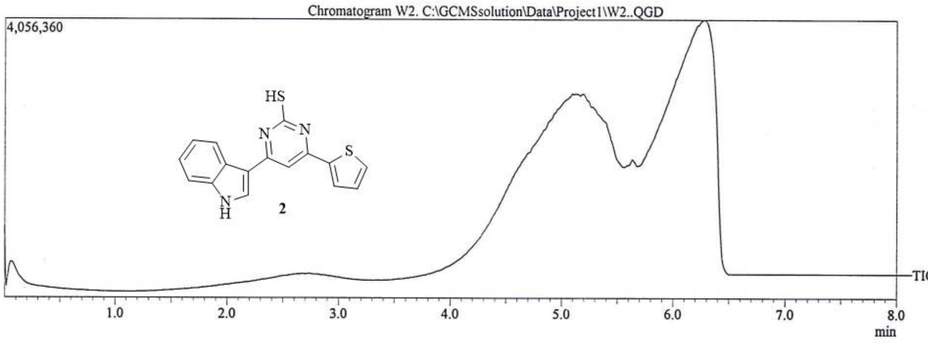

Spectrum

Line#:1 R.Time:5.2(Scan#:619)
MassPeaks:216
RawMode:Single 5.2(619) BasePeak:253(271556)
BG Mode:None Group 1 - Event 1

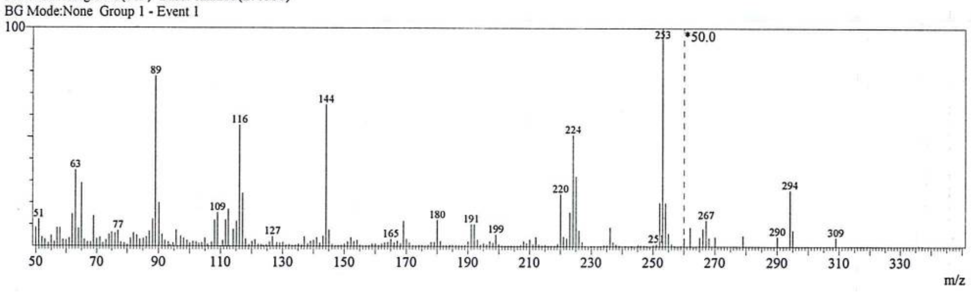

Mass spectrum for compound 2

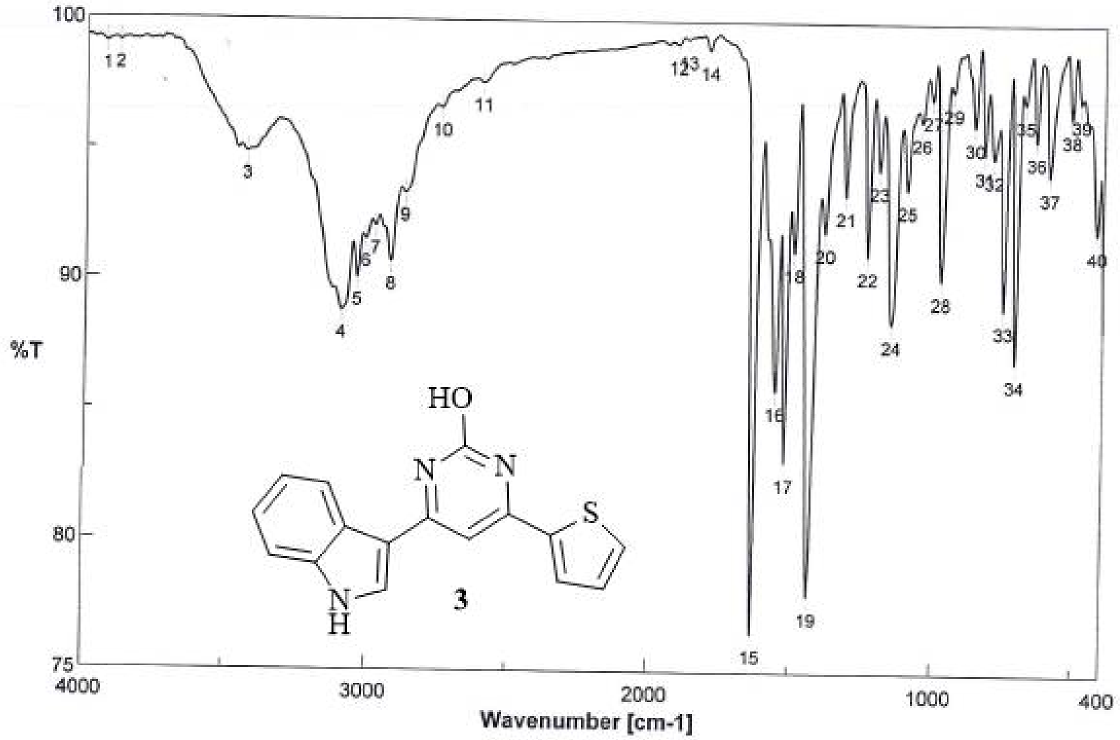

Accumulation	16
Resolution	4 cm-1
Zero Filling	ON
Apodization	Cosine
Gain	Auto (2)
Scanning Speed	Auto (2 mm/sec)
Date/Time	10/20/2020 0:45PM
Update	10/20/2020 0:46PM
Operator	
File Name	Memory#146
Sample Name	W 10
Comment	

Micro Analytical Center

IR spectrum for compound 3

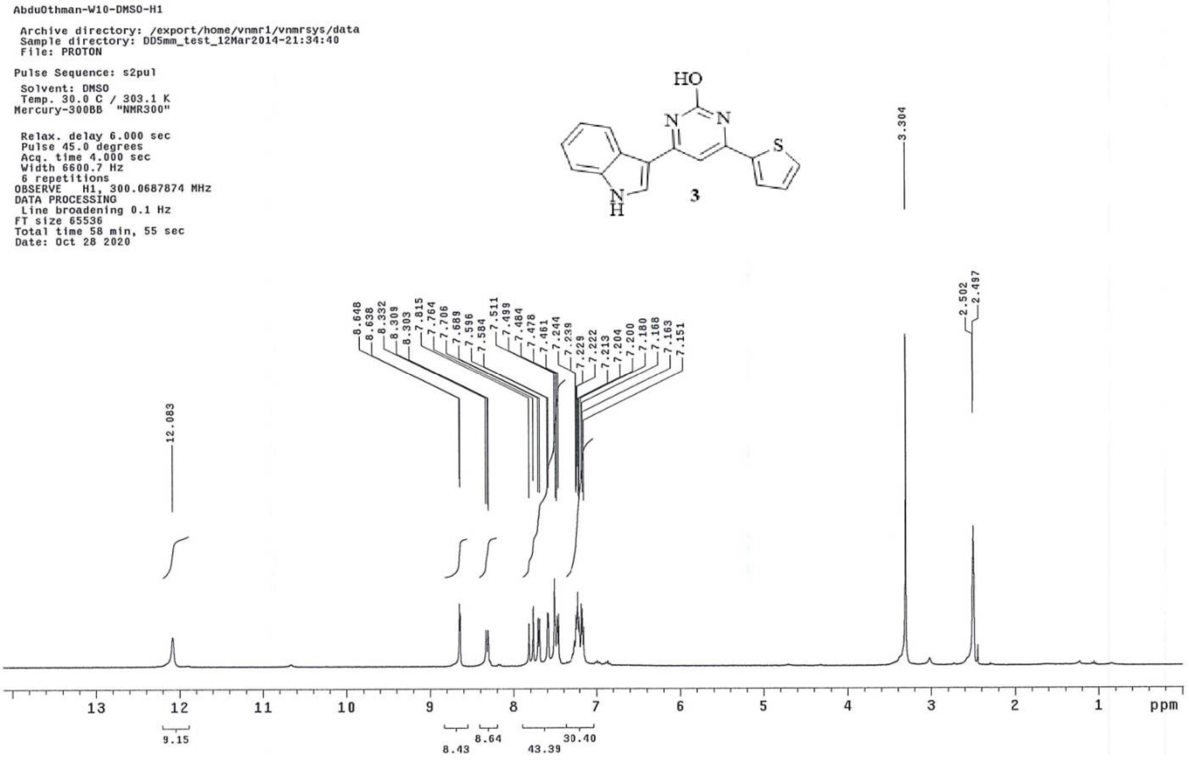

^{1}H-NMR spectrum for compound 3

23-Jan-07

Cairo University
Micro Analytical Center

DI Analysis
Shimadzu Qp-2010 Plus

Sample Information

Analyzed by	: Dr. Mai Younis
Analyzed	: 23/01/2007 07:20:40 م
Sample Name	: W3
Sample ID	:
Customer Name	: Dr. Abdou Othman - Science - Cairo
Data File	: C:\GCMSsolution\Data\Project1\W3 .QGD
Org Data File	: C:\GCMSsolution\Data\Project1\W3..QGD
Method File	: C:\GCMSsolution\Data\Project1\High Temperature Op
Org Method File	: C:\GCMSsolution\Data\Project1\High Temperature Op
Report File	:
Tuning File	: C:\GCMSsolution\System\Tune1_default.qgt
SEndIf$Modified by	: Dr. Mai Younis
Modified	: 23/01/2007 07:27:13 م

Method

===== Analytical Line 1 =====

IonSourceTemp	:250.00 °C
[MS Table]	
--Group 1 - Event 1--	
Start Time	:0.00min
End Time	:10.00min
ACQ Mode	:Scan
Event Time	:0.50sec
Scan Speed	:1000
Start m/z	:50.00
End m/z	:500.00
Electron Voltage	: 70 eV
Ionization Mode	: EI

C:\GCMSsolution\Data\Project1\W3..QGD

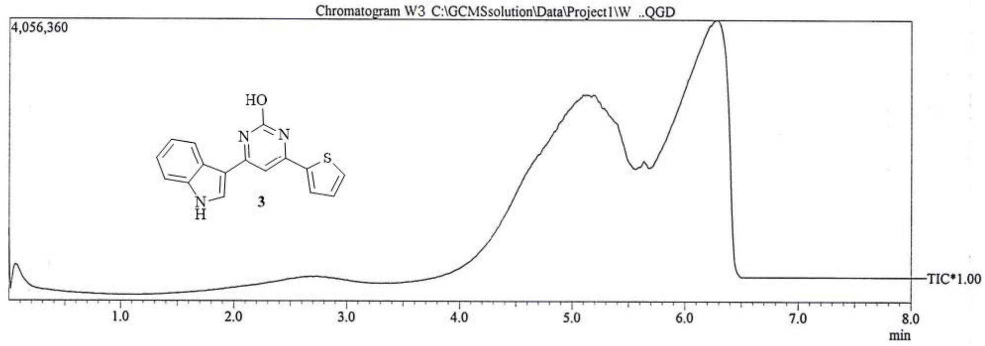

Spectrum

Line#:1 R.Time:5.2(Scan#:619)
MassPeaks:216
RawMode:Single 5.2(619) BasePeak:253(271556)
BG Mode:None Group 1 - Event 1

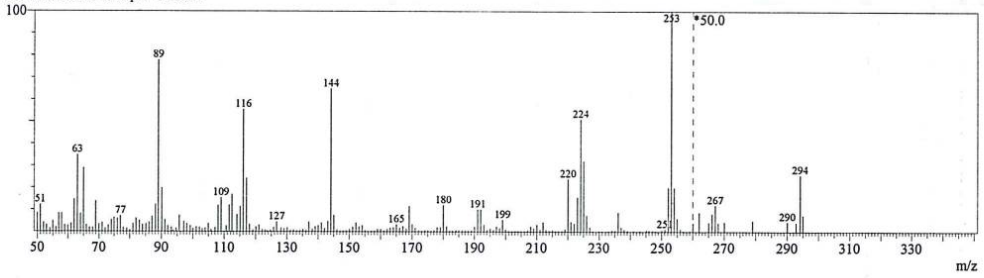

Mass spectrum for compound 3

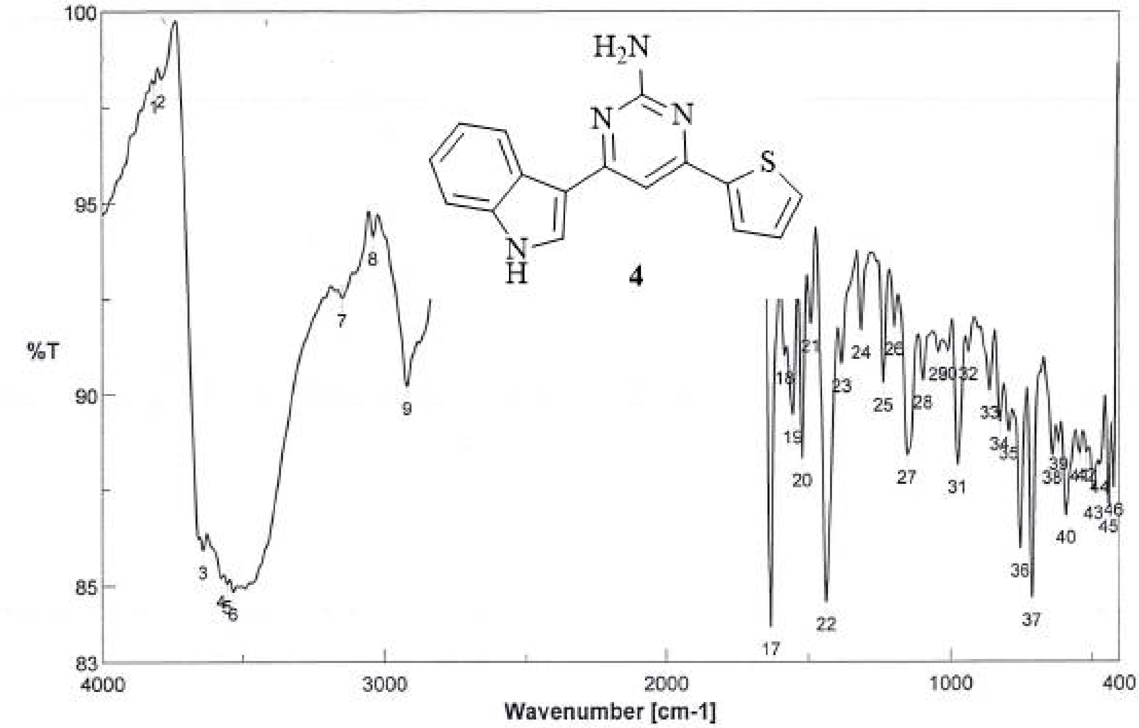

Accumulation	16
Resolution	4 cm-1
Zero Filling	ON
Apodization	Cosine
Gain	Auto (8)
Scanning Speed	Auto (2 mm/sec)
Date/Time	8/27/2020 11:14AM
Update	8/27/2020 11:16AM
Operator	IR
File Name	Memory#75
Sample Name	W5
Comment	

IR spectrum for compound 4

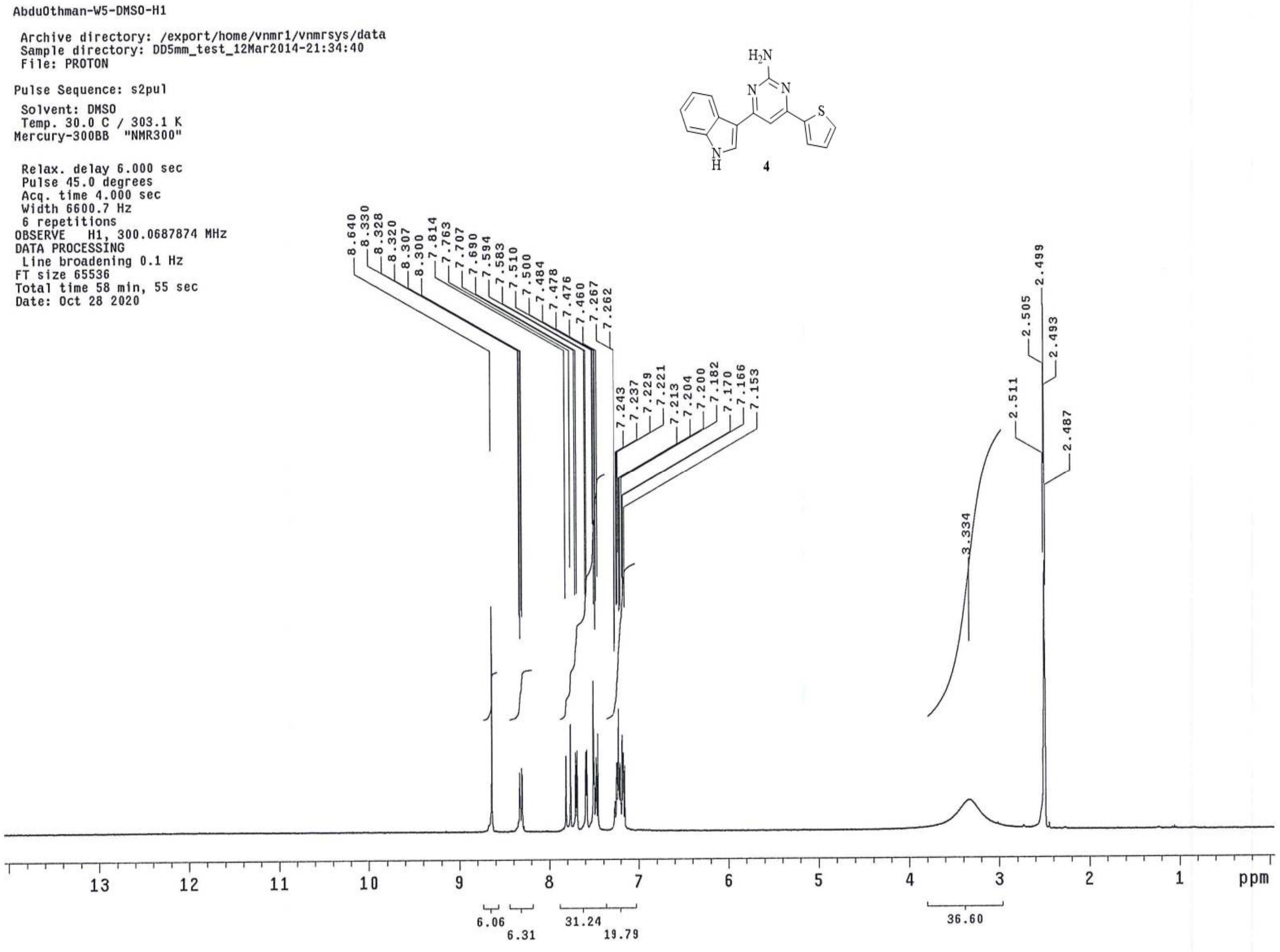

^{1}H-NMR spectrum for compound 4

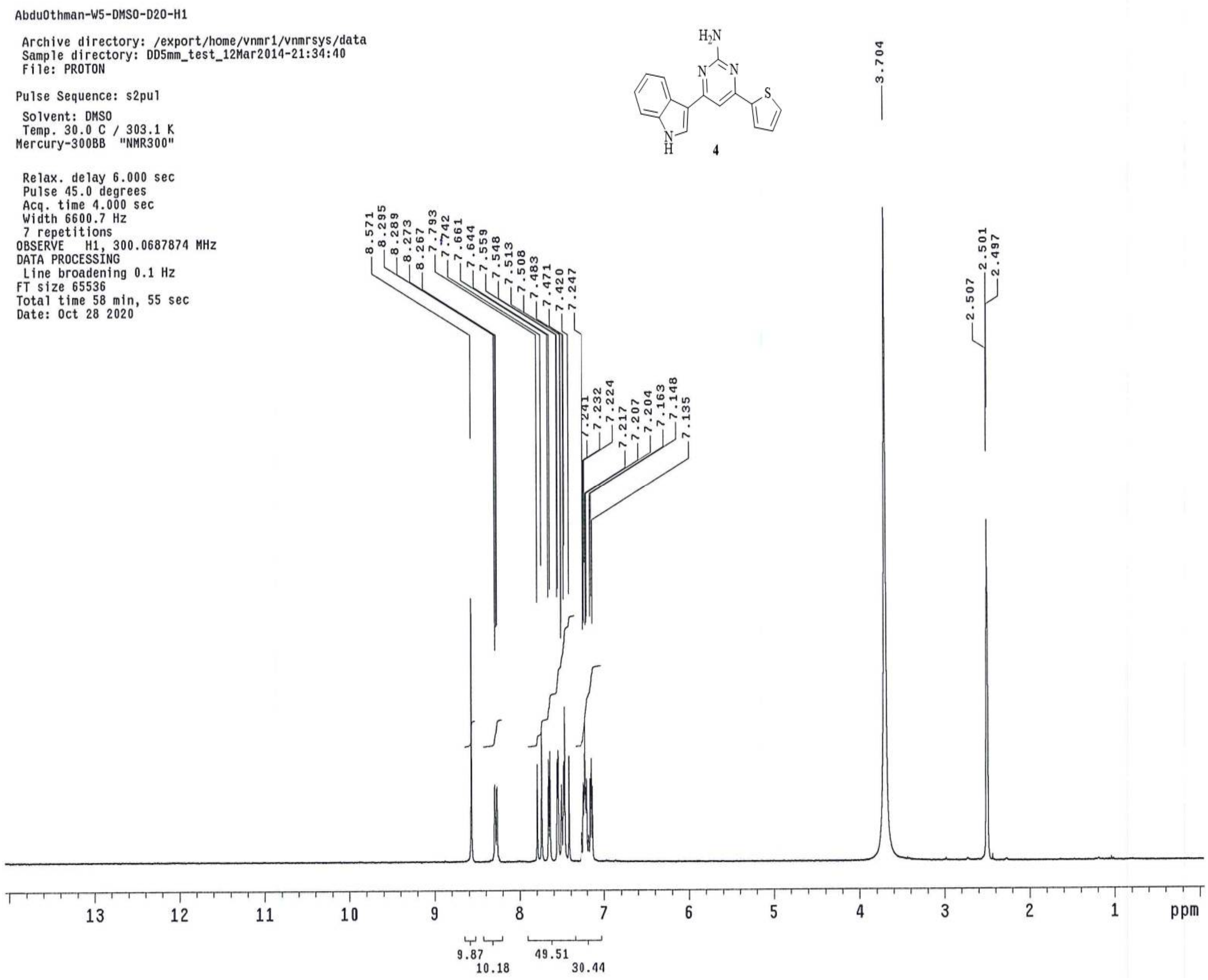

^{1}H-NMR spectrum for compound 4

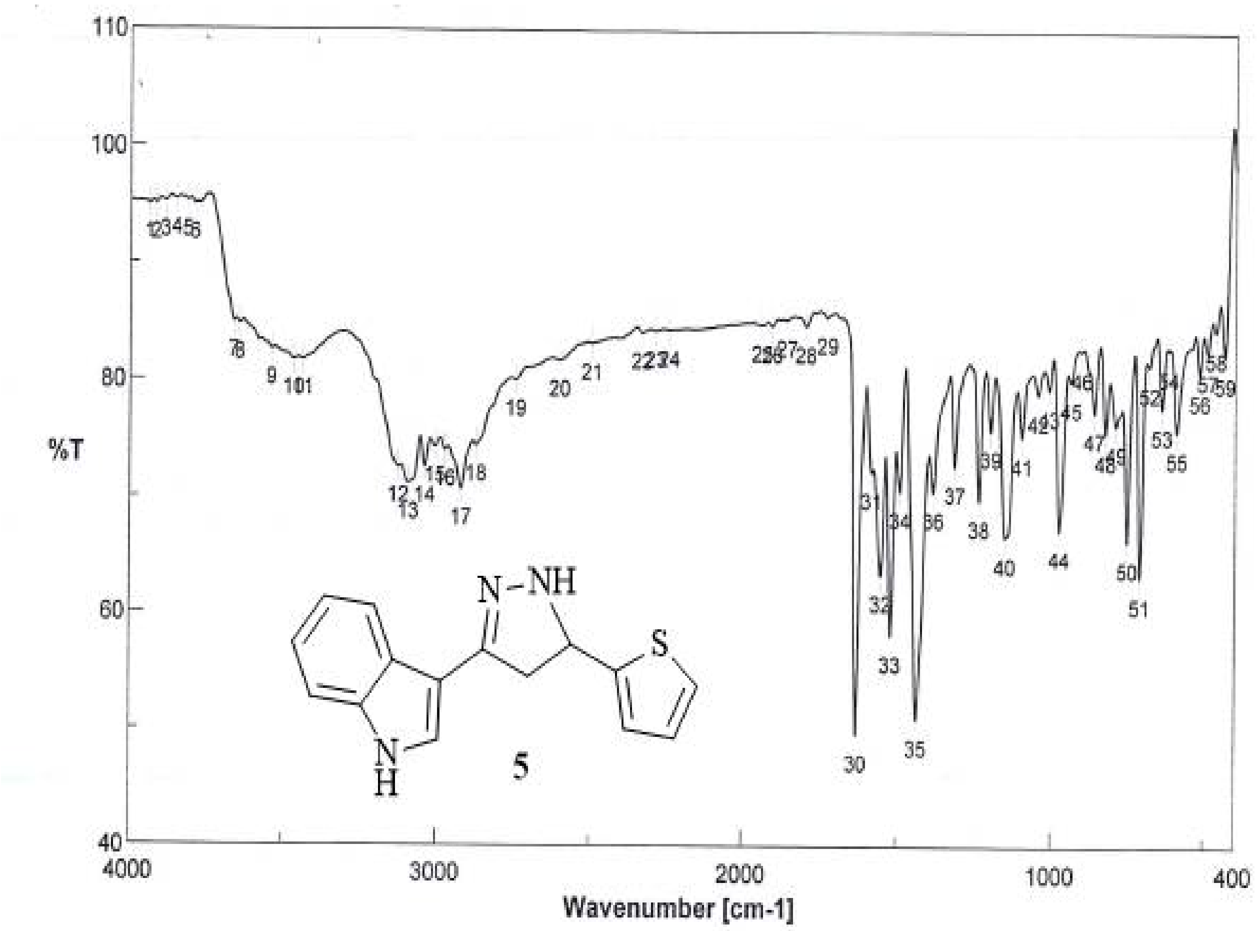

Accumulation	16
Resolution	4 cm-1
Zero Filling	ON
Apodization	Cosine
Gain	Auto (8)
Scanning Speed	Auto (2 mm/sec)
Date/Time	8/27/2020 11:14AM
Update	8/27/2020 11:14AM
Operator	IR
File Name	Memory#69
Sample Name	W6
Comment	

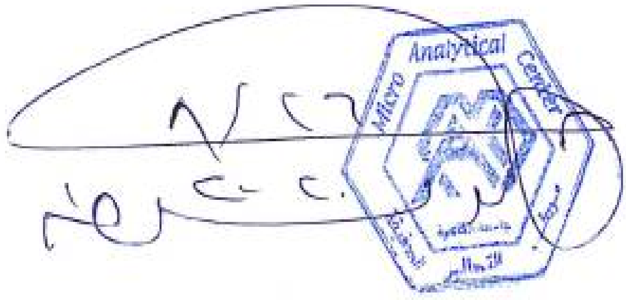

IR spectrum for compound 5

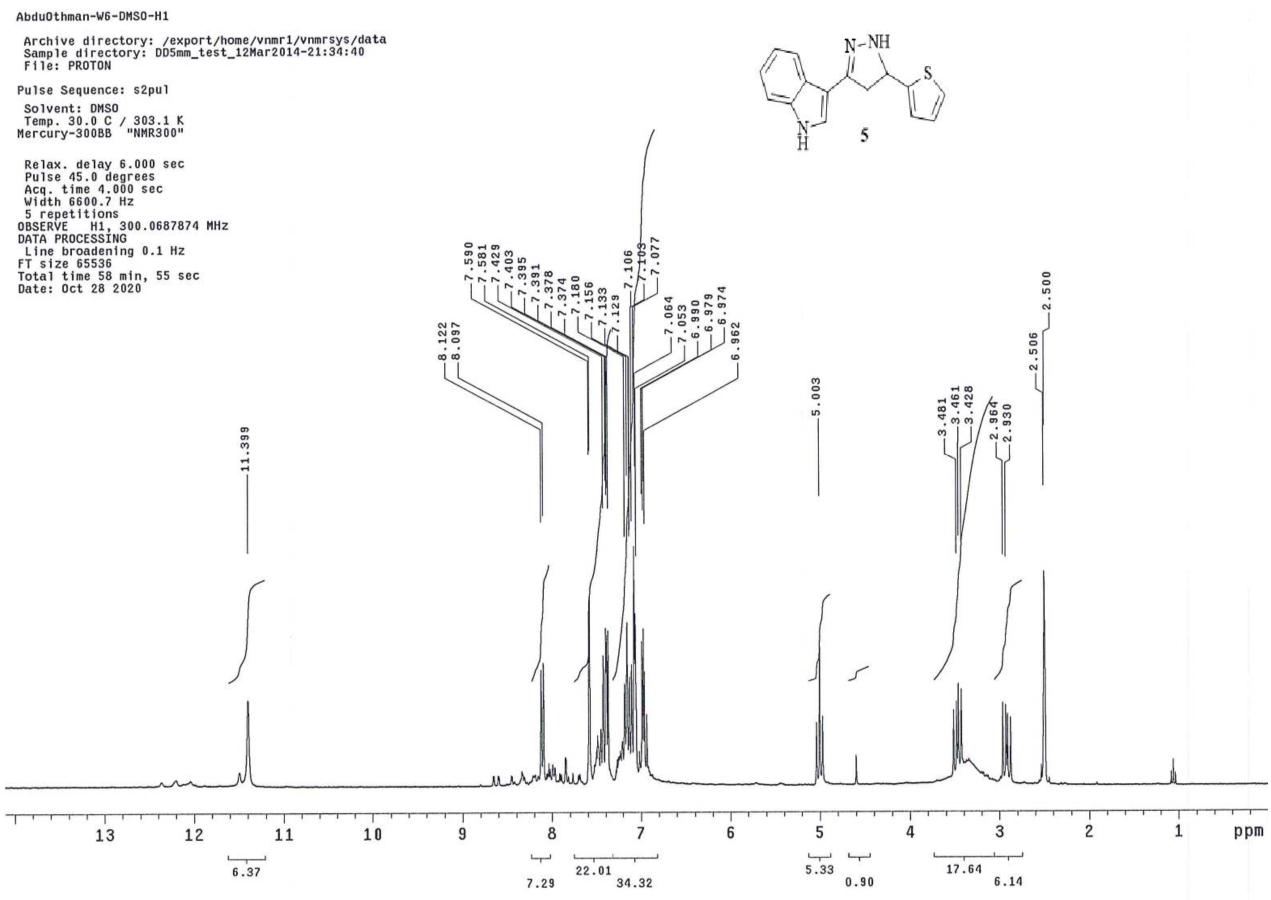

^{1}H-NMR spectrum for compound 5

23-Jan-07

Cairo University
Micro Analytical Center

DI Analysis
Shimadzu Qp-2010 Plus

Sample Information

Analyzed by : Dr. Mai Younis
Analyzed : 23/01/2007 07:20:40 م
Sample Name : W5
Sample ID :
Customer Name : Dr. Abdou Othman - Science - Cairo
Data File : C:\GCMSsolution\Data\Project1\W5..QGD
Org Data File : C:\GCMSsolution\Data\Project1\W5..QGD
Method File : C:\GCMSsolution\Data\Project1\High Temperature Op
Org Method File : C:\GCMSsolution\Data\Project1\High Temperature Op
Report File :
Tuning File : C:\GCMSsolution\System\Tune1_default.qgt
$EndIf$Modified by : Dr. Mai Younis
Modified : 23/01/2007 07:27:13 م

Method

===== Analytical Line 1 =====
IonSourceTemp :250.00 °C
[MS Table]
--Group 1 - Event 1--
Start Time :0.00min
End Time :10.00min
ACQ Mode :Scan
Event Time :0.50sec
Scan Speed :1000
Start m/z :50.00
End m/z :500.00

Electron Voltage : 70 eV
Ionization Mode : EI

C:\GCMSsolution\Data\Project1\W5..QGD

Chromatogram W5. C:\GCMSsolution\Data\Project1\W ..QGD

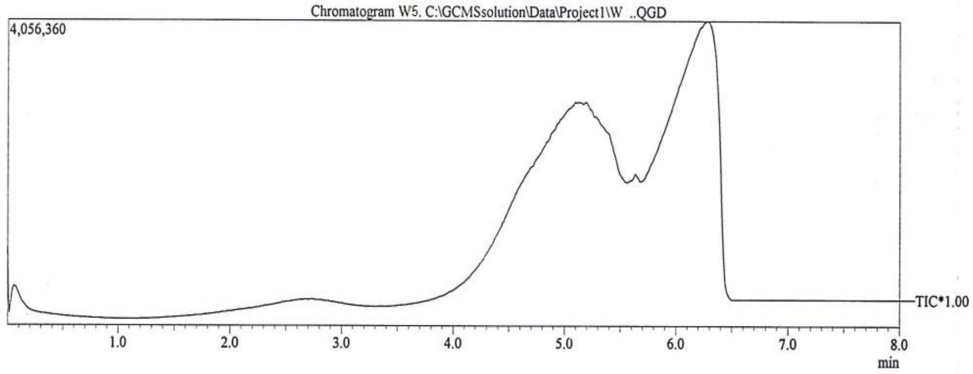

Spectrum

Line#:1 R.Time:5.2(Scan#:619)
MassPeaks:216
RawMode:Single 5.2(619) BasePeak:253(271556)
BG Mode:None Group 1 - Event 1

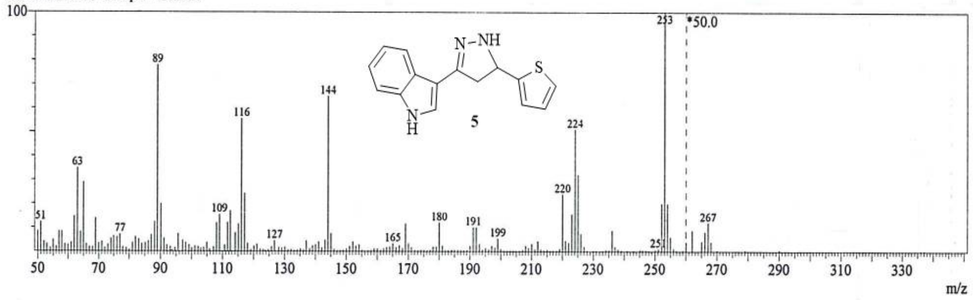

Mass spectrum for compound 5

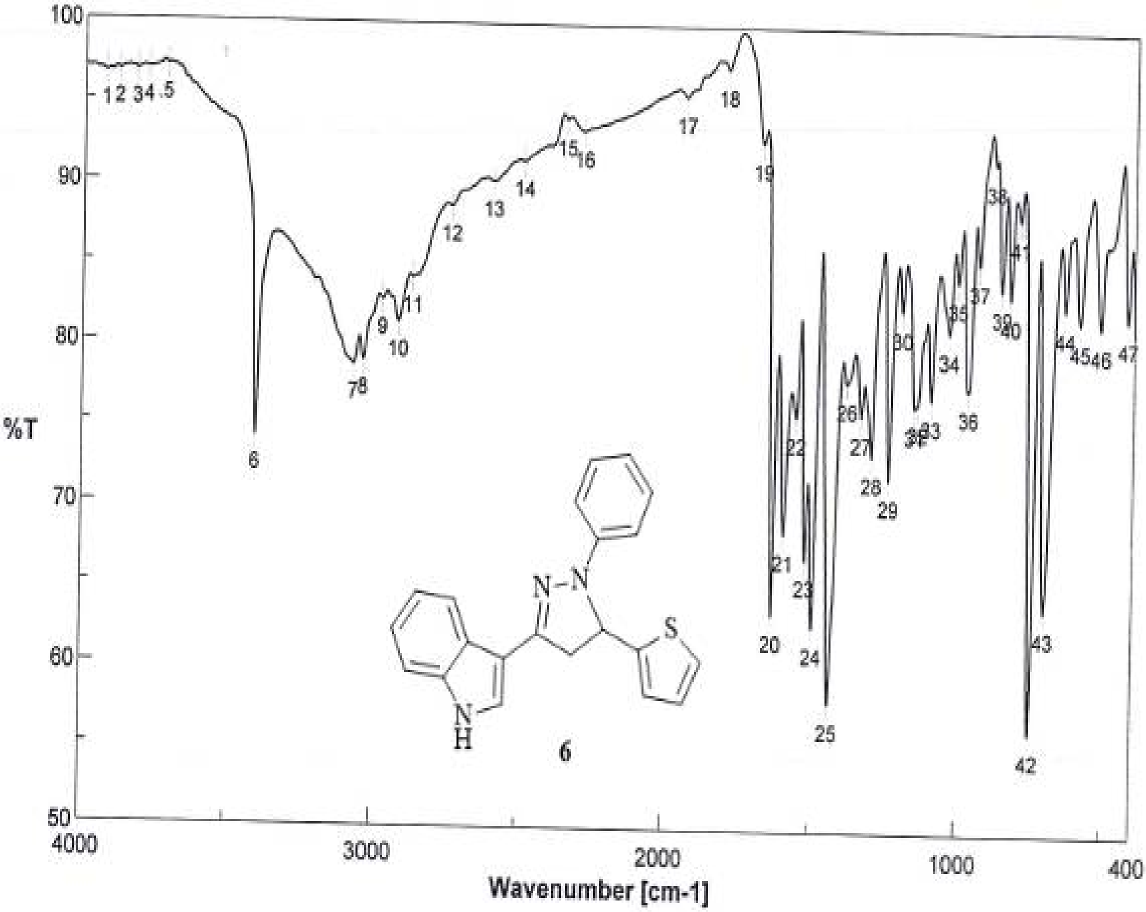

Accumulation	16
Resolution	4 cm-1
Zero Filling	ON
Apodization	Cosine
Gain	Auto (2)
Scanning Speed	Auto (2 mm/sec)
Date/Time	10/20/2020 0:43PM
Update	10/20/2020 0:44PM
Operator	
File Name	Memory#140
Sample Name	W7
Comment	

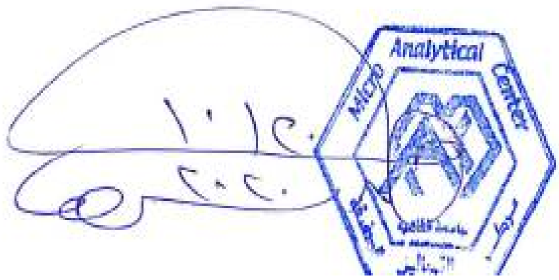

IR spectrum for compound 6

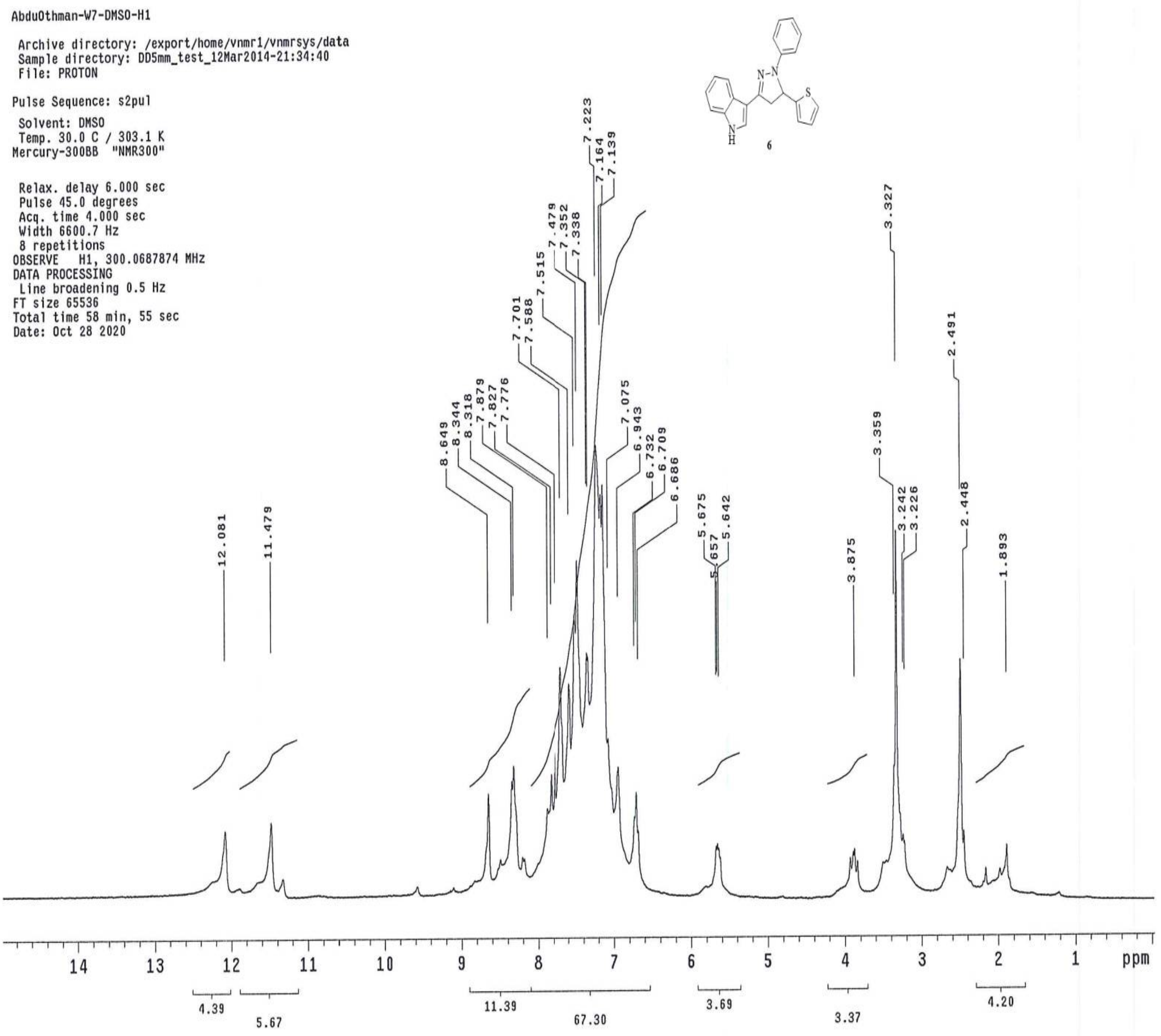

^{1}H-NMR spectrum for compound 6

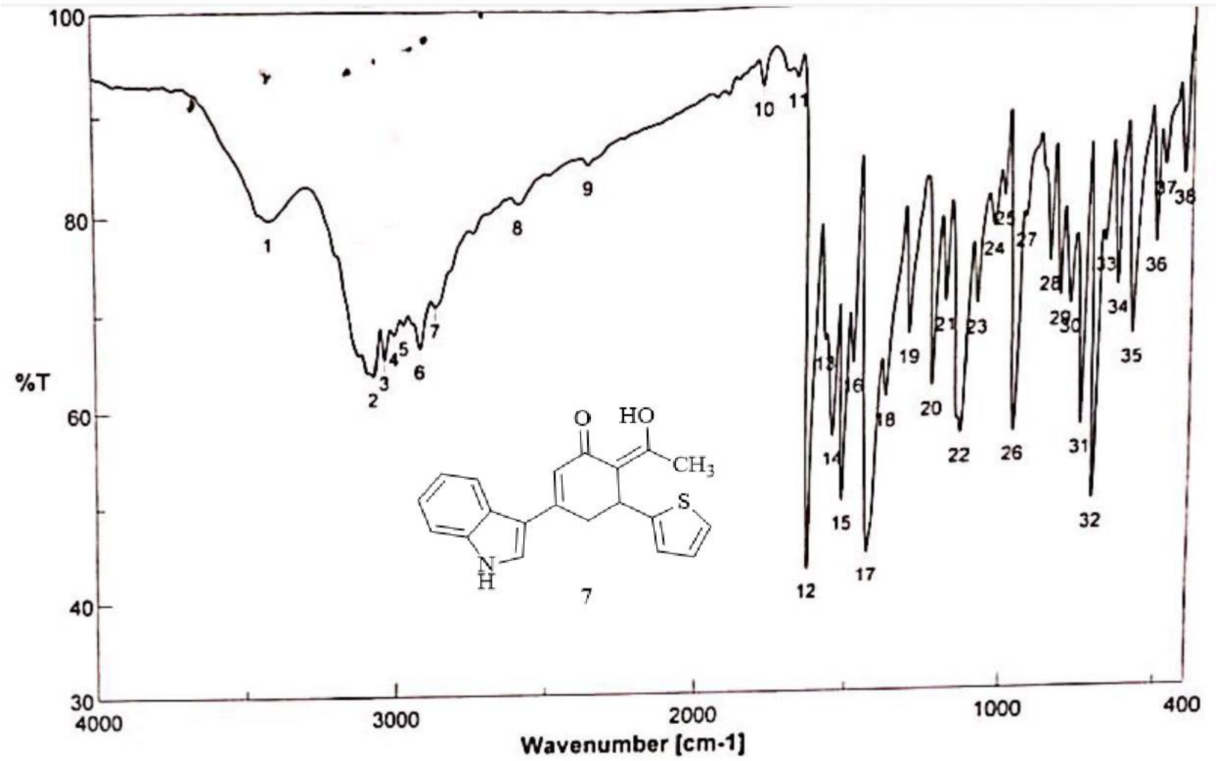

Accumulation	16
Resolution	4 cm-1
Zero Filling	ON
Apodization	Cosine
Gain	Auto (4)
Scanning Speed	Auto (2 mm/sec)
Date/Time	1/13/2021 10:03AM
Update	1/13/2021 10:05AM
Operator	
File Name	Memory#34
Sample Name	w8
Comment	

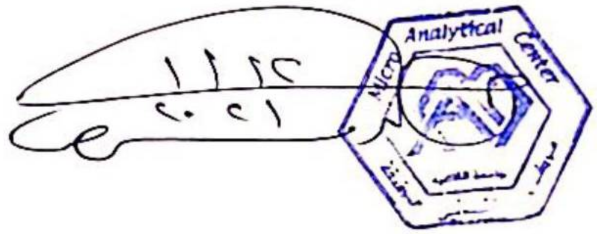

IR spectrum for compound 7

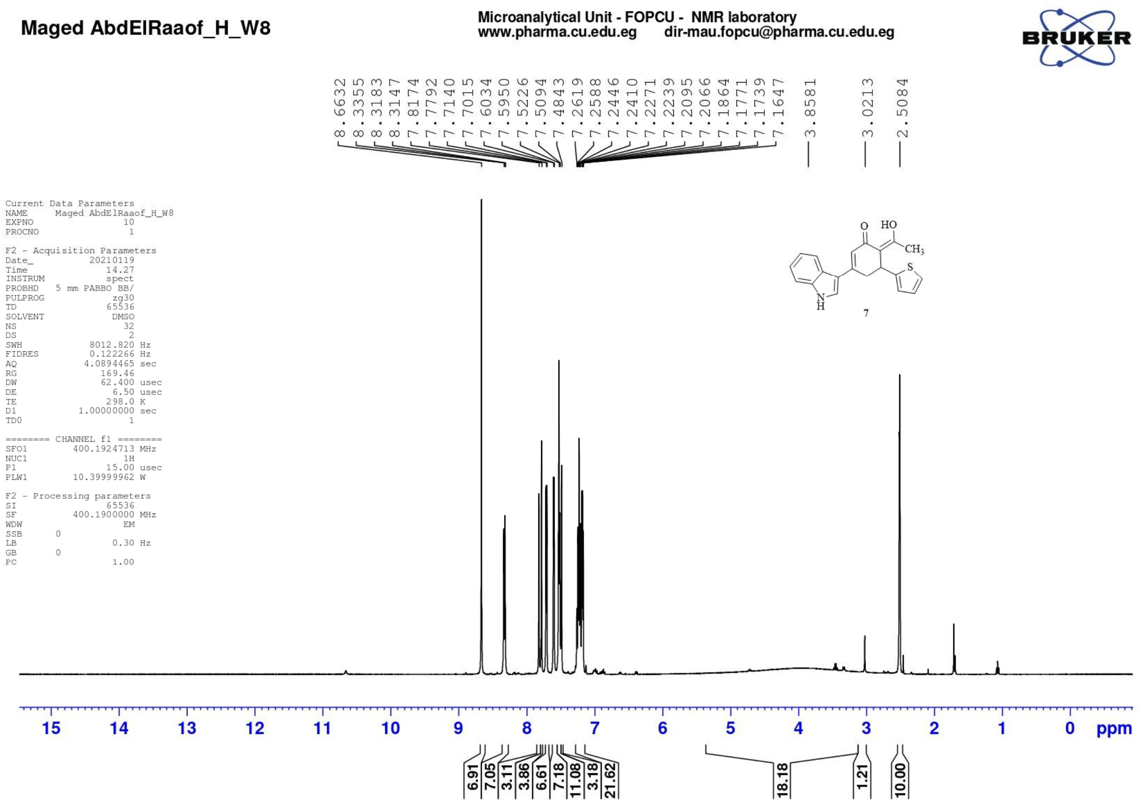

^{1}H-NMR spectrum for compound 7

23-Jan-07

Cairo University
Micro Analytical Center

DI Analysis
Shimadzu Qp-2010 Plus

Sample Information

Analyzed by	: Dr. Mai Younis
Analyzed	: 23/01/2007 07:20:40 م
Sample Name	: W7
Sample ID	:
Customer Name	: Dr. Abdou Othman - Science - Cairo
Data File	: C:\GCMSsolution\Data\Project1\W7..QGD
Org Data File	: C:\GCMSsolution\Data\Project1\W7..QGD
Method File	: C:\GCMSsolution\Data\Project1\High Temperature Op
Org Method File	: C:\GCMSsolution\Data\Project1\High Temperature Op
Report File	:
Tuning File	: C:\GCMSsolution\System\Tune1_default.qgt
SEndIf$Modified by	: Dr. Mai Younis
Modified	: 23/01/2007 07:27:13 م

Method

===== Analytical Line 1 =====

IonSourceTemp	:250.00 °C
[MS Table]	
--Group 1 - Event 1--	
Start Time	:0.00min
End Time	:10.00min
ACQ Mode	:Scan
Event Time	:0.50sec
Scan Speed	:1000
Start m/z	:50.00
End m/z	:500.00
Electron Voltage	: 70 eV
Ionization Mode	: EI

C:\GCMSsolution\Data\Project1\W7..QGD

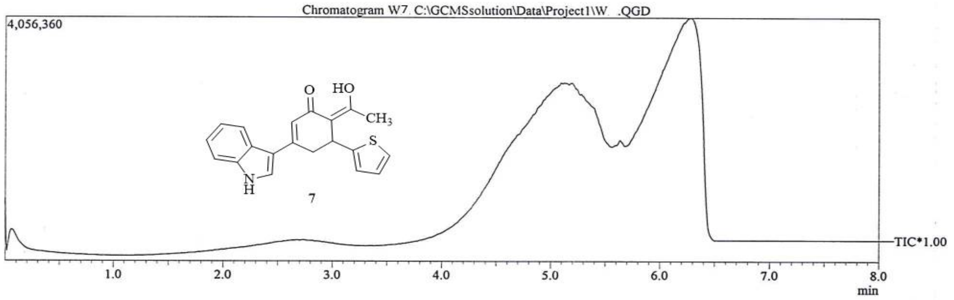

Spectrum

Line#:1 R.Time:5.2(Scan#:619)
MassPeaks:216
RawMode:Single 5.2(619) BasePeak:253(271556)
BG Mode:None Group 1 - Event 1

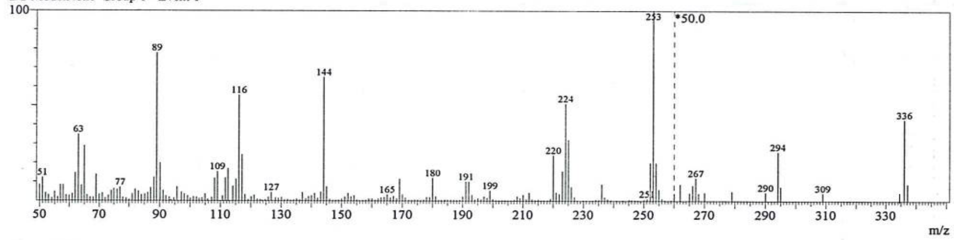

Mass Table
Line#:1 R.Time:5.2(Scan#:619)
MassPeaks:216
RawMode:Single 5.2(619) BasePeak:253(271556)
BG Mode:None Group 1 - Event 1

#	m/z	Abs. In	Rel. Int.	#	m/z	Abs. In	Rel. Int.	#	m/z	Abs. In	Rel. Int.
1	50.00	22758	8.38	4	53.00	8134	3.00	7	56.05	4803	1.77
2	51.00	32999	12.15	5	54.05	3631	1.34	8	57.05	22669	8.35
3	52.05	11053	4.07	6	55.05	12778	4.71	9	58.00	22407	8.25

1 / 2

Mass spectrum for compound 7

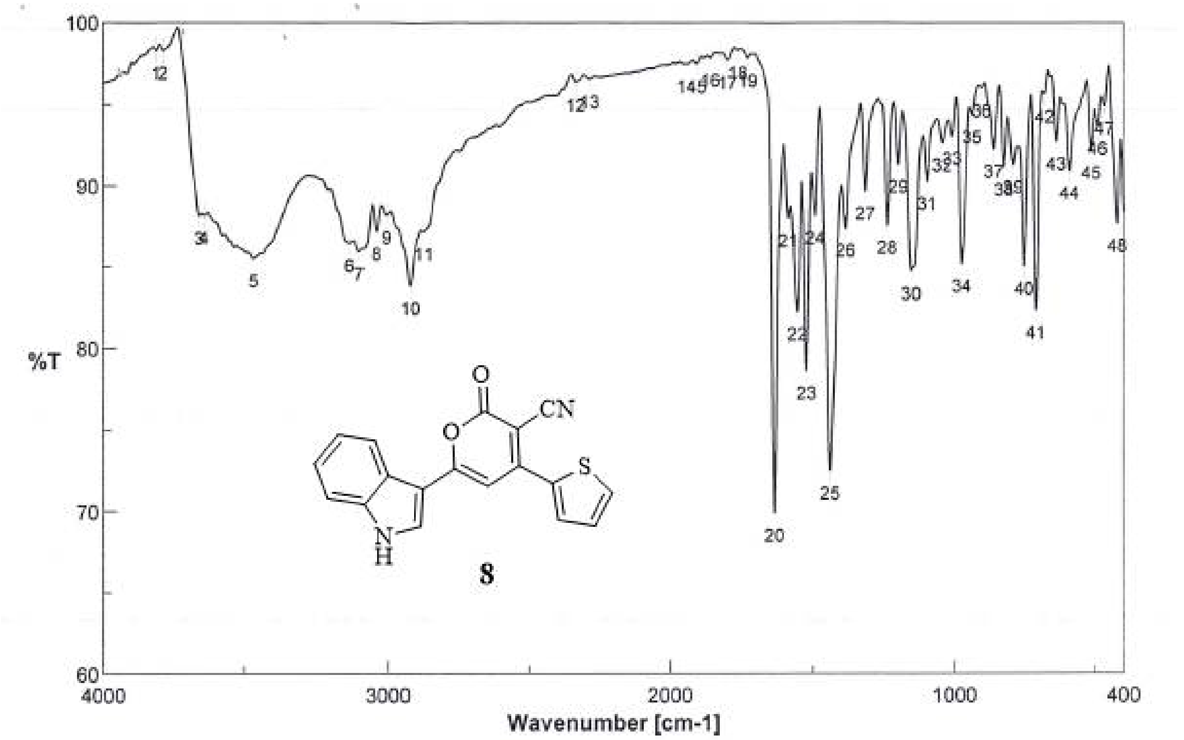

Accumulation	16
Resolution	4 cm-1
Zero Filling	ON
Apodization	Cosine
Gain	Auto (8)
Scanning Speed	Auto (2 mm/sec)
Date/Time	8/27/2020 11:05AM
Update	8/27/2020 11:08AM
Operator	IR
File Name	Memory#52
Sample Name	W2
Comment	

IR spectrum for compound 8

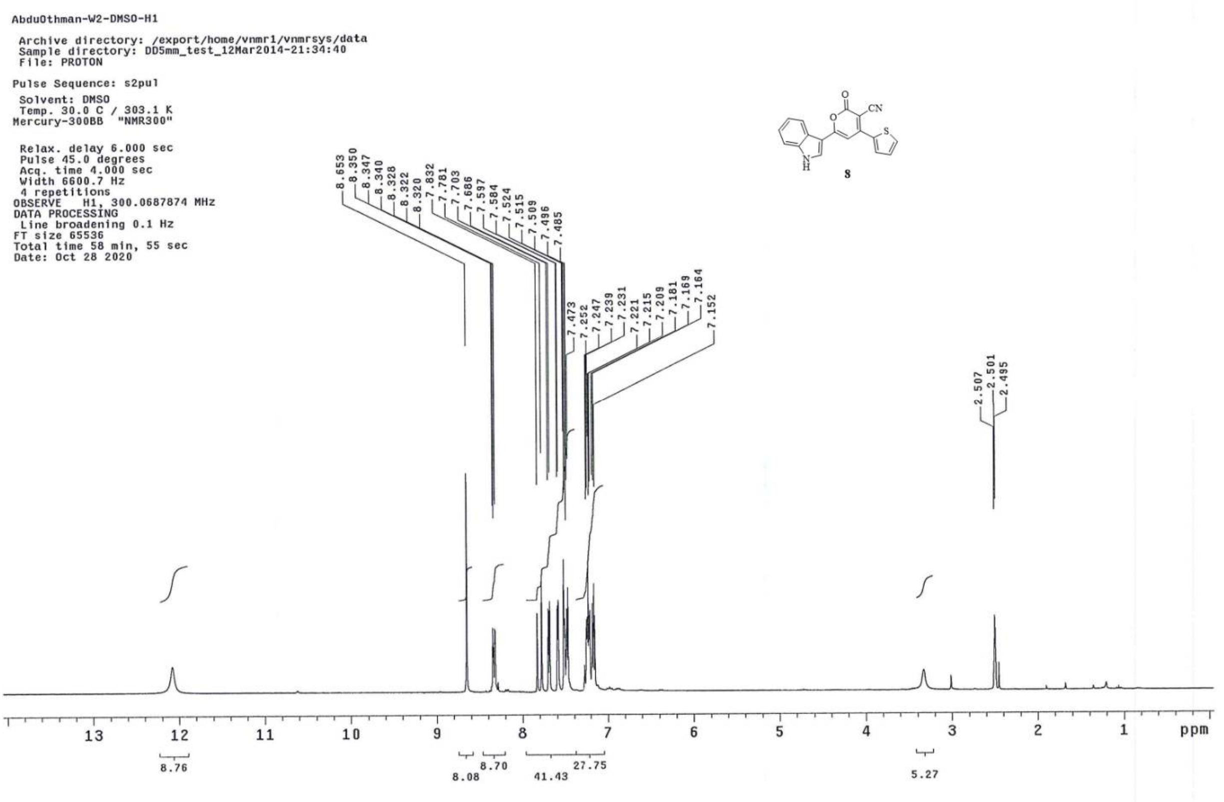

^{1}H-NMR spectrum for compound 8

13-Feb-07

Cairo University
Micro Analytical Center

DI Analysis
Shimadzu Qp-2010 Plus

Sample Information

Analyzed by	: Dr. Mai Younis
Analyzed	: 05/02/2007 04:51:16 م
Sample Name	: W8
Sample ID	:
Customer Name	: Dr. Maged abdelraaof - Science - Zagazik
Data File	: C:\GCMSsolution\Data\Project1\W8 QGD
Org Data File	: C:\GCMSsolution\Data\Project1\W8 QGD
Method File	: C:\GCMSsolution\Data\Project1\High Temperature Op
Org Method File	: C:\GCMSsolution\Data\Project1\High Temperature Op
Report File	:
Tuning File	: C:\GCMSsolution\System\Tune1_default.qgt
$EndIf$Modified by	: Dr. Mai Younis
Modified	: 05/02/2007 04:58:34 م

Method

===== Analytical Line 1 =====

IonSourceTemp	:250.00 °C
[MS Table]	
--Group 1 - Event 1--	
Start Time	:0.00min
End Time	:10.00min
ACQ Mode	:Scan
Event Time	:0.50sec
Scan Speed	:1000
Start m/z	:50.00
End m/z	:500.00
Electron Voltage	: 70 eV
Ionization Mode	: EI

C:\GCMSsolution\Data\Project1\W8 .QGD

Chromatogram W8 C:\GCMSsolution\Data\Project1\W .QGD

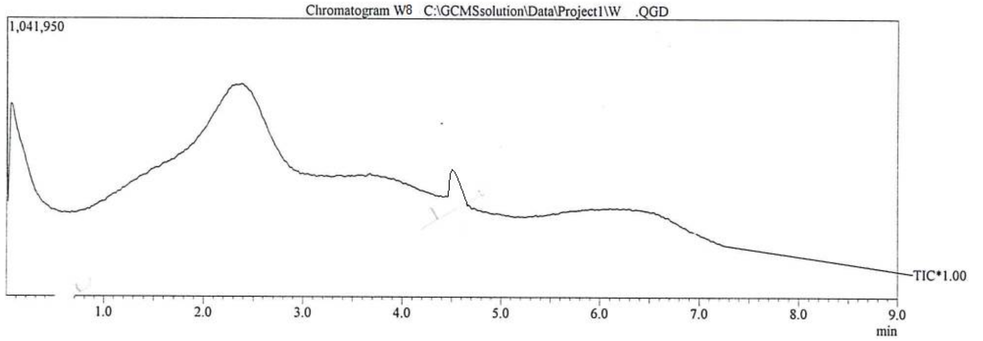

Spectrum

Line#:1 R.Time:6.0(Scan#:724)
MassPeaks:227
RawMode:Single 6.0(724) BasePeak:57(15103)
BG Mode:None Group 1 - Event 1

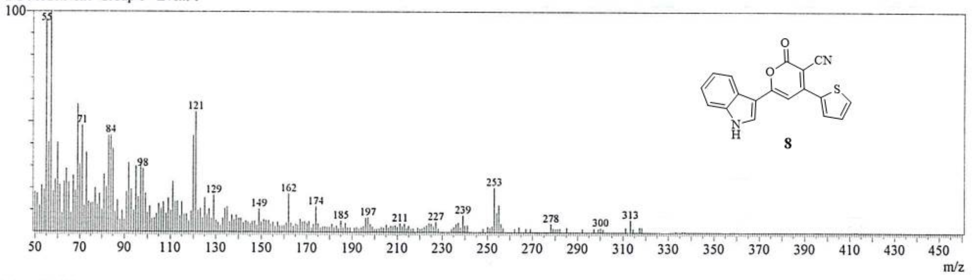

Mass spectrum for compound 9

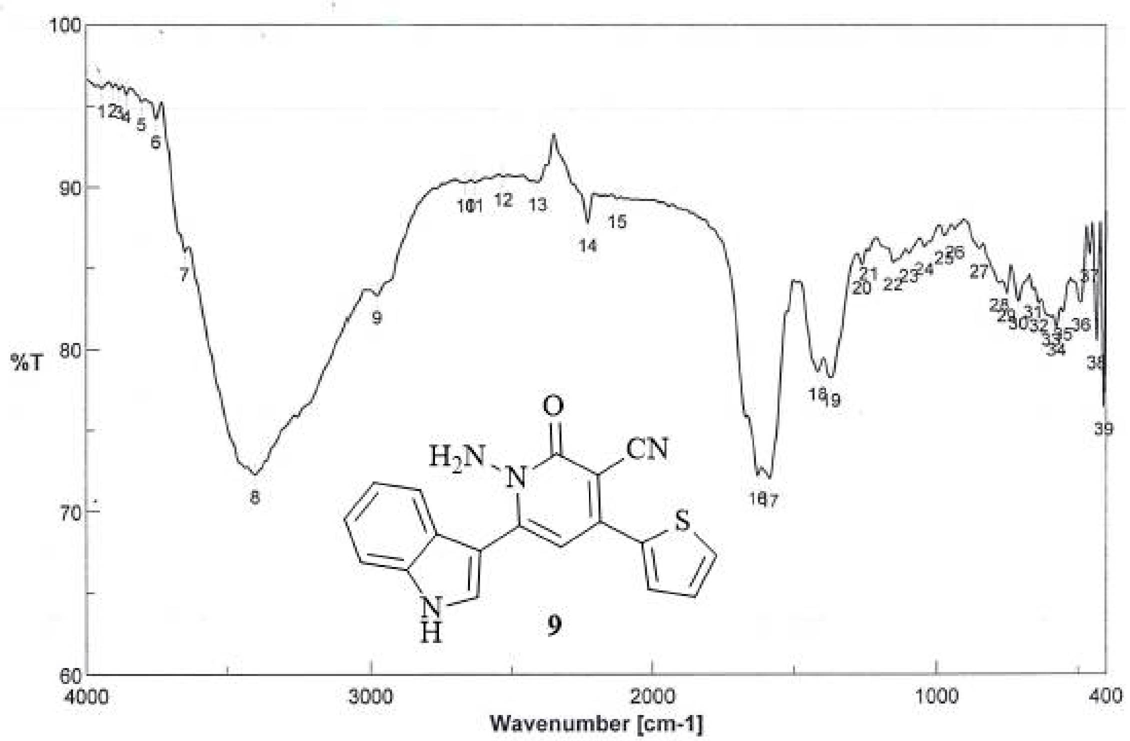

Accumulation	16
Resolution	4 cm-1
Zero Filling	ON
Apodization	Cosine
Gain	Auto (16)
Scanning Speed	Auto (2 mm/sec)
Date/Time	8/27/2020 11:11AM
Update	8/27/2020 11:12AM
Operator	IR
File Name	Memory#64
Sample Name	W3
Comment	

IR spectrum for compound 9

13-Feb-07 20:51:2[illegible]

Cairo University
Micro Analytical Center

DI Analysis
Shimadzu Qp-2010 Plus

Sample Information

Analyzed by : Dr. Mai Younis
Analyzed : 05/02/2007 04:51:16 م
Sample Name : W9
Sample ID :
Customer Name : Dr. Maged abdelraaof - Science - Zagazik
Data File : C:\GCMSsolution\Data\Project1\W9 QGD
Org Data File : C:\GCMSsolution\Data\Project1\W9 QGD
Method File : C:\GCMSsolution\Data\Project1\High Temperature Op
Org Method File : C:\GCMSsolution\Data\Project1\High Temperature Op
Report File :
Tuning File : C:\GCMSsolution\System\Tune1_default.qgt
$EndIf$Modified by : Dr. Mai Younis
Modified : 05/02/2007 04:58:34 م

Method

===== Analytical Line 1 =====
IonSourceTemp :250.00 °C
[MS Table]
--Group 1 - Event 1--
Start Time :0.00min
End Time :10.00min
ACQ Mode :Scan
Event Time :0.50sec
Scan Speed :1000
Start m/z :50.00
End m/z :500.00

Electron Voltage : 70 eV
Ionization Mode : EI

C:\GCMSsolution\Data\Project1\W9 .QGD

Chromatogram W9 C:\GCMSsolution\Data\Project1\W .QGD

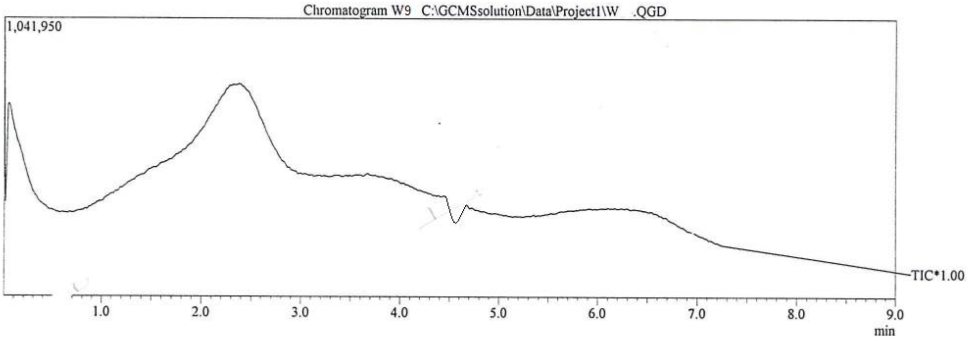

Spectrum

Line#:1 R.Time:6.0(Scan#:724)
MassPeaks:227
RawMode:Single 6.0(724) BasePeak:57(15103)
BG Mode:None Group 1 - Event 1

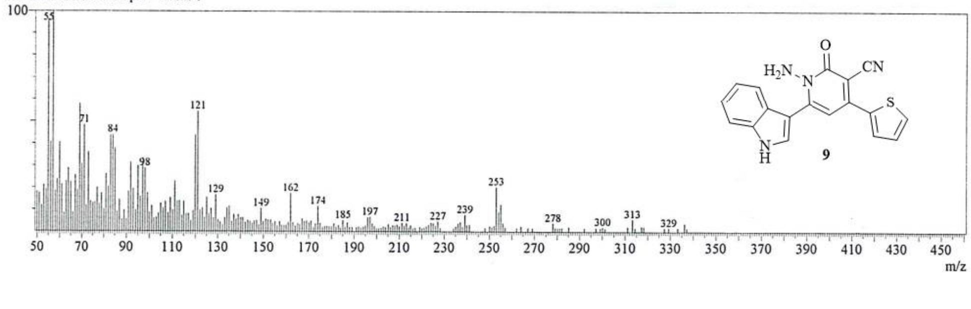

Mass spectrum for compound 9

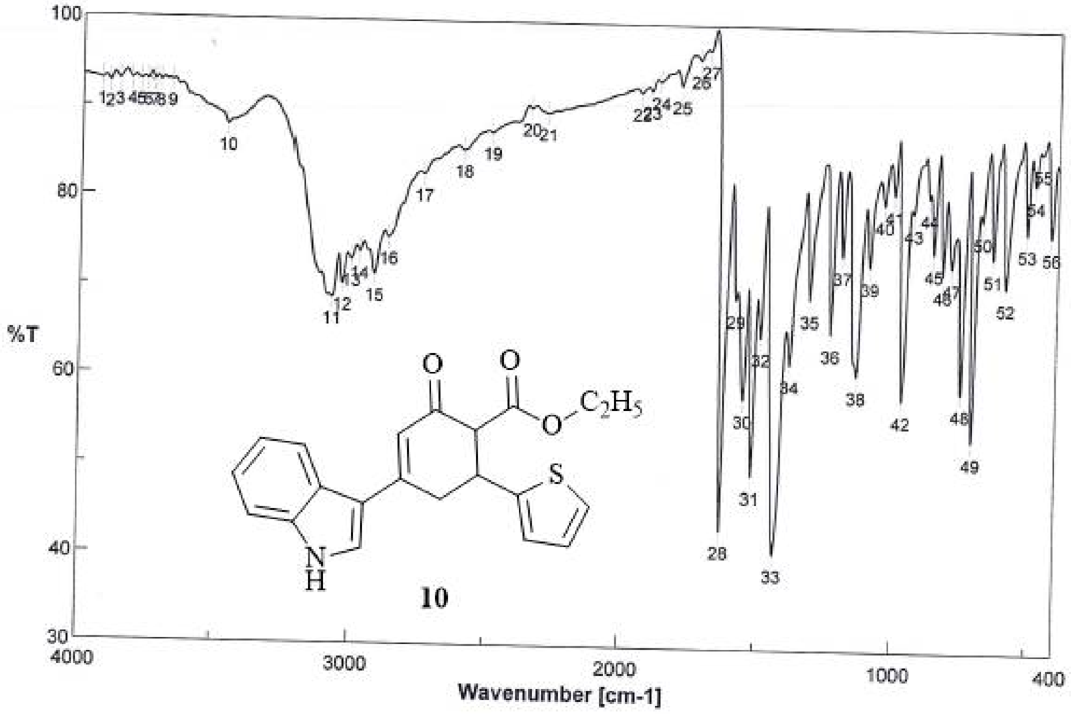

Accumulation	16
Resolution	4 cm-1
Zero Filling	ON
Apodization	Cosine
Gain	Auto (2)
Scanning Speed	Auto (2 mm/sec)
Date/Time	10/20/2020 0:42PM
Update	10/20/2020 0:42PM
Operator	
File Name	Memory#135
Sample Name	W9
Comment	

IR spectrum for compound 10

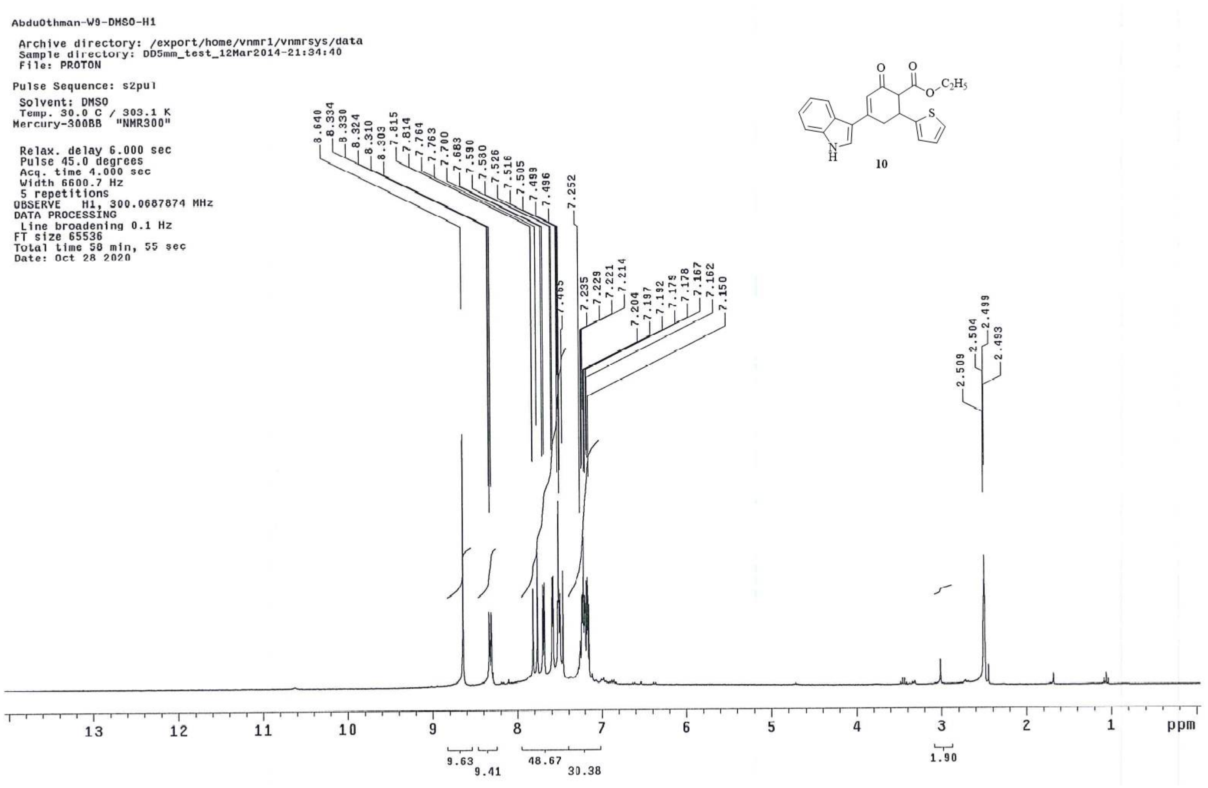

^{1}H-NMR spectrum for compound 10

13-Feb-07 20:51:2[illegible]

Cairo University
Micro Analytical Center

DI Analysis
Shimadzu Qp-2010 Plus

Sample Information

Analyzed by	: Dr. Mai Younis
Analyzed	: 05/02/2007 04:51:16 م
Sample Name	: W10
Sample ID	:
Customer Name	: Dr. Maged abdelraaof - Science - Zagazik
Data File	: C:\GCMSsolution\Data\Project1\W10.QGD
Org Data File	: C:\GCMSsolution\Data\Project1\W10.QGD
Method File	: C:\GCMSsolution\Data\Project1\High Temperature Op
Org Method File	: C:\GCMSsolution\Data\Project1\High Temperature Op
Report File	:
Tuning File	: C:\GCMSsolution\System\Tune1_default.qgt
$EndIf$Modified by	: Dr. Mai Younis
Modified	: 05/02/2007 04:58:34 م

Method

===== Analytical Line 1 =====

IonSourceTemp	:250.00 °C
[MS Table]	
--Group 1 - Event 1--	
Start Time	:0.00min
End Time	:10.00min
ACQ Mode	:Scan
Event Time	:0.50sec
Scan Speed	:1000
Start m/z	:50.00
End m/z	:500.00
Electron Voltage	: 70 eV
Ionization Mode	: EI

C:\GCMSsolution\Data\Project1\W10.QGD

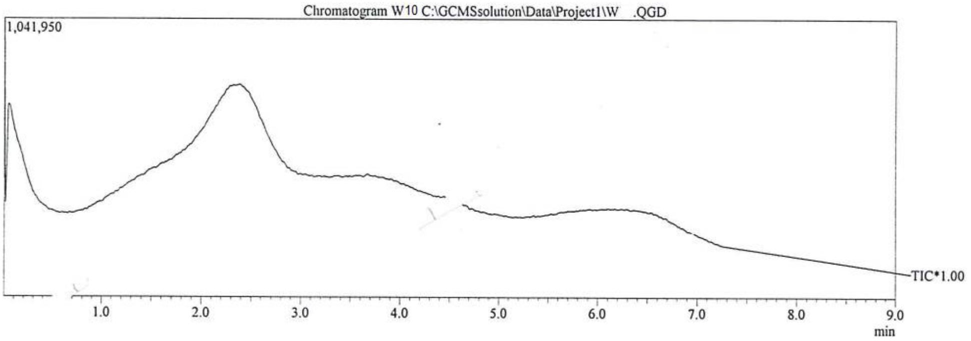

Spectrum

Line#:1 R.Time:6.0(Scan#:724)
MassPeaks:227
RawMode:Single 6.0(724) BasePeak:57(15103)
BG Mode:None Group 1 - Event 1

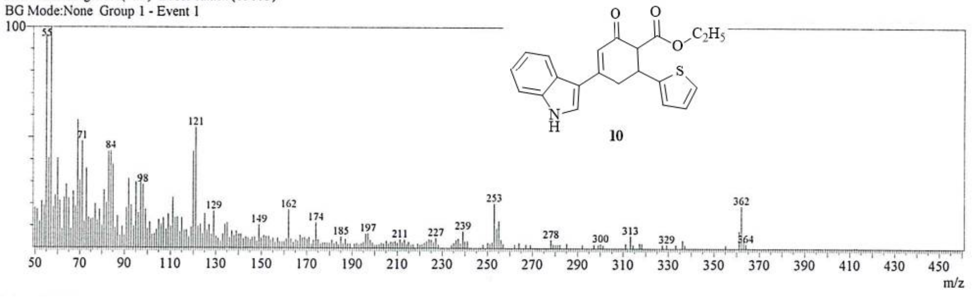

Mass spectrum for compound 10

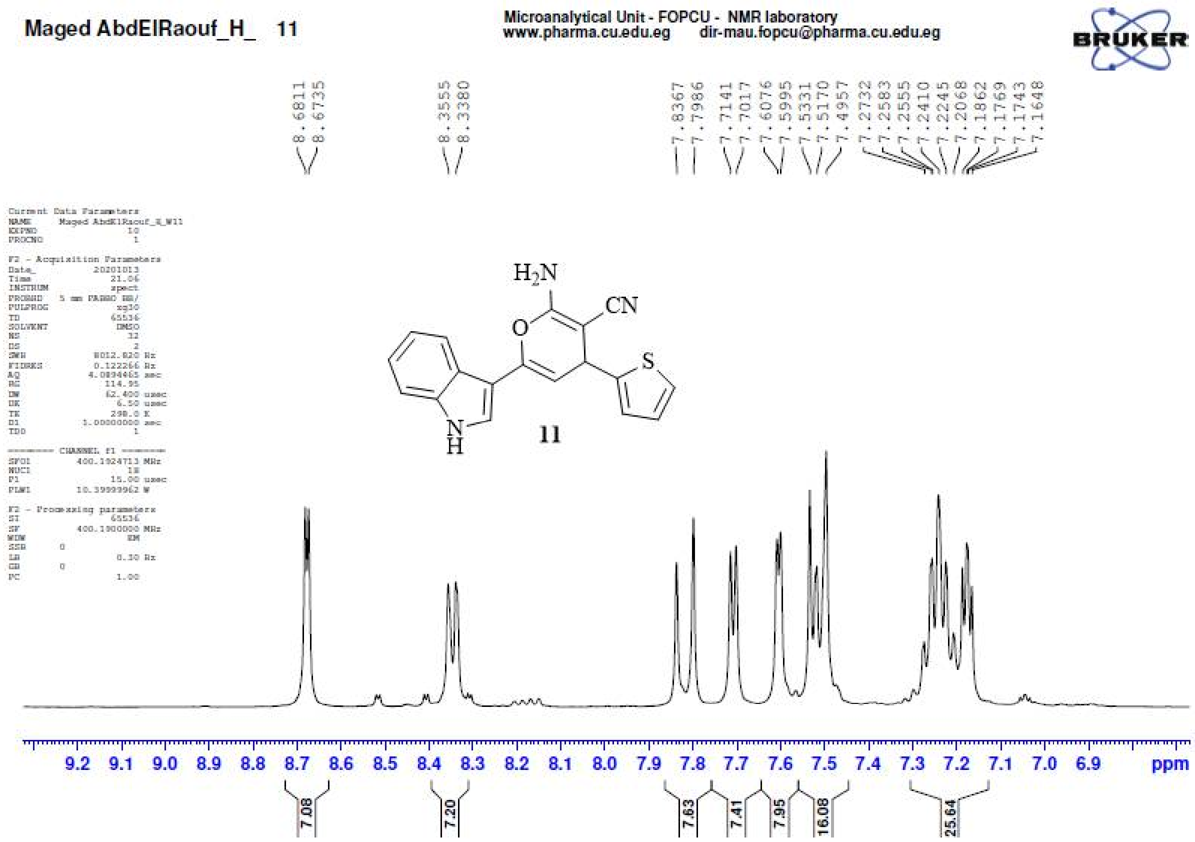

^{1}H-NMR spectrum for compound 11

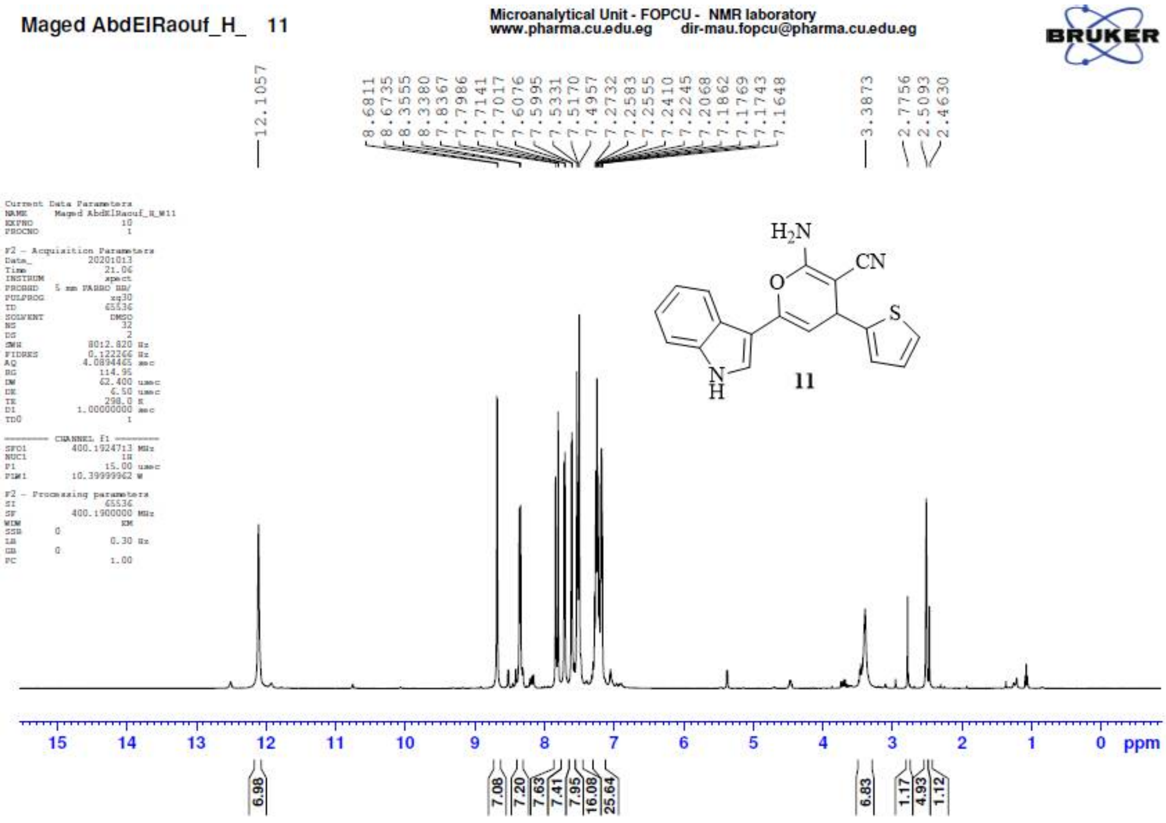

^{1}H-NMR spectrum for compound 11

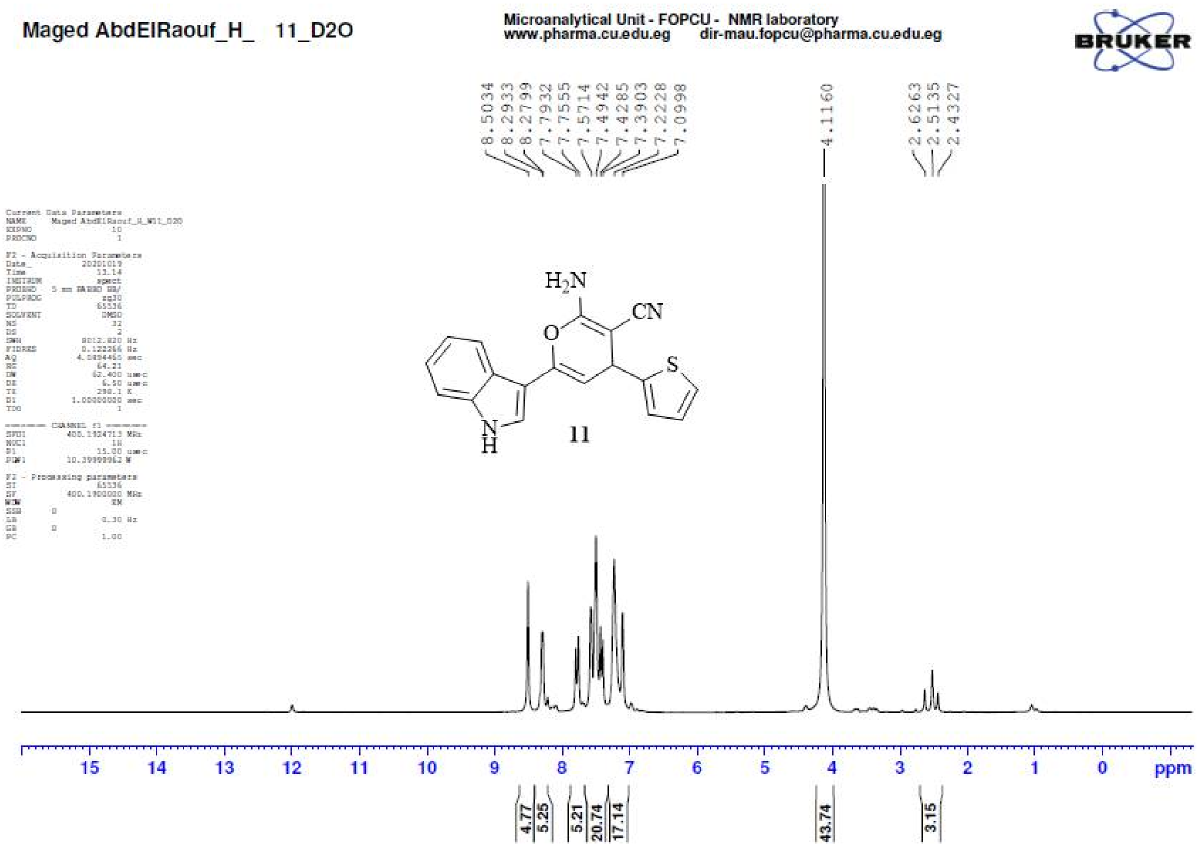

^{1}H-NMR (D_2O) spectrum for compound 11

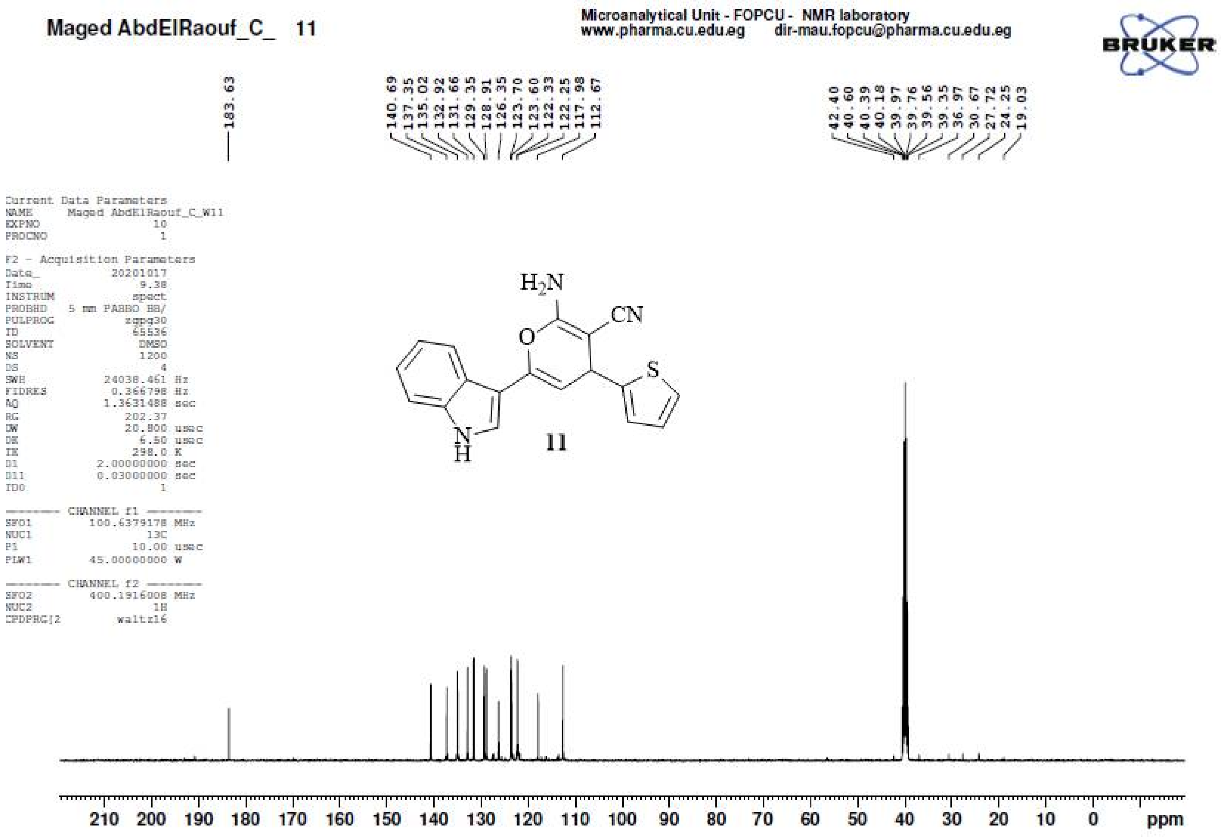

^{13}C-NMR spectrum for compound 11

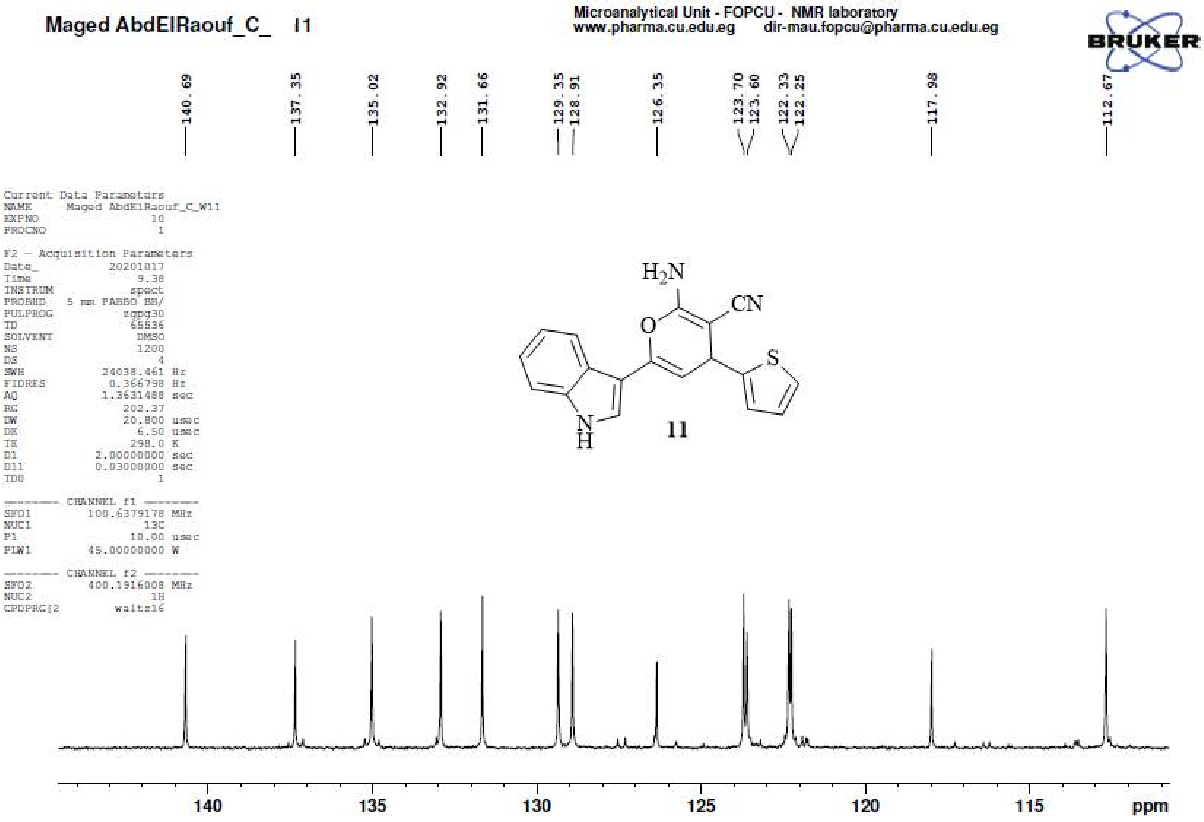

^{13}C-NMR spectrum for compound 11

13-Feb-07

Cairo University
Micro Analytical Center

DI Analysis
Shimadzu Qp-2010 Plus

Sample Information

Analyzed by	: Dr. Mai Younis
Analyzed	: 05/02/2007 04:51:16 م
Sample Name	: W11
Sample ID	:
Customer Name	: Dr. Maged abdelraaof - Science - Zagazik
Data File	: C:\GCMSsolution\Data\Project1\W11.QGD
Org Data File	: C:\GCMSsolution\Data\Project1\W11.QGD
Method File	: C:\GCMSsolution\Data\Project1\High Temperature Op
Org Method File	: C:\GCMSsolution\Data\Project1\High Temperature Op
Report File	:
Tuning File	: C:\GCMSsolution\System\Tune1_default.qgt
$EndIf$Modified by	: Dr. Mai Younis
Modified	: 05/02/2007 04:58:34 م

Method

===== Analytical Line 1 =====

IonSourceTemp	:250.00 °C
[MS Table]	
--Group 1 - Event 1--	
Start Time	:0.00min
End Time	:10.00min
ACQ Mode	:Scan
Event Time	:0.50sec
Scan Speed	:1000
Start m/z	:50.00
End m/z	:500.00
Electron Voltage	: 70 eV
Ionization Mode	: EI

C:\GCMSsolution\Data\Project1\W11 QGD

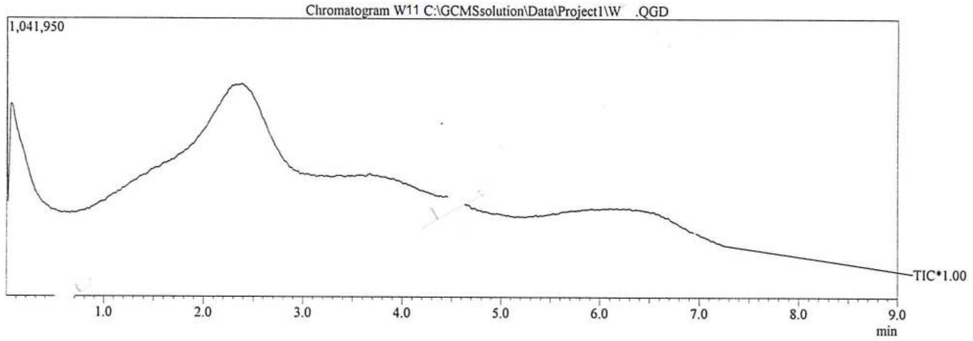

Spectrum

Line#:1 R.Time:6.0(Scan#:724)
MassPeaks:227
RawMode:Single 6.0(724) BasePeak:57(15103)
BG Mode:None Group 1 - Event 1

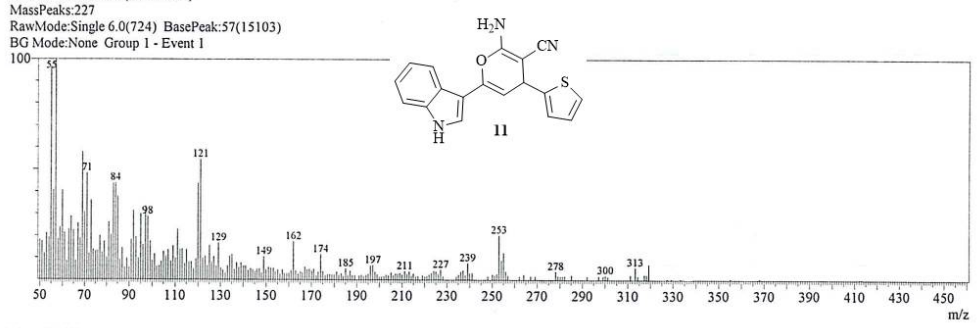

Mass spectrum for compound 11

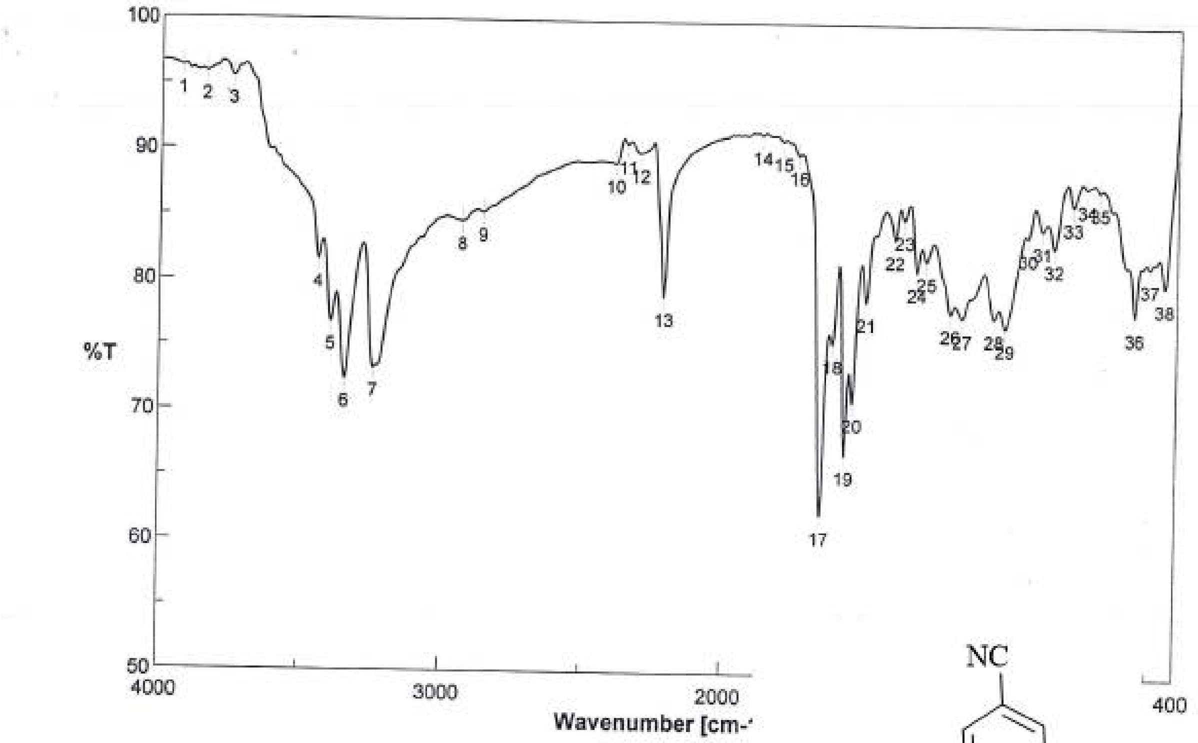

Accumulation	16
Resolution	4 cm-1
Zero Filling	ON
Apodization	Cosine
Gain	Auto (2)
Scanning Speed	Auto (2 mm/sec)
Date/Time	10/20/2020 0:32PM
Update	10/20/2020 0:33PM
Operator	
File Name	Memory#108
Sample Name	W12
Comment	

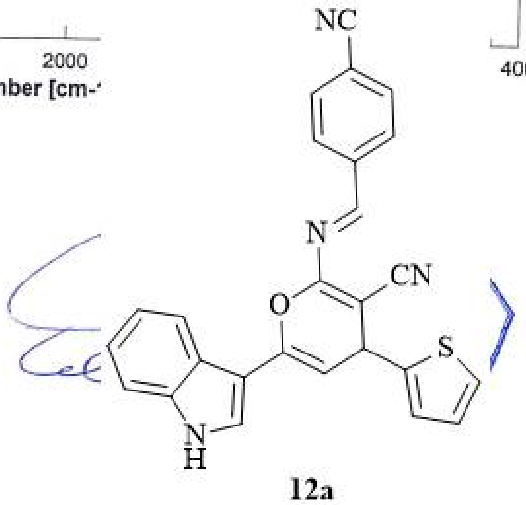

12a

IR spectrum for compound 12a

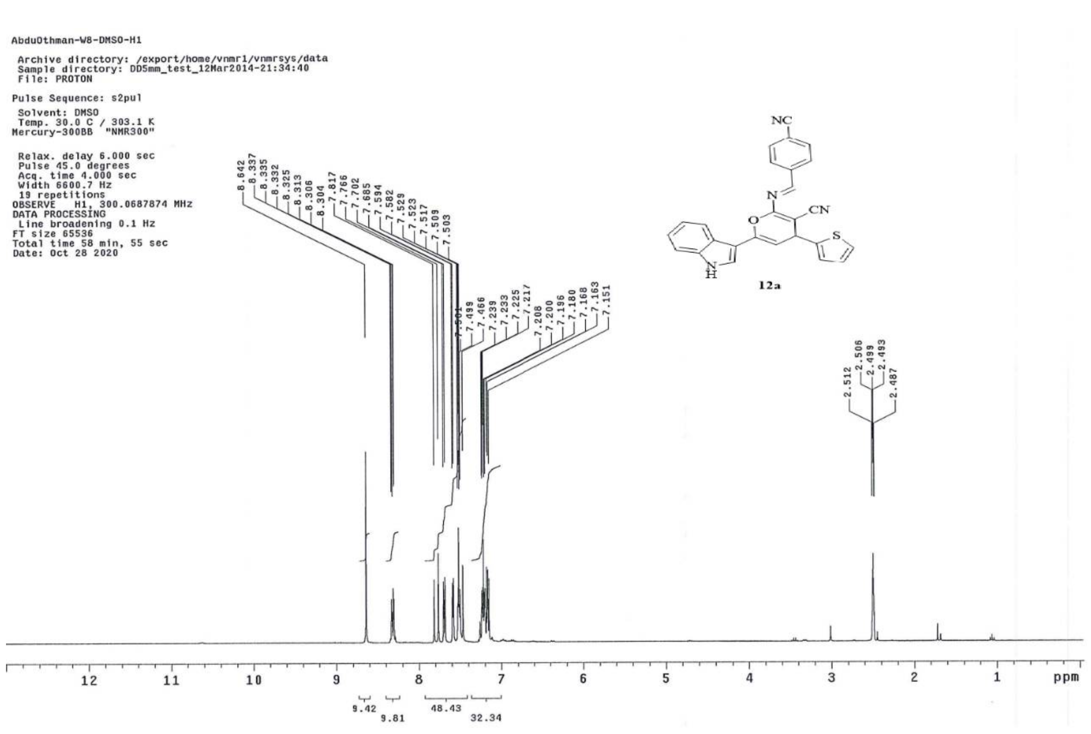

^{1}H-NMR spectrum for compound 12a

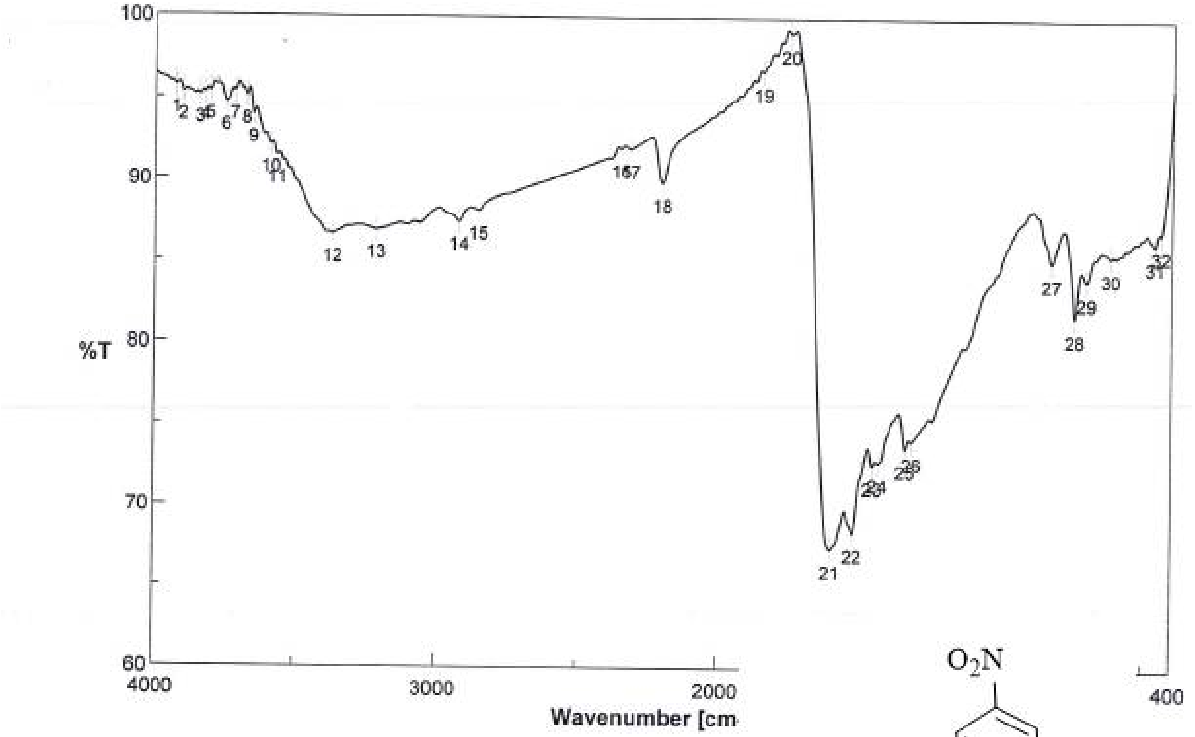

Accumulation	16
Resolution	4 cm-1
Zero Filling	ON
Apodization	Cosine
Gain	Auto (2)
Scanning Speed	Auto (2 mm/sec)
Date/Time	10/20/2020 0:36PM
Update	10/20/2020 0:38PM
Operator	
File Name	Memory#120
Sample Name	W13
Comment	

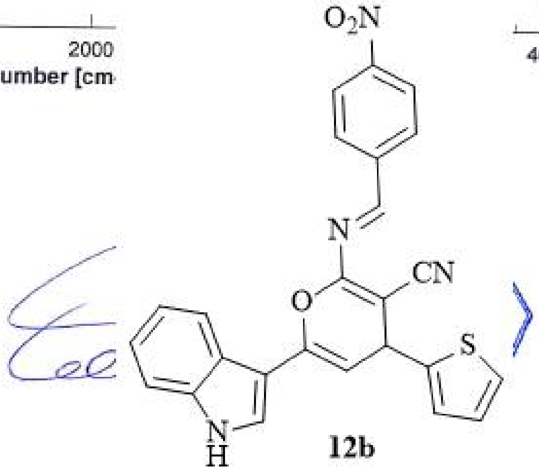

12b

IR spectrum for compound 12b

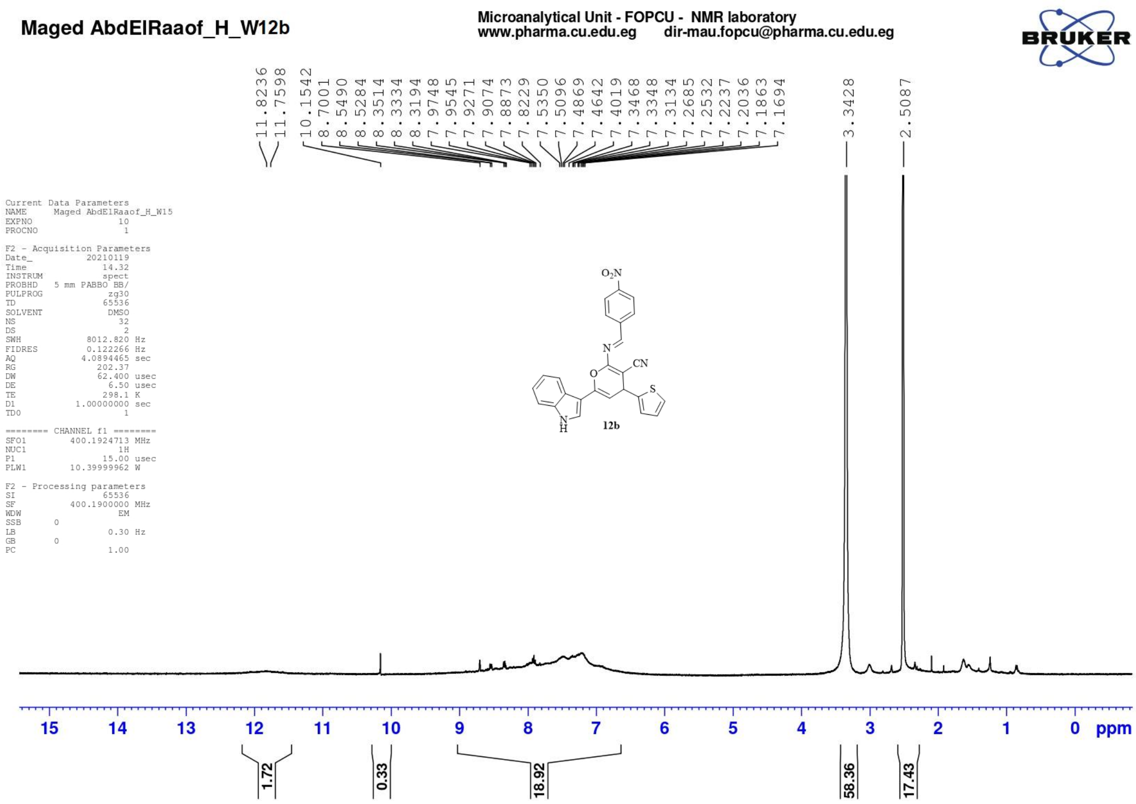

^{1}H-NMR spectrum for compound 12b

23-Jan-07

Cairo University
Micro Analytical Center

DI Analysis
Shimadzu Qp-2010 Plus

Sample Information

Analyzed by : Dr. Mai Younis
Analyzed : 23/01/2007 07:30:04 م
Sample Name : W12b
Sample ID :
Customer Name : Dr. Abdou Othman - Science - Cairo
Data File : C:\GCMSsolution\Data\Project1\W.... QGD
Org Data File : C:\GCMSsolution\Data\Project1\W.... QGD
Method File : C:\GCMSsolution\Data\Project1\High Temperature Op
Org Method File : C:\GCMSsolution\Data\Project1\High Temperature Op
Report File :
Tuning File : C:\GCMSsolution\System\Tune1_default.qgt
$EndIf$Modified by : Dr. Mai Younis
Modified : 23/01/2007 07:38:16 م

Method

===== Analytical Line 1 =====
IonSourceTemp :250.00 °C
[MS Table]
--Group 1 - Event 1--
Start Time :0.00min
End Time :10.00min
ACQ Mode :Scan
Event Time :0.50sec
Scan Speed :1000
Start m/z :50.00
End m/z :500.00

Electron Voltage : 70 eV
Ionization Mode : EI

C:\GCMSsolution\Data\Project1\W ..QGD

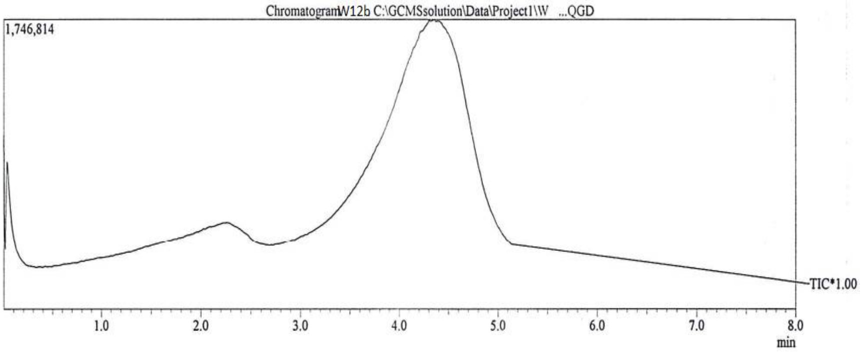

Spectrum

Line#:1 R.Time:4.1(Scan#:497)
MassPeaks:309
RawMode:Single 4.1(497) BasePeak:311(172215)
BG Mode:None Group 1 - Event 1

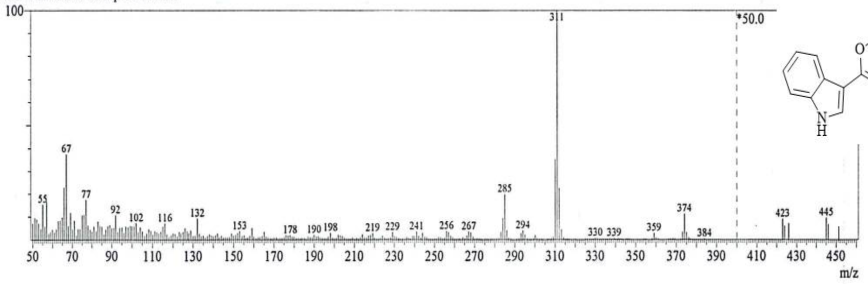

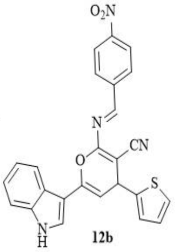

12b

Mass spectrum for compound 12b

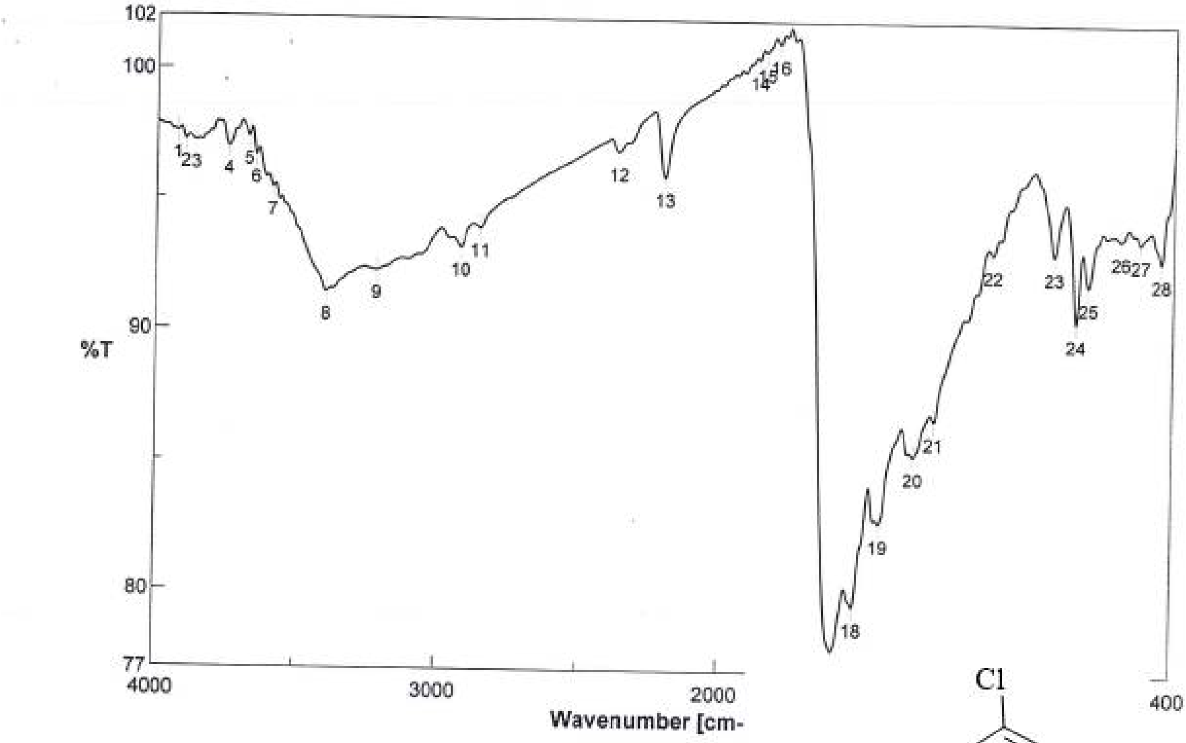

Accumulation	16
Resolution	4 cm-1
Zero Filling	ON
Apodization	Cosine
Gain	Auto (2)
Scanning Speed	Auto (2 mm/sec)
Date/Time	10/20/2020 0:37PM
Update	10/20/2020 0:39PM
Operator	
File Name	Memory#125
Sample Name	W 14
Comment	

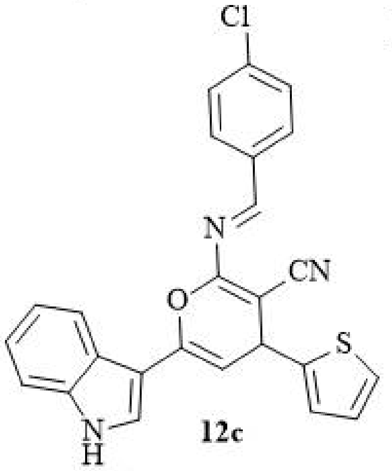

12c

IR spectrum for compound 12c

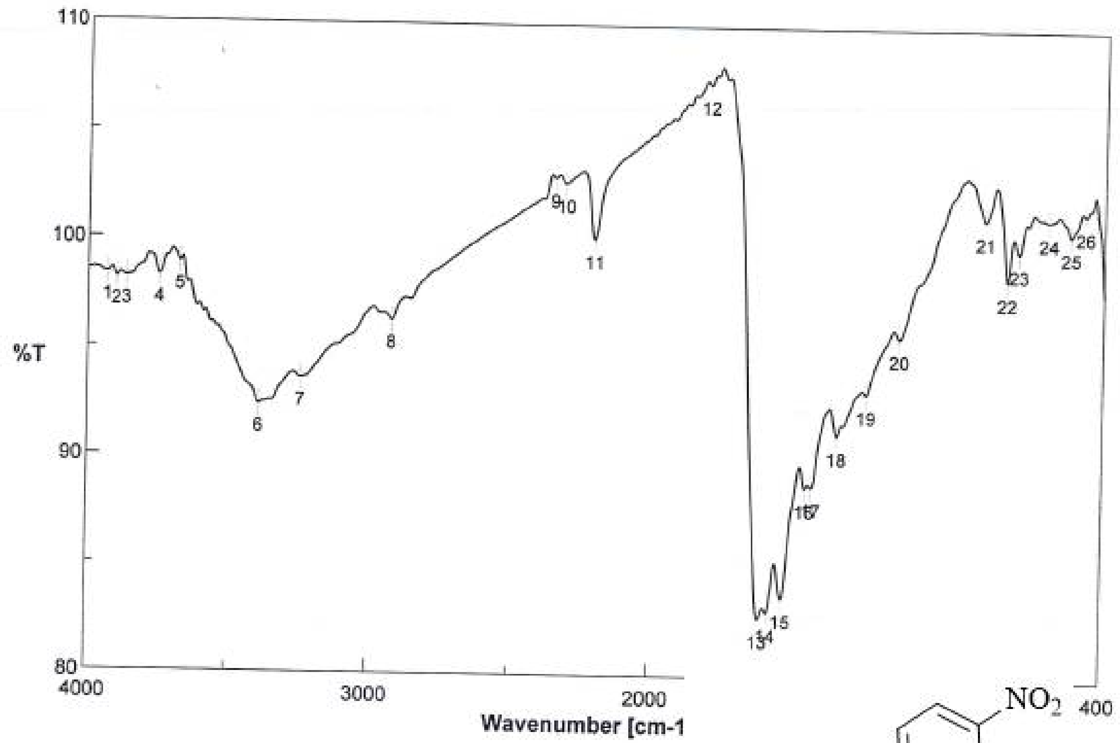

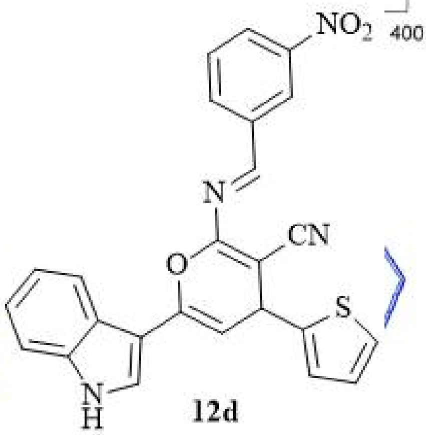

12d

Accumulation	16
Resolution	4 cm-1
Zero Filling	ON
Apodization	Cosine
Gain	Auto (2)
Scanning Speed	Auto (2 mm/sec)
Date/Time	10/20/2020 0:34PM
Update	10/20/2020 0:35PM
Operator	
File Name	Memory#115
Sample Name	W15
Comment	

IR spectrum for compound 12d

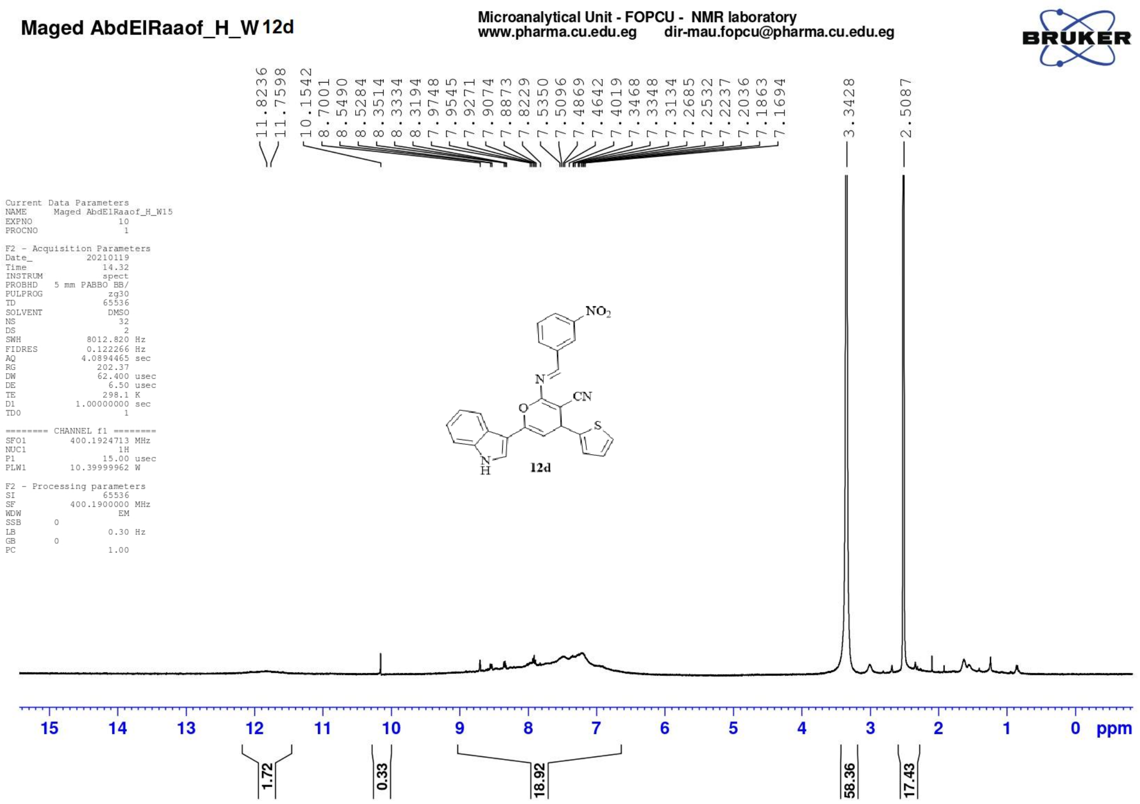

^{1}H-NMR spectrum for compound 12d

23-Jan-07

Cairo University
Micro Analytical Center

DI Analysis
Shimadzu Qp-2010 Plus

Sample Information

Analyzed by	: Dr. Mai Younis
Analyzed	: 23/01/2007 07:33:04 م
Sample Name	: W12d
Sample ID	:
Customer Name	: Dr. Abdou Othman - Science - Cairo
Data File	: C:\GCMSsolution\Data\Project1\W .....QGD
Org Data File	: C:\GCMSsolution\Data\Project1\W ....QGD
Method File	: C:\GCMSsolution\Data\Project1\High Temperature Op
Org Method File	: C:\GCMSsolution\Data\Project1\High Temperature Op
Report File	:
Tuning File	: C:\GCMSsolution\System\Tune1_default.qgt
$EndIf$Modified by	: Dr. Mai Younis
Modified	: 23/01/2007 07:38:16 م

Method

===== Analytical Line 1 =====

IonSourceTemp	:250.00 °C
[MS Table]	
--Group 1 - Event 1--	
Start Time	:0.00min
End Time	:10.00min
ACQ Mode	:Scan
Event Time	:0.50sec
Scan Speed	:1000
Start m/z	:50.00
End m/z	:500.00
Electron Voltage	: 70 eV
Ionization Mode	: EI

C:\GCMSsolution\Data\Project1\W ....QGD

Chromatogram W12dC:\GCMSsolution\Data\Project1\W ...QGD

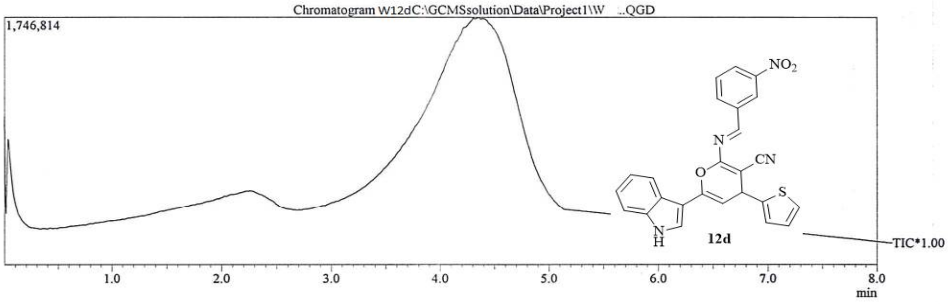

Spectrum

Line#:1 R.Time:4.1(Scan#:497)
MassPeaks:309
RawMode:Single 4.1(497) BasePeak:311(172215)
BG Mode:None Group 1 - Event 1

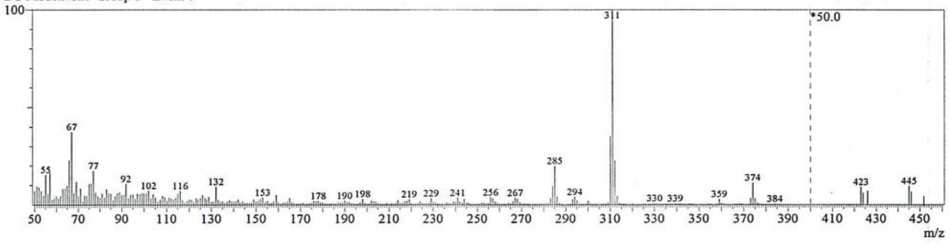

Mass Table
Line#:1 R.Time:4.1(Scan#:497)
MassPeaks:309
RawMode:Single 4.1(497) BasePeak:311(172215)
BG Mode:None Group 1 - Event 1

#	m/z	Abs. In	Rel. Int.	#	m/z	Abs. In	Rel. Int.	#	m/z	Abs. In	Rel. Int.
1	50.00	12046	6.99	4	53.05	11943	6.93	7	56.10	9650	5.60
2	51.05	16019	9.30	5	54.10	7876	4.57	8	57.05	28638	16.63
3	52.05	15057	8.74	6	55.05	26382	15.32	9	58.05	4229	2.46

1 / 3

Mass spectrum for compound 12d

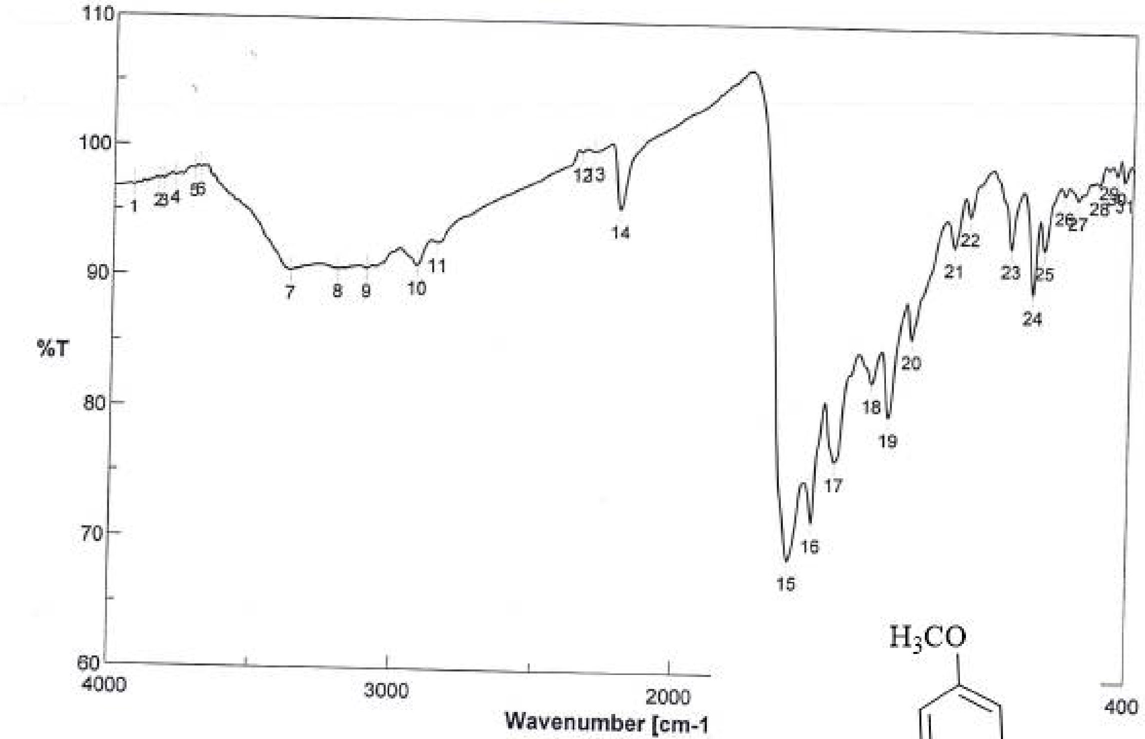

Accumulation	16
Resolution	4 cm-1
Zero Filling	ON
Apodization	Cosine
Gain	Auto (2)
Scanning Speed	Auto (2 mm/sec)
Date/Time	10/20/2020 0:47PM
Update	10/20/2020 0:47PM
Operator	
File Name	Memory#150
Sample Name	W 16
Comment	

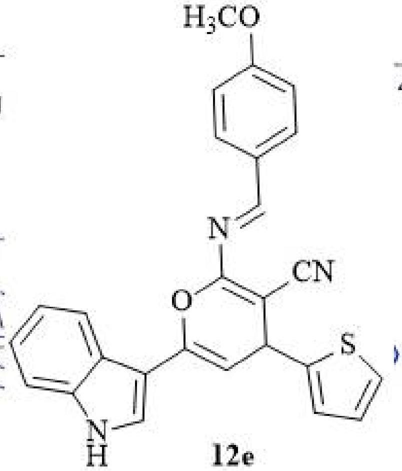

12e

IR spectrum for compound 12e

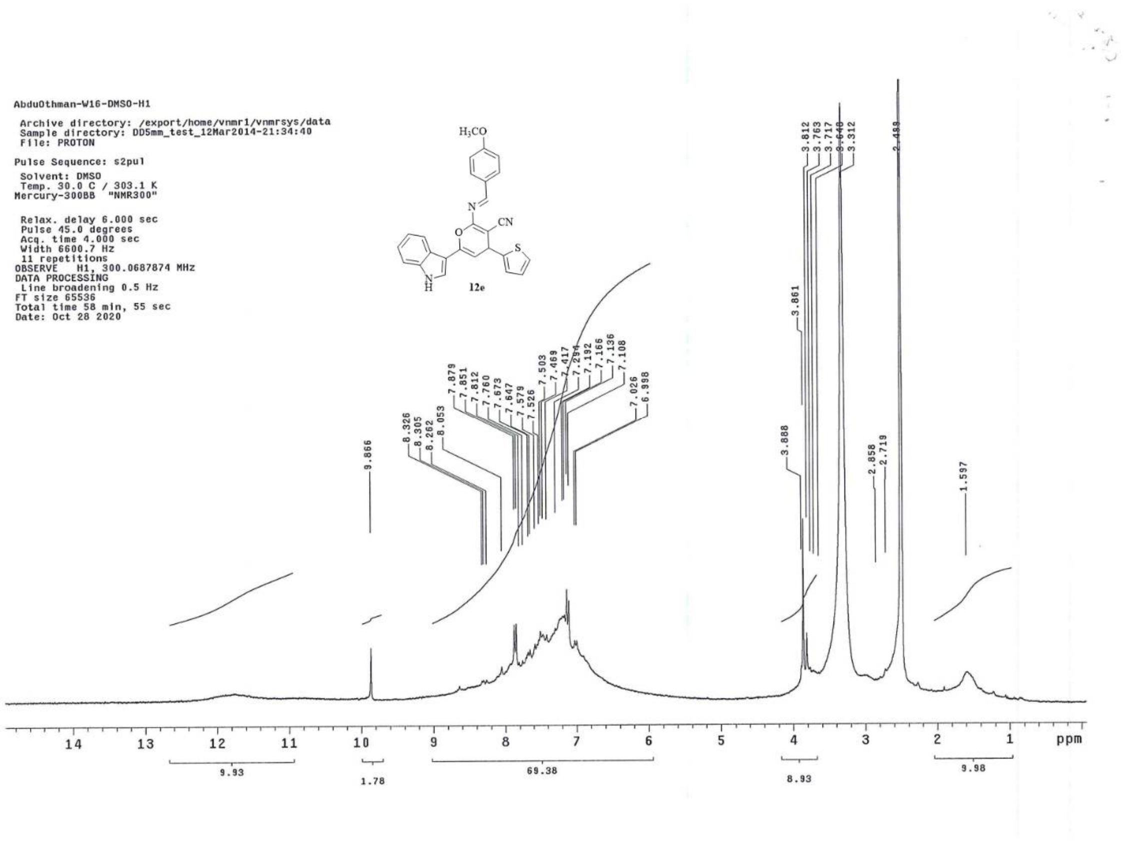

^{1}H-NMR spectrum for compound 12e

23-Jan-07

Cairo University
Micro Analytical Center

DI Analysis
Shimadzu Qp-2010 Plus

Sample Information

Analyzed by : Dr. Mai Younis
Analyzed : 23/01/2007 07:33:0 م
Sample Name : W12e
Sample ID :
Customer Name : Dr. Abdou Othman - Science - Cairo
Data File : C:\GCMSsolution\Data\Project1\W12eQGD
Org Data File : C:\GCMSsolution\Data\Project1\W12eQGD
Method File : C:\GCMSsolution\Data\Project1\High Temperature Op
Org Method File : C:\GCMSsolution\Data\Project1\High Temperature Op
Report File :
Tuning File : C:\GCMSsolution\System\Tune1_default.qgt
$EndIf$Modified by : Dr. Mai Younis
Modified : 23/01/2007 07:38:16 م

Method

===== Analytical Line 1 =====
IonSourceTemp :250.00 °C
[MS Table]
--Group 1 - Event 1--
Start Time :0.00min
End Time :10.00min
ACQ Mode :Scan
Event Time :0.50sec
Scan Speed :1000
Start m/z :50.00
End m/z :500.00

Electron Voltage : 70 eV
Ionization Mode : EI

C:\GCMSsolution\Data\Project1\W12..QGD

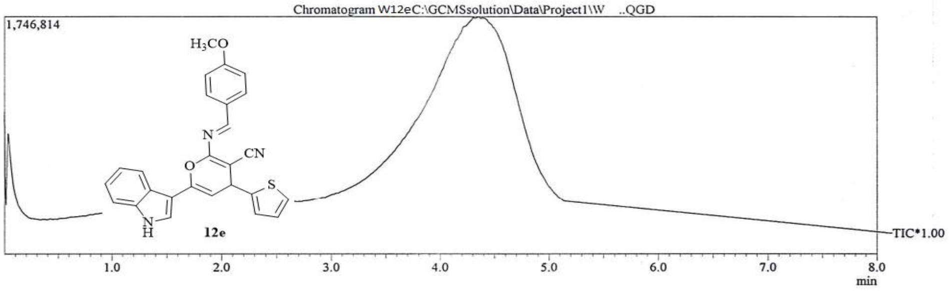

Spectrum

Line#:1 R.Time:4.1(Scan#:497)
MassPeaks:309
RawMode:Single 4.1(497) BasePeak:311(172215)
BG Mode:None Group 1 - Event 1

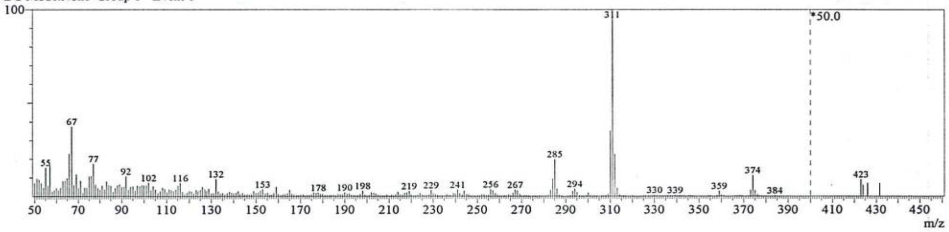

Mass Table
Line#:1 R.Time:4.1(Scan#:497)
MassPeaks:309
RawMode:Single 4.1(497) BasePeak:311(172215)
BG Mode:None Group 1 - Event 1

#	m/z	Abs. In	Rel. Int.	#	m/z	Abs. In	Rel. Int.	#	m/z	Abs. In	Rel. Int.
1	50.00	12046	6.99	4	53.05	11943	6.93	7	56.10	9650	5.60
2	51.05	16019	9.30	5	54.10	7876	4.57	8	57.05	28638	16.63
3	52.05	15057	8.74	6	55.05	26382	15.32	9	58.05	4229	2.46

1 / 3

Mass spectrum for compound 12e
